# Novel benzofuran-based sulphonamides as selective carbonic anhydrases IX and XII inhibitors: synthesis and *in vitro* biological evaluation

**DOI:** 10.1080/14756366.2019.1697250

**Published:** 2019-12-06

**Authors:** Mohamed A. Abdelrahman, Wagdy M. Eldehna, Alessio Nocentini, Hany S. Ibrahim, Hadia Almahli, Hatem A. Abdel-Aziz, Sahar M. Abou-Seri, Claudiu T. Supuran

**Affiliations:** a Department of Pharmaceutical Chemistry, Faculty of Pharmacy, Egyptian Russian University, Badr City, Egypt; b Department of Pharmaceutical Chemistry, Faculty of Pharmacy, Kafrelsheikh University, Kafrelsheikh, Egypt; c Department of NEUROFARBA, Section of Pharmaceutical and Nutraceutical Sciences, University of Florence, Firenze, Italy; d Chemistry Research Laboratory, Department of Chemistry, University of Oxford, Oxford, UK; e Department of Applied Organic Chemistry, National Research Center, Dokki, Egypt; f Department of Pharmaceutical Chemistry, Faculty of Pharmacy, Cairo University, Cairo, Egypt

**Keywords:** Benzenesulfonamides, carbonic anhydrases, benzofurans, synthesis, anticancer

## Abstract

Pursuing on our efforts toward searching for efficient hCA IX and hCA XII inhibitors, herein we report the design and synthesis of new sets of benzofuran-based sulphonamides (**4a**,**b**, **5a**,**b**, **9a–c**, and **10a–d**), featuring the zinc anchoring benzenesulfonamide moiety linked to a benzofuran tail *via* a hydrazine or hydrazide linker. All the target benzofurans were examined for their inhibitory activities toward isoforms hCA I, II, IX, and XII. The target tumour-associated hCA IX and XII isoforms were efficiently inhibited with *K*_I_s spanning in ranges 10.0–97.5 and 10.1–71.8 nM, respectively. Interestingly, arylsulfonehydrazones **9** displayed the best selectivity toward hCA IX and XII over hCA I (SIs: 39.4–250.3 and 26.0–149.9, respectively), and over hCA II (SIs: 19.6–57.1 and 13.0–34.2, respectively). Furthermore, the target benzofurans were assessed for their anti-proliferative activity, according to US-NCI protocol, toward a panel of sixty cancer cell lines. Only benzofurans **5b** and **10b** possessed selective and moderate growth inhibitory activity toward certain cancer cell lines.

## Introduction

Carbonic anhydrases (CAs, EC 4.2.1.1) are metalloenzymes, present in all kingdoms life, catalyse the reversible reaction of the hydration of carbon dioxide to bicarbonate and protons^1^. This simple reaction play a vital role in many physiological and pathological processes associated with pH control, ion transport, and fluid secretion^2–4^. The Zn(II) containing metalloenzyme α-CAs have been reported in vertebrates and, in humans, which is further distinguished by sixteen different hCA isoforms including cytosolic isoforms (hCA I, II, III, VII, and XIII), membrane bound isoforms (hCA IV, IX, XII, XIV, and XV), mitochondrial isoforms (hCA VA and VB) and secreted isoforms (hCA VI) depending upon their distribution in tissues, cellular localisation, and molecular features^5–7^. It is well established that these metalloenzymes possess a significant role in several pathological processes^1^^,^^8–10^. So, modulators of these enzymes could be used as diuretics^11^, anti-glaucoma agents^12^, anti-epileptics^13^, and more recently as antitumor agents^13^^,^^14^. In particular, the human (*h*) isoform CA IX is ectopically expressed in hypoxic tumours, thus acting as a key player in cancer cells survival, proliferation, and metastasis^15^, and its inhibition has been suggested as a promising strategy for treatment of human malignancies^15–17^.

**Figure 1.**
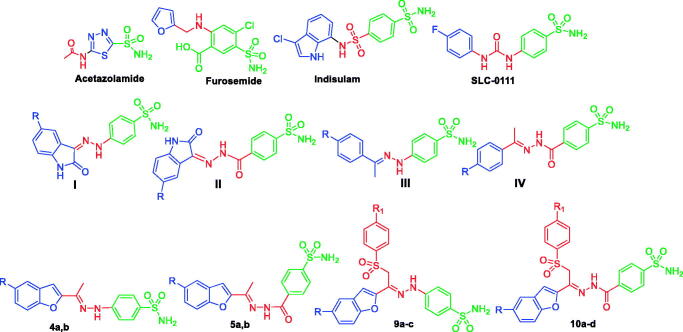
Structures of some CAIs, and the target benzofuran-based sulphonamides **4a, b, 5a, b, 9b–d** and **10a–d**.

Amongst the various classes of CA inhibitors (CAIs), the primary sulphonamides and their bioisosteres represent the most important ones^18^, with many small molecules in clinical use, such as zacetazolamide (AAZ) and furosemide, or in clinical development, such as indisulam and SLC-0111 (Figure 1). Of special interest, SLC-0111 is an ureido-based benzenesulfonamide with selective hCA IX inhibitory activity that is currently being tested in Phase I/II clinical trials for the treatment of advanced hypoxic tumours^13^^,^^19^. Inhibition of CA with the zinc anchoring sulphonamide derivatives is mediated *via* coordination of SO_2_NH^−^ (the deprotonated form) to the positively charged Zn(II) ion in the CA active site. In addition, the sulfamoyl functionality engages two H-bonds: the NH^−^ group acts as donor, while the S = O as acceptor with T199 OG1 atom and backbone NH respectively.

 The “tail approach” is considered to be the most successful approach that could be utilised to afford isoform selective CAIs. In details, the aromatic/heterocyclic ring incorporating the primary sulphonamide functionality, the zinc binding group (ZBG), is to be appended with tail moieties through diverse functionalised linkers. Recently our research team has utilised the tail approach to develop several small molecules as effective CAIs, like structures **I–IV** (Figure 1)^20–24^.

In continuation to our previous effort in the search for efficient hCA IX and hCA XII inhibitors^25–27^, herein we report the design and synthesis of new sets of benzofuran-based sulphonamides (**4a**,**b**, **5a**,**b**, **9a–c**, and **10a–d**, Figure 1), featuring the zinc anchoring benzenesulfonamide moiety linked to a benzofuran tail *via* a hydrazine or hydrazide linker. In series **9** and **10**, an arylsulfone moiety was incorporated to probably promote binding to the hydrophilic part of the active site.

The target benzofurans (**4a**,**b**, **5a**,**b**, **9a–c**, and **10a–d**) were evaluated *in vitro* for their inhibitory activity towards the physiologically relevant hCA isoforms I, II, IX, and XII using stopped-flow CO_2_ hydrase assay. Additionally, they were screened for their anti-proliferative toward a panel of 60 cancer cell lines at dose of 10 mM following the US-NCI single dose assay protocol.

## Materials and methods

### Chemistry

All reaction solvents and reagents were purchased from commercial suppliers and used without further purification. Melting points were measured with a Stuart melting point apparatus and were uncorrected. The NMR spectra were obtained on Bruker Avance 400 (400 MHz ^1^H and 100 MHz ^13^C NMR). ^1^H NMR spectra were referenced to tetramethylsilane (δ = 0.00 ppm) as an internal standard and were reported as follows: chemical shift, multiplicity (b = broad, s = singlet, d = doublet, t = triplet, dd = doublet of doublet, m = multiplet). IR spectra were recorded with a Bruker FT-IR spectrophotometer. Reaction courses and product mixtures were routinely monitored by thin layer chromatography (TLC) that carried out using glass sheets pre-coated with silica gel 60 F_254_ purchased by Merk.

### *General procedure for preparation of compounds* 4a,b and 5a,b

To a solution of 2-acetylbenzofuran derivative **1a** or**1b** (1 mmol) in glacial acetic acid (5 mL), 4-hydrazinylbenzenesulfonamide **2** or 4-(hydrazinecarbonyl)benzenesulfonamide **3** (0.187 g, 1 mmol) was added. The reaction mixture was stirred under reflux temperature for 4 h. The precipitated solid was collected by filtration while hot, washed with cold ethanol, dried and recrystallised from dioxan to afford the target benzofuran-based sulphonamides **4a,b** and **5a,b**, respectively.

#### 4–(2-(1-(Benzofuran-2-yl)ethylidene)hydrazineyl)benzenesulfonamide (4a)

White powder (yield 83%), m.p. 202–205 °C; IR (KBr, ν cm^−1^): 3447 (NH), 3326, 3214 (NH_2_) and 1322, 1147 (SO_2_); ^1^H NMR (DMSO-d_6_) *δ ppm*: 2.33 (s, 3H, CH_3_), 7.15 (s, 1H, Ar-H), 7.24–7.27 (m, 1H, Ar-H), 7.37, 7.39 (2s, 2H, NH_2_ D_2_O exchangeable of -SO_2_NH_2_), 7.52–7.56 (m, 1H, Ar-H), 7.61–7.66 (m, 2H, Ar-H), 7.70–7.74 (m, 2H, Ar-H), 7.83 (d, 1H, *J* = 8.0 Hz, Ar-H), 7.90 (s, 1H, Ar-H), 9.98 (s, 1H, NH D_2_O exchangeable); ^13^C NMR (DMSO-d_6_) *δ ppm*: 13.53, 105.23, 112.74, 114.79, 121.67, 123.64, 124.15, 124.50, 127.75, 128.89, 128.92, 134.70, 135.68, 148.35, 152.59, 155.45; MS *m/z* [%]: 329 [M^+^, 89.27], 89 [100]; Anal. calcd. for C_16_H_15_N_3_O_3_S (329.37): C, 58.35; H, 4.59; N, 12.76. Found C, 58.73; H, 4.53; N, 12.78.

#### 4–(2-(1–(5-Bromobenzofuran-2-yl)ethylidene)hydrazineyl)benzenesulfonamide (4b)

White powder (yield 81%), m.p. >300 °C; IR (KBr, ν cm^−1^): 3434 (NH), 3227, 3316 (NH_2_) and 1343, 1162 (SO_2_); ^1^H NMR (DMSO-d_6_) *δ ppm*: 2.42 (s, 3H, CH_3_), 7.52–7.58 (m, 3H, Ar-H and NH_2_ D_2_O exchangeable of -SO_2_NH_2_), 7.65 (d, 1H, J = 8.0 Hz, Ar-H), 7.92 (s, 1H, Ar-H), 7.95–7.98 (m, 3H, Ar-H), 8.05–8.07 (m, 2H, Ar-H), 11.07 (s, 1H, NH D_2_O exchangeable); ^13^C NMR (DMSO-d_6_) *δ ppm*: 14.52, 108.00, 113.94, 116.13, 124.64, 126.03, 12811, 128.97, 129.30, 130.62, 137.18, 146.41, 147.11, 153.94, 155.30, 163.84; Anal. calcd. for C_16_H_14_BrN_3_O_3_S (408.27): C, 47.07; H, 3.46; N, 10.29. Found C, 47.27; H, 3.49; N, 10.28.

#### 4–(2-(1-(Benzofuran-2-yl)ethylidene)hydrazine-1-carbonyl)benzenesulfonamide (5a)

White powder (yield 80%), m.p. >300 °C; IR (KBr, ν cm^−1^): 3434 (NH), 3316, 3227 (NH_2_), 1682 (C=O) and 1343, 1149 (SO_2_); ^1^H NMR (DMSO-d_6_) *δ ppm*: 2.44 (s, 3H, CH_3_), 7.28 (t, 1H, *J* = 8.0 Hz, Ar-H), 7.39 (t, 1H, *J* = 8.0 Hz, Ar-H), 7.49–7.58 (m, 3H, Ar-H and NH_2_ D_2_O exchangeable of -SO_2_NH_2_), 7.66 (d, 1H, *J* = 8.0 Hz, Ar-H), 7.71 (d, 1H, *J* = 8.0 Hz, Ar-H), 7.96 (d, 2H, *J* = 8.0 Hz, Ar-H), 8.06 (d, 2H, *J* = 8.0 Hz, Ar-H), 11.04 (s, 1H, NH D_2_O exchangeable); ^13^C NMR (DMSO-d_6_) *δ ppm*: 14.57, 108.91, 111.86, 122.33, 123.87, 126.05, 126.14, 128.12, 128.35, 129.26, 136.72, 137.28, 146.58, 147.05, 153.96, 155.16, 163.78; MS *m/z* [%]: 357 [M^+^, 52.05], 184 [100]; Anal. calcd. for C_17_H_15_N_3_O_4_S (357.38): C, 57.13; H, 4.23; N, 11.76. Found C, 57.27; H, 4.29; N, 11.78.

#### 4–(2-(1–(5-Bromobenzofuran-2-yl)ethylidene)hydrazine-1-carbonyl)benzenesulfonamide (5b)

White powder (yield 79%), m.p. 270–272 °C; IR (KBr, ν cm^−1^): 3424 (NH), 3320, 3210 (NH_2_), 1597 (C=O) and 1316, 1150 (SO_2_); ^1^H NMR (DMSO-d_6_) *δ ppm*: 2.31 (s, 3H, CH_3_), 7.16–7.20 (m, 3H, Ar-H and NH_2_ D_2_O exchangeable of -SO_2_NH_2_), 7.37–7.40 (m, 2H, Ar-H),7.42–7.46 (m, 1H, Ar-H), 7.58–7.61 (m, 1H, Ar-H), 7.72–7.75 (m, 2H, Ar-H), 7.83–7.84 (m, 1H, Ar-H), 10.04 (s, 1H, NH D_2_O exchangeable); ^13^C NMR (DMSO-d_6_) *δ ppm*: 13.44, 104.36, 112.85, 113.57, 115.93, 123.93, 127.76, 127.81, 131.22, 134.91, 135.14, 148.19, 153.66, 156.47; Anal. calcd. for C_17_H_14_BrN_3_O_4_S (436.28): C, 46.80; H, 3.23; N, 9.63. Found C, 46.67; H, 3.19; N, 9.70.

### *General procedures for preparation of the target compounds* 9a–c and 10a–d

A mixture of 1-(benzofuran-2-yl)-2-(phenylsulfonyl)ethanone **8a–d** (1 mmol), and 4-hydrazinylbenzenesulfonamide **2** (0.187 g, 1 mmol) or 4-(hydrazinecarbonyl)benzenesulfonamide **3** (0.215 g, 1 mmol) was refluxed in absolute ethanol in the presence of catalytic amount of glacial acetic acid. The solid formed was filtered, dried and recrystallised from ethanol/DMF to afford the target benzofuran-based sulphonamides **9a–c** and **10a–d**, respectively.

#### 4–(2-(1-(Benzofuran-2-yl)-2-tosylethylidene)hydrazineyl)benzenesulfonamide (9a)

Yellow powder (yield 80%), m.p. 269–270 °C; IR (KBr, ν cm^−1^): 3430 (NH), 3309, 3280 (NH_2_) and 1343, 1309, 1265, 1159 (2SO_2_); ^1^H NMR (DMSO-d_6_) *δ ppm*: 2.13, 2.29 (2s, 3H, CH_3_), 4.75, 5.12 (2s, 2H, -SO_2_CH_2_-), 6.97 (d, 1H, *J* = 8.0 Hz, Ar-H), 7.08, 7.77 (s, 1H, Ar-H), 7.16, 7.17 (2s, 2H, NH_2_ D_2_O exchangeable of -SO_2_NH_2_), 7.23–7.29 (m, 2H, Ar- H), 7.31–7.37 (m, 2H, Ar- H), 7.44–7.52 (m, 2H, Ar- H), 7.57–7.73 (m, 3H, Ar-H), 7.74–7.76 (m, 2H, Ar-H), 10.29, 10.55 (2s, 1H, NH D_2_O exchangeable); ^13^C NMR (DMSO-d_6_) *δ ppm*: 21.33, 21.48, 53.15, 60.98, 105.77, 110.04, 111.44, 112.39, 112.98, 113.40, 121.56, 122.46, 123.61, 124.17, 124.25, 125.18, 125.35, 126.59, 127.22, 127.70, 128.73, 128.75, 128.82, 129.89, 129.93, 135.72, 136.00, 136.34, 136.41, 144.80, 145.37, 147.05, 147.38, 148.91, 153.82, 154.23, 154.56; MS *m/z* [%]: 483 [M^+^, 1.26], 143 [100]; Anal. calcd. for C_23_H_21_N_3_O_5_S_2_ (483.56): C, 57.13; H, 4.38; N, 8.69. Found C, 57.19; H, 4.39; N, 8.73.

#### 4–(2-(1–(5-Bromobenzofuran-2-yl)-2-(phenylsulfonyl)ethylidene)hydrazineyl)benzenesulfonamide (9b)

Yellow powder (yield 78%), m.p. 281–283 °C; IR (KBr, ν cm^−1^): 3423 (NH), 3324, 3262 (NH_2_) and 1305, 1263, 1148, 1088 (2SO_2_); ^1^H NMR (DMSO-d_6_) *δ ppm*: 4.82, 5.18 (2s, 2H, -SO_2_CH_2_-), 6.97 (d, 1H, *J* = 8.0 Hz, Ar-H), 7.08, 7.99 (2s, 1H, Ar-H), 7.16, 7.19 (2s, 2H, NH_2_ D_2_O exchangeable of -SO_2_NH_2_), 7.27 (d, 1H, *J* = 8.0 Hz, Ar-H), 7.43–7.56 (m, 2H, Ar-H), 7.58–7.65 (m, 3H, Ar-H), 7.66–7.78 (m, 3H, Ar-H), 7.86 (d, 1H, *J* = 8.0 Hz, Ar-H), 7.91 (d, 1H, *J* = 8.0 Hz, Ar-H), 10.47, 10.62 (2s, 1H, NH D_2_O exchangeable); ^13^C NMR (DMSO-d_6_) *δ ppm*: 52.87, 60.71, 104.84, 109.23, 113.17, 113.46, 113.51, 114.48, 115.95, 116.51, 123.84, 124.31, 124.77, 127.29, 127.77, 128.76, 129.15, 129.55, 129.90, 131.01, 134.16, 134.62, 136.07, 136.21, 139.22, 146.94, 147.26, 150.20, 153.11, 153.35, 155.21; Anal. calcd. for C_22_H_18_BrN_3_O_5_S_2_ (548.43): C, 48.18; H, 3.31; N, 7.66. Found C, 48.39; H, 3.29; N, 7.67.

#### 4–(2-(1–(5-Bromobenzofuran-2-yl)-2-tosylethylidene)hydrazineyl)benzenesulfonamide (9c)

Yellow powder (yield 76%), m.p. 200–202 °C; IR (KBr, ν cm^−1^): 3401 (NH), 3334, 3288 (NH_2_) and 1307, 1265, 1149, 1086 (2SO_2_); ^1^H NMR (DMSO-d_6_) *δ ppm*: 2.13, 2.29 (2s, 3H, CH_3_), 4.74, 5.23 (2s, 2H, -SO_2_CH_2_-), 7.00 (d, 1H, *J* = 8.0 Hz, Ar-H), 7.09, 7.96 (2s, 1H, Ar-H), 7.20 (s, 2H, NH_2_ D_2_O exchangeable of -SO_2_NH_2_), 7.24–7.33 (m, 3H, Ar- H), 7.48 (s, 1H, Ar- H), 7.57 (d, 1H, *J* = 8.0 Hz, Ar-H), 7.63 (d, 2H, *J* = 8.0 Hz, Ar-H), 7.67–7.71 (m, 3H, Ar-H), 10.29, 10.55 (2s, 1H, NH D_2_O exchangeable); ^13^C NMR (DMSO-d_6_) *δ ppm*: 21.31, 21.46, 60.99, 109.22, 113.12, 113.51, 114.47, 115.89, 116.45, 123.58, 123.81, 124.70, 27.23, 127.58, 128.82, 129.08, 129.83, 129.92, 129.94, 131.10, 135.83, 136.21, 136.25, 136.47, 144.82, 145.28, 147.04, 147.28, 150.06, 153.06, 153.35, 155.36; Anal. calcd. for C_23_H_20_BrN_3_O_5_S_2_ (562.45): C, 49.12; H, 3.58; N, 7.47. Found C, 49.29; H, 3.59; N, 7.57.

#### 4–(2-(1-(Benzofuran-2-yl)-2-(phenylsulfonyl)ethylidene)hydrazine-1-carbonyl)benzenesulfonamide (10a)

White powder (yield 80%), m.p. 281–283 °C; IR (KBr, ν cm^−1^): 3429 (NH), 3372, 3310 (NH_2_), 1679 (C=O) and 1343, 1309, 1266, 1159 (2SO_2_); ^1^H NMR (DMSO-d_6_) *δ ppm*: 4.89, 5.43 (2s, 2H, -SO_2_CH_2_-), 7.26 (t, 1H, *J* = 8.0 Hz, Ar-H), 7.37–7.41 (m, 4H, NH_2_ D_2_O exchangeable of -SO_2_NH_2_and Ar-H), 7.54–7.60 (m, 4H, Ar-H), 7.76–7.88 (m, 2H, Ar-H), 7.98–8.06 (m, 5H, Ar-H), 11.32, 11.76 (2s, 1H, NH D_2_O exchangeable); ^13^C NMR (DMSO-d_6_) *δ ppm*: 53.42, 110.06, 111.77, 122.29, 123.94, 126.20, 126.69, 128.15, 128.63, 128.78, 129.23, 129.59, 129.77, 134.51, 134.89, 136.63, 139.02, 147.48, 152.72, 155.03, 163.37; MS *m/z* [%]: 497 [M^+^, 7.32], 77 [100]; Anal. calcd. for C_23_H_19_N_3_O_6_S_2_ (497.54): C, 55.52; H, 3.85; N, 8.45. Found C, 55.23; H, 3.90; N, 8.47.

#### 4–(2-(1-(Benzofuran-2-yl)-2-tosylethylidene)hydrazine-1-carbonyl)benzenesulfonamide (10b)

White powder (yield 78%), m.p. >300 °C; IR (KBr, ν cm^−1^): 3423 (NH), 3370, 3307 (NH_2_), 1680 (C=O) and 1343, 1306, 1267, 1159 (2SO_2_); ^1^H NMR (DMSO-d_6_) *δ ppm*: 2.20, 2.26 (2s, 3H, CH_3_), 4.82, 5.37 (2s, 2H, -SO_2_CH_2_-), 7.27 (d, 2H, *J* = 8.0 Hz, Ar-H), 7.34 (t, 1H, *J* = 8.0 Hz, Ar-H), 7.47, 7.49 (2s, 2H, NH_2_ D_2_O exchangeable of -SO_2_NH_2_), 7.56–7.60 (m, 3H, Ar-H), 7.69–7.71 (m, 2H, Ar-H), 7.73–7.82 (m, 3H, Ar-H), 7.96–8.00 (m, 2H, Ar-H), 11.24, 11.27 (s, 1H, NH D_2_O exchangeable); ^13^C NMR (DMSO-d_6_) *δ ppm*: 19.03, 21.04, 53.57, 110.08, 111.77, 122.27, 123.90, 126.19, 126.67, 128.16, 128.62, 128.80, 129.20, 130.02, 130.18, 134.80, 136.09, 136.54, 145.67, 147.48, 152.69, 154.98, 163.23; MS *m/z* [%]: 511 [M^+^, 5.06], 184 [100]; Anal. calcd. for C_24_H_21_N_3_O_6_S_2_ (511.57): C, 56.35; H, 4.14; N, 8.21. Found C, 56.48; H, 4.18; N, 8.27.

#### 4–(2-(1–(5-Bromobenzofuran-2-yl)-2-(phenylsulfonyl)ethylidene)hydrazine-1-carbonyl)benzenesulfonamide (10c)

White powder (yield 77%), m.p. >300 °C; IR (KBr, ν cm^−1^): 3401 (NH), 3324, 3288 (NH_2_), 1682 (C=O) and 1341, 1307, 1157, 1085 (2SO_2_); ^1^H NMR (DMSO-d_6_) *δ ppm*: 5.26, 5.42 (2s, 2H, -SO_2_CH_2_-), 7.32 (2s, 1H, Ar-H), 7.50–7.55 (m, 3H, NH_2_ D_2_O exchangeable of -SO_2_NH_2_ and Ar-H), 7.57–7.61 (m, 6H, Ar-H),7.87 (s, 1H, Ar-H), 7.95–8.00 (m, 4H, Ar-H), 10.98, 11.36 (2s, 1H, NH D_2_O exchangeable); ^13^C NMR (DMSO-d_6_) *δ ppm*: 53.30, 56.50, 109.03, 113.85, 116.21, 124.60, 126.19, 128.78, 129.31, 129.80, 130.36, 134.95, 136.50, 138.90, 153.76, 153.98, 163.36; Anal. calcd. for C_23_H_18_BrN_3_O_6_S_2_ (576.44): C, 47.92; H, 3.15; N, 7.27. Found C, 47.79; H, 3.19; N, 7.33.

#### 4–(2-(1–(5-Bromobenzofuran-2-yl)-2-tosylethylidene)hydrazine-1-carbonyl)benzenesulfonamide (10d)

White powder (yield 74%), m.p. 283–285 °C; IR (KBr, ν cm^−1^): 3405 (NH), 3323, 3280 (NH_2_), 1673 (C=O) and 1343, 1305,1159, 1086 (2SO_2_); ^1^H NMR (DMSO-d_6_) *δ ppm*: 2.20, 2.25 (2s, 3H, CH_3_), 4.83, 5.37 (2s, 2H, -SO_2_CH_2_-), 7.25–7.31 (m, 3H, Ar-H), 7.49–7.60 (m, 3H, NH_2_ D_2_O exchangeable of -SO_2_NH_2_ and Ar-H), 7.62–7.72 (m, 2H, Ar-H), 7.78 (d, 1H, *J* = 8.0 Hz, Ar-H), 7.86 (s, 1H, Ar-H), 7.96–8.10 (m, 4H, Ar-H), 11.30, 11.74 (2s, 1H, NH D_2_O exchangeable); ^13^C NMR (DMSO-d_6_) *δ ppm*: 21.38, 53.52, 109.04, 113.84, 116.17, 124.58, 126.17, 126.38, 128.69, 128.80, 129.11, 130.19, 130.40, 134.34, 136.06, 145.67, 147.52, 153.73, 153.99, 163.34; Anal. calcd. for C_23_H_20_BrN_3_O_6_S_2_ (590.46): C, 48.82; H, 3.41; N, 7.12. Found C, 48.92; H, 3.34; N, 7.29.

### CA inhibitory assay

An Applied Photophysics stopped-flow instrument has been used for assaying the CA catalysed CO_2_ hydration activity^28^. Phenol red (at a concentration of 0.2 mM) has been used as indicator, working at the absorbance maximum of 557 nm, with 20 mM Hepes (pH 7.5) as buffer, and 20 mM Na_2_SO_4_ (for maintaining constant the ionic strength), following the initial rates of the CA-catalysed CO_2_ hydration reaction for a period of 10–100 s. The CO_2_ concentrations ranged from 1.7 to 17 mM for the determination of the kinetic parameters and inhibition constants. For each inhibitor at least six traces of the initial 5–10% of the reaction have been used for determining the initial velocity. The uncatalyzed rates were determined in the same manner and subtracted from the total observed rates. Stock solutions of inhibitor (0.1 mM) were prepared in distilled-deionised water and dilutions up to 0.01 nM were done thereafter with the assay buffer. Inhibitor and enzyme solutions were preincubated together for 15 min at room temperature prior to assay, in order to allow for the formation of the E-I complex. The inhibition constants were obtained by non-linear least-squares methods using PRISM 3 and the Cheng–Prusoff equation, as reported earlier^22^^,^^23^^,^^29–31^, and represent the mean from at least three different determinations.

### Anti-proliferative activity against sixty NCI-cancer cell lines panel

The anti-proliferative assay was carried out in accordance with the protocol of the Drug Evaluation Branch, NCI, Bethesda^32^^,^^33^, as described previously^34^^,^^35^.

## Results and discussion

### Chemistry

**Scheme 1.**
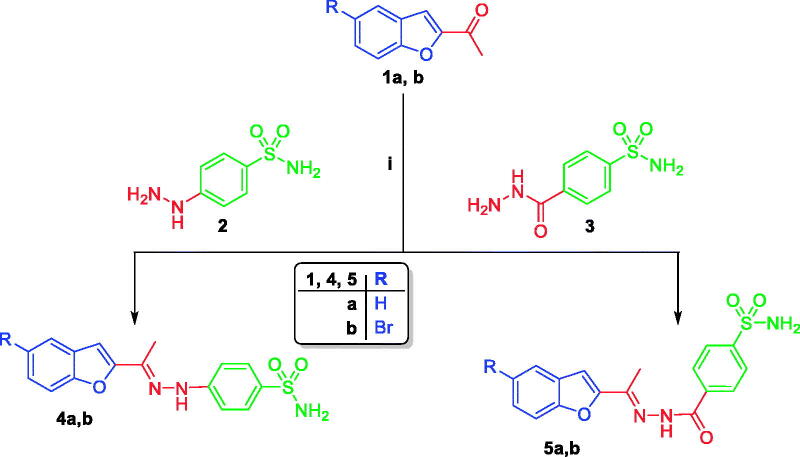
Reagent and conditions: (i) Glacial Acetic acid, reflux 4 h.

**Scheme 2.**
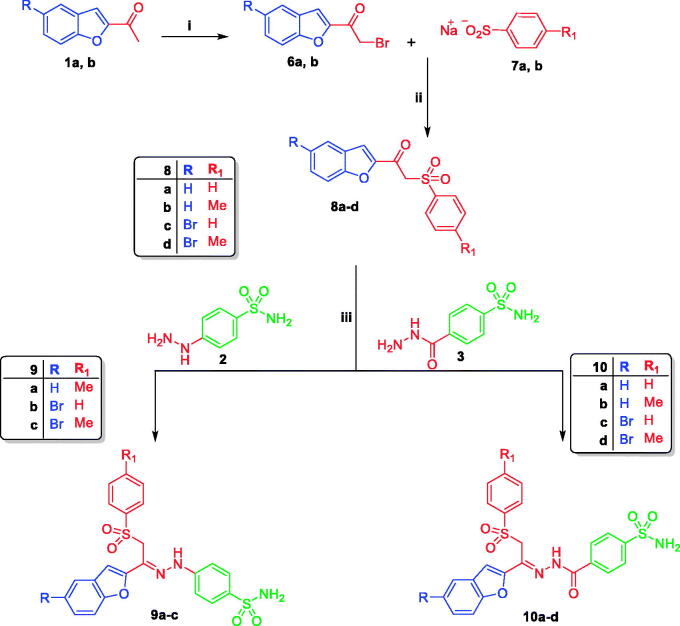
Reagent and conditions: (i) Br_2_/Acetic Acid, Stirring at r.t 4 h; (ii) Abs.Ethanol, reflux 4 h; (iii) Ethanol/Acetic acid, reflux 4 h.

The preparation of benzofuran-based sulphonamides **4a,b, 5a,b 9b–d**, and **10a–d** in this study is illustrated in Schemes 1 and 2. The synthesis was initiated by condensation of 2-acetylbenzofuran **1a** and 5-bromo-2-acetylbenzofuran **1b** with 4-hydrazinylbenzenesulfonamide **2** or 4-(hydrazinecarbonyl)benzenesulfonamide **3** in refluxing glacial acetic acid to furnish the target benzofuran-based sulphonamides **4a,b** and **5a,b** in 79–83% yield (Scheme 1).

In Scheme 2, 2-acetylbenzofurans **1a** and **1b** were brominated by the use of bromine in glacial acetic acid to afford 1-(benzofuran-2-yl)-2-bromoethan-1-ones **6a** and **6b**, respectively. Thereafter, the brominated intermediates **6a** and **6b** were refluxed with sodium benzene sulfinates **7a** and **7b** in ethanol to obtain key intermediates **8a** and **8b**. Consequently, these key intermediates were condensed with 4-hydrazinylbenzenesulfonamide **2** or 4-(hydrazinecarbonyl)benzenesulfonamide **3** in refluxing ethanol containing catalytic amount of glacial acetic acid to furnish the target benzofurans **9a–c** and **10a–d**, respectively (Scheme 2).

Postulated structure of the newly synthesised benzofuran-based sulphonamides **4a,b, 5a,b 9b–d**, and **10a–d** were in full agreement with their spectral and elemental analyses data.

### Biological evaluation

#### Carbonic anhydrase inhibition

The newly prepared benzofuran-based sulphonamides **4a,b, 5a,b 9b–d**, and **10a–d** were evaluated for their ability to inhibit the physiologically relevant hCA isoforms, hCA I, II (cytosolic), and hCA IX and XII (trans membrane, tumour associated isoforms) using acetazolamide (AAZ) as standard inhibitor by a stopped flow CO_2_ hydras assay . The inhibition data of the prepared benzensulfonamides and **AAZ** against the examined isoforms are summarised in Table 1.

**Table 1.** Inhibition data of human CA isoforms hCA I, II, IX and XII for the target sulphonamides (**4a,b, 5a,b, 9a–c,** and **10a–d**), using (AAZ) as a standard drug.


Compound	R	R_1_	*K*_I_ (nM)*			
			hCA I	hCA II	hCA IX	hCA XII
**4a**	H	–	162.8	12.3	33.3	26.9
**4b**	Br	–	92.7	73.5	48.4	38.8
**5a**	H	–	37.4	33.6	60.4	10.1
**5b**	Br	–	63.9	44.2	27.7	32.5
**9a**	H	CH_3_	1292	643.7	32.8	49.7
**9b**	Br	H	2159	888.2	44.6	33.4
**9c**	Br	CH_3_	2503	571.1	10.0	16.7
**10a**	H	H	4625	353.9	76.6	71.8
**10b**	H	CH_3_	3921	228.5	51.1	38.9
**10c**	Br	H	1822	438.8	85.4	66.8
**10d**	Br	CH_3_	827.6	727.1	97.5	27.5
**AAZ**	–	–	250.0	12.0	25.0	5.7

^*^ Mean from three different assays, by a stopped flow technique (errors were in the range of ±5–10% of the reported values).

- The ubiquitous cytosolic *h*CA I isoform was inhibited by the herein reported benzofuran-based sulphonamides in a variable degree. The benzofuran hydrazones **4a** and **4b** displayed moderate potency with inhibition constant (*K*_I_) values of 162.8 and 92.7 nM, respectively, whereas the benzofuran hydrazides **5a** and **5b** potently inhibited *h*CA I isoform with *K*_I_ values of 37.4 and 63.9 nM, respectively. Contrariwise, *h*CA I was weakly inhibited by both arylsulfonehydrazones **9a–c** and arylsulfonehydrazides **10a–d** with *K*_I_s ranging in the micromolar range, in detail, between 1.292 and 4.625 μM, except for the Br-substituted tolylsulfonehydrazide **10d** which displayed lower *K*_I_ value (827.6 nM).
- The *in vitro* kinetic data listed in Table 1 revealed that the physiologically dominant cytosolic hCA II isoform was inhibited in a similar fashion to *h*CA I inhibition profile. While, benzofuran hydrazones/hydrazides **4a,b/5a,b** effectively inhibited hCA II (*K*_I_s: 12.3–73.5 nM), arylsulfonehydrazones **9a–c** and arylsulfonehydrazides **10a–d** displayed weak inhibitory activity with *K*_I_s spanning in the high nanomolar range: 228.5–888.2 nM.
  In particular, benzofuran hydrazone **4a** (*K*_I_=12.3 nM) emerged as the most potent hCA II inhibitor in this study with comparable activity to the standard drug AAZ (*K*_I_=12 nM). It is noteworthy that grafting 5-Br substituent to the benzofuran moiety elicited a worsening of effectiveness toward hCA II, except for compound **9c** which exhibited a reduced *K*_I_ (571.1 nM) than its un-substituted analogue **9a** (*K*_I_=1643.7 nM).
- The tumour-associated hCA IX isoform was efficiently inhibited by the herein reported benzofuran-based sulphonamides (**4a**,**b**, **5a**,**b**, **9a–c** and **10a–d**) with *K*_I_ values in the nanomolar range, 10.0–97.5 nM, Table 1. Superiorly, sulphonamide **9c** displayed the best hCA IX inhibitory activity in this study (*K*_I_=10.0 nM) which is 2.5-times more potent than the standard drug AAZ (*K*_I_=25 nM). Also, compounds **4a**, **5b** and **9a** displayed potent inhibitory activity toward hCA IX isoform with *K*_I_ values equal 33.3, 27.7 and 32.8 nM, respectively.
  It is worth emphasising that replacement of the hydrazine linker in arylsulfonehydrazones **9a–c** (*K*_I_s = 32.8, 44.6 and 10.0 nM, respectively) with the hydrazide one furnished arylsulfonehydrazides **10a–d** with decreased hCA IX inhibitory activity (*K*_I_s = 76.6, 51.1, 85.4 and 97.5 nM, respectively).
- The data listed in Table 1 ascribed to the newly synthesised benzofuran-based sulphonamides (**4a**,**b**, **5a**,**b**, **9a–c** and **10a–d**) potent efficiency in inhibiting the transmembrane tumour-associated hCA XII isoform. The target sulphonamides possessed activity with *K*_I_ values spanning in the nanomolar range: 10.1–71.8 nM, Table 1. In particular, compound **5a** was the most potent hCA XII inhibitor in this study with *K*_I_ value of 10.1 nM. It is worth highlighting that the benzofuran hydrazides **5a** and **5b** showed an improved inhibitory profile (*K*_I_s = 10.1 and 32.5 nM, respectively) against hCA IIX in comparison to their benzofuran hydrazone analogues **4a** and **4b** (*K*_I_s = 26.9 and 38.8 nM, respectively).
- The calculated selectivity indexes (SIs) displayed in Table 2 undeniably ascribed to the arylsulfonehydrazones **9** excellent selectivity towards hCA IX and XII over hCA I (SIs ranges: 39.4–250.3 and 26.0–149.9, respectively) and over hCA II (SIs ranges: 19.6–57.1 and 13.0–34.2, respectively). Besides, arylsulfonehydrazides **10** displayed good selectivity towards hCA IX and XII over hCA I (SIs ranges: 8.5–76.7 and 27.3–100.8, respectively) and over hCA II (SIs ranges: 5.1–7.5 and 4.9–26.4, respectively). Conversely, both hydrazones **4** and hydrazides **5** failed to display a satisfied selectivity towards hCA IX and XII. The distinctive selectivity of series **9** and **10** could be attributed to incorporation of arylsulfone moieties which elicited a dramatic worsening of effectiveness against hCA I and II.

**Table 2.** Selectivity ratios for the inhibition of hCA IX and XII over hCA I and II for targeted compounds **4a, b, 5a, b, 9a–c** and **10a–d**.

Compound	I/IX	II/IX	I/XII	II/XII
**4a**	4.9	0.4	6.1	0.5
**4b**	1.9	1.5	2.4	1.9
**5a**	0.6	0.6	3.7	3.3
**5b**	2.3	1.6	2.0	1.4
**9a**	39.4	19.6	26.0	13.0
**9b**	48.4	19.9	64.6	26.6
**9c**	250.3	57.1	149.9	34.2
**10a**	60.4	4.6	64.4	4.9
**10b**	76.7	4.5	100.8	5.9
**10c**	21.3	5.1	27.3	6.6
**10d**	8.5	7.5	30.1	26.4
**AAZ**	10.0	0.5	43.9	2.2

### *In vitro* antitumor activity towards 60 cancer cell lines (NCI, USA)

The newly prepared benzofuran-based sulphonamides **4a,b, 5a,b 9a-c**, and **10a–d** were selected to be evaluated for their antitumor activity at the NCI-Developmental Therapeutic Programme (www.dtp.nci.nih.gov). They were evaluated at one dose primary anticancer screening assay, at 10 μM, toward the full panel of sixty cancer cell lines, in accordance with the protocol of the Drug Evaluation Branch, NCI, Bethesda^34^. The cell growth was evaluated using the sulforhodamine B (SRB) colorimetric assay^35–37^. The obtained data were reported as mean-graph of the percentage growth of the different treated tumour cells (Supplementary Materials).

**Figure 2.**
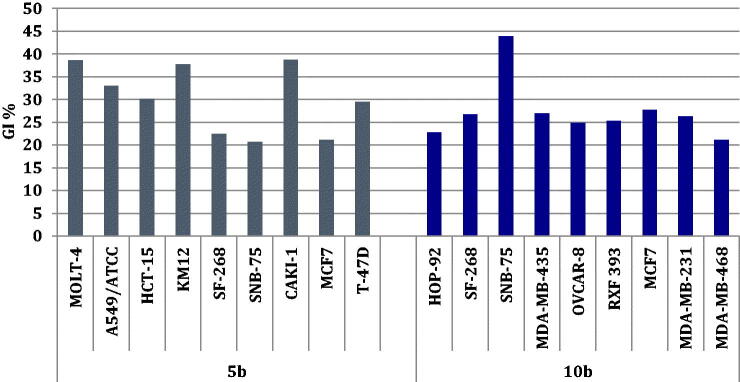
The most susceptible NCI cancer cell lines towards the impact of target sulphonamides **5b** and **10b** according to the GI%.

Investigation of the obtained results for this assay unveiled that only sulphonamides **5b** and **10b** possessed selective and moderate growth inhibitory activity toward certain cell lines, as displayed in Figure 2. Unfortunately, all the remaining target sulphonamides displayed non-significant anti-proliferative activity toward most NCI cancer cell lines.

## Conclusion

In summary, we successfully designed and synthesised novel benzofuran-based sulphonamides (**4a**,**b**, **5a**,**b**, **9a–c**, and **10a–d**) as a potent and selective CAIs. All the examined hCA isoforms were inhibited by the prepared benzofurans in variable degrees with the following *K*_I_s ranges: 37.4–4625 nM for hCA I, 12.3–888.2 nM for hCA II, 10.0–97.5 nM for hCA XI, and 10.1–71.8 nM for hCA XII. Regarding the selectivity of the target compounds, arylsulfonehydrazones **9** showed excellent selectivity towards hCA IX and XII over hCA I (SIs ranges: 39.4–250.3 and 26.0–149.9, respectively) and over hCA II (SIs ranges: 19.6–57.1 and 13.0–34.2, respectively). Besides, arylsulfonehydrazides **10** displayed good selectivity towards hCA IX and XII over hCA I (SIs: 8.5–76.7 and 27.3–100.8, respectively) and over hCA II (SIs: 5.1–7.5 and 4.9–26.4, respectively). The distinctive selectivity of series **9** and **10** could be attributed to incorporation of arylsulfone moieties which elicited a dramatic worsening of effectiveness against hCA I and II. The prepared benzofuran-based sulphonamides were further evaluated for their antitumor activity at the NCI-Developmental Therapeutic Programme. The obtained results unveiled that only sulphonamides **5b** and **10b** possessed selective and moderate growth inhibitory effect against certain cell lines, whereas, the remaining compounds displayed non-significant anti-proliferative activity toward most NCI cancer cell lines.

## Supplementary Material

Supplemental Material
