# Supplementary material for: Novel benzofuran-based sulphonamides as selective carbonic anhydrases IX and XII inhibitors: synthesis and in vitro biological evaluation
Source: J Enzyme Inhib Med Chem. 2019 Dec 6;35(1):298–305. doi: 10.1080/14756366.2019.1697250 (PMC6913630; doi:10.1080/14756366.2019.1697250)

4b-proton

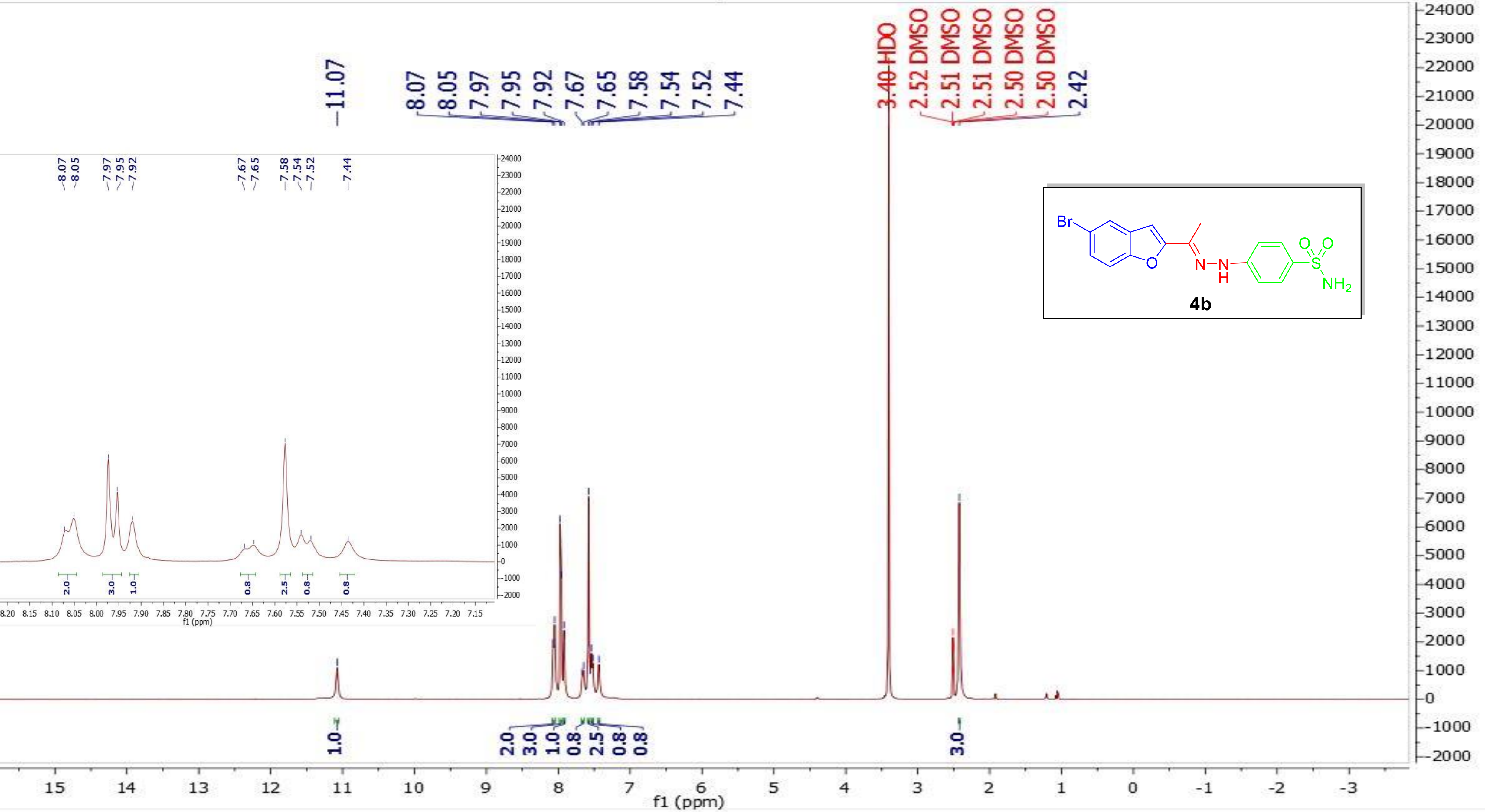

11.07
8.07
8.05
7.97
7.95
7.92
7.67
7.65
7.58
7.54
7.52
7.44
3.40 HDO
2.52 DMSO
2.51 DMSO
2.51 DMSO
2.50 DMSO
2.50 DMSO
2.42
Br
O
N
N
H
S
O
O
NH2
4b
1.0
2.0
3.0
1.0
0.8
2.5
0.8
0.8
3.0
f1 (ppm)

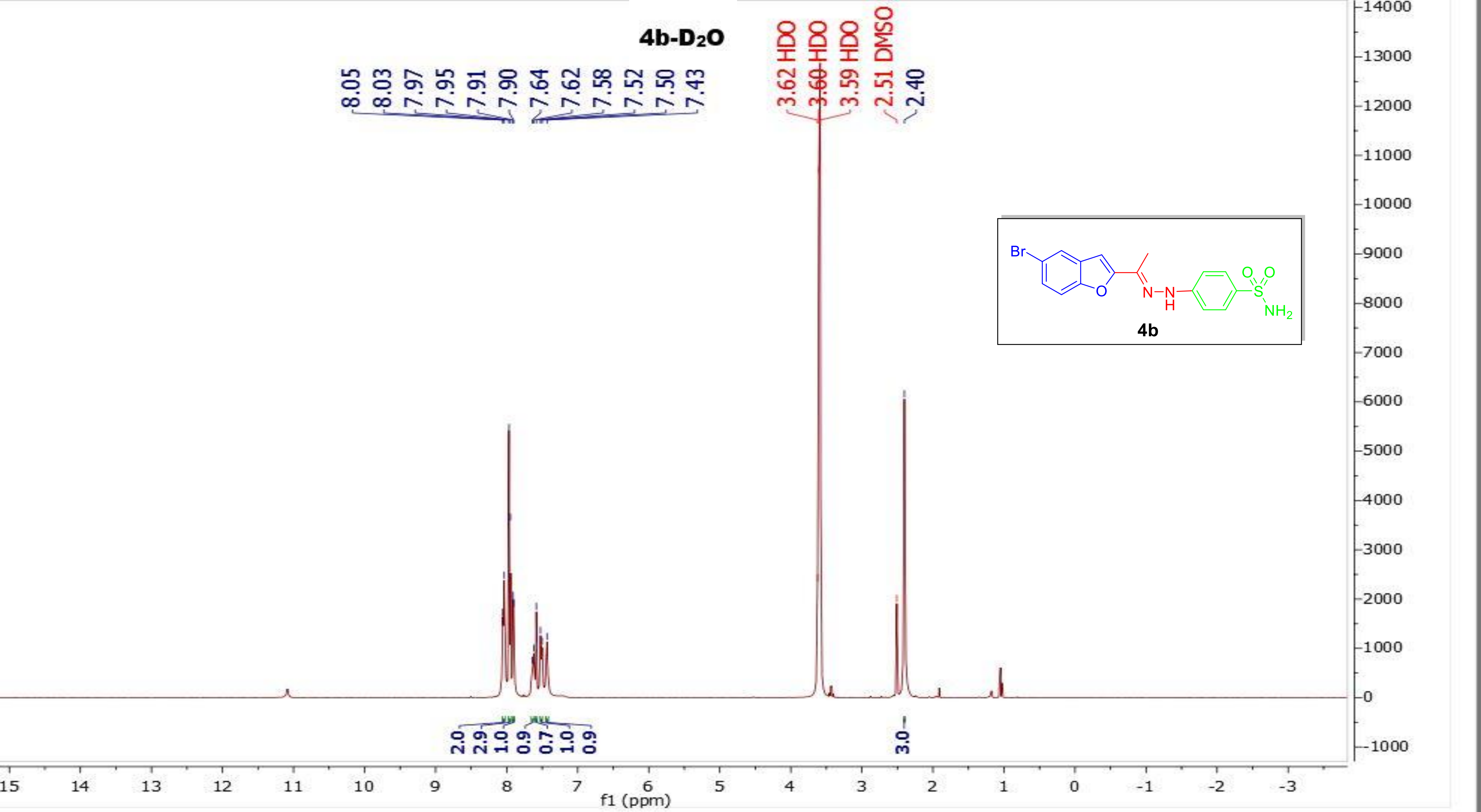
4b-D2O
8.05
8.03
7.97
7.95
7.91
7.90
7.64
7.62
7.58
7.52
7.50
7.43
3.62 HDO
3.60 HDO
3.59 HDO
2.51 DMSO
2.40
Br
O
N
N
H
O
O
S
NH2
4b
2.0
2.9
1.0
0.9
0.7
1.0
0.9
3.0
f1 (ppm)

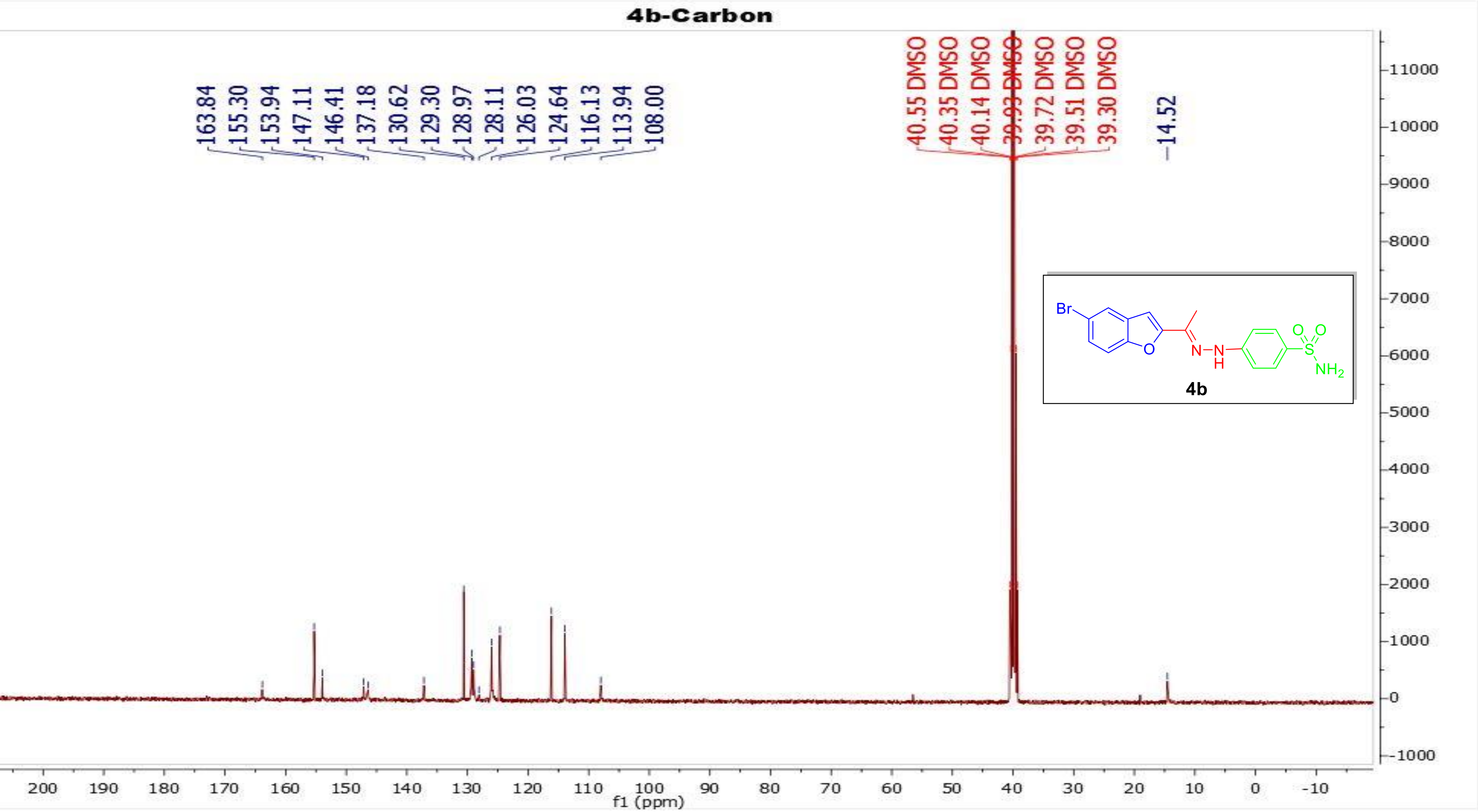
4b-Carbon
163.84
155.30
153.94
147.11
146.41
137.18
130.62
129.30
128.97
128.11
126.03
124.64
116.13
113.94
108.00
40.55 DMSO
40.35 DMSO
40.14 DMSO
39.93 DMSO
39.72 DMSO
39.51 DMSO
39.30 DMSO
14.52
Br
O
N
N
H
O
O
S
NH2
4b
f1 (ppm)

5a-proton

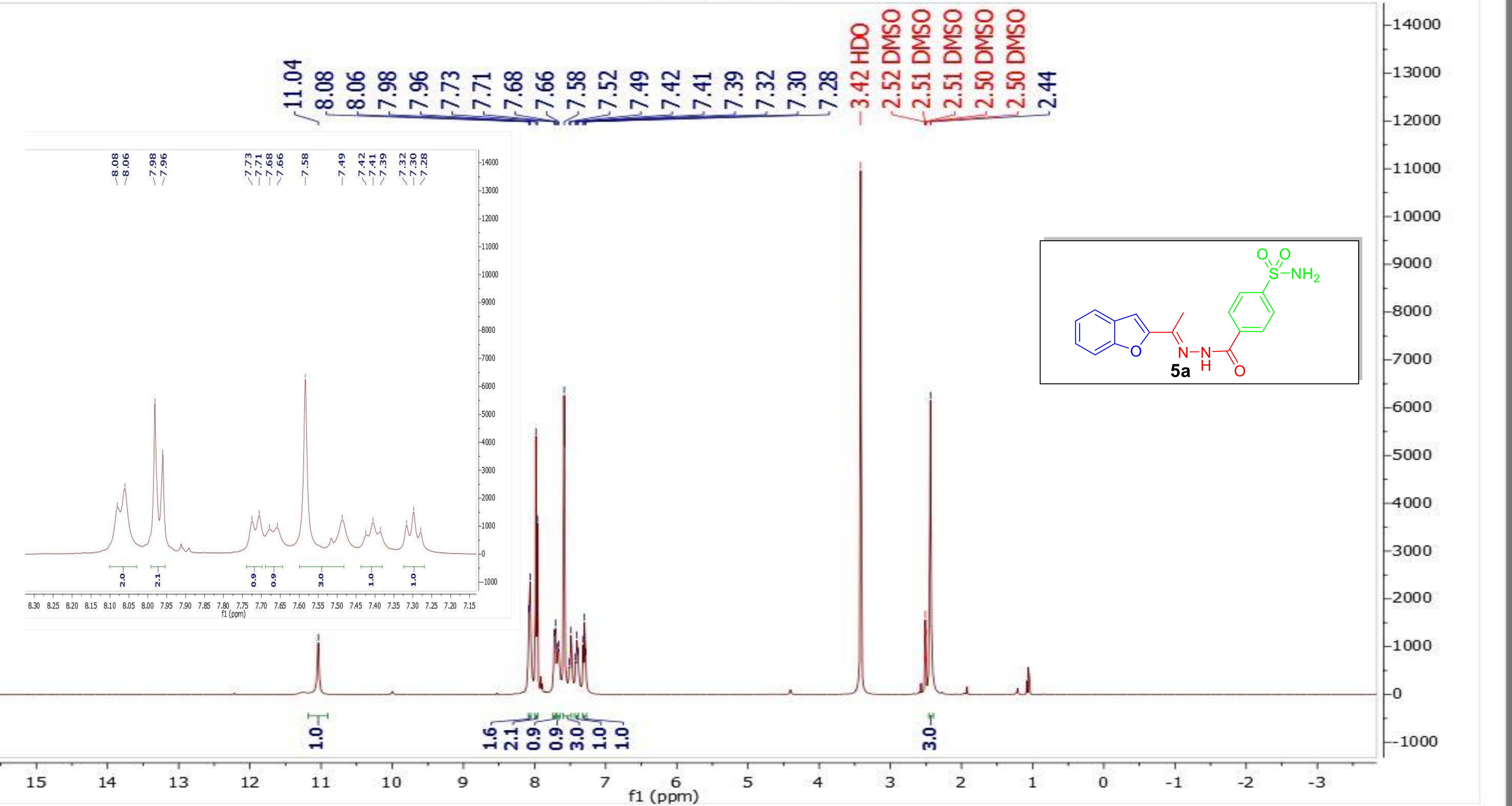
11.04
8.08
8.06
7.98
7.96
7.73
7.71
7.68
7.66
7.58
7.52
7.49
7.42
7.41
7.39
7.32
7.30
7.28
3.42 HDO
2.52 DMSO
2.51 DMSO
2.51 DMSO
2.50 DMSO
2.50 DMSO
2.44
1.0
1.6
2.1
0.9
0.9
3.0
1.0
1.0
3.0
f1 (ppm)
5a
O
N
H
NH2
S

5a-D2O

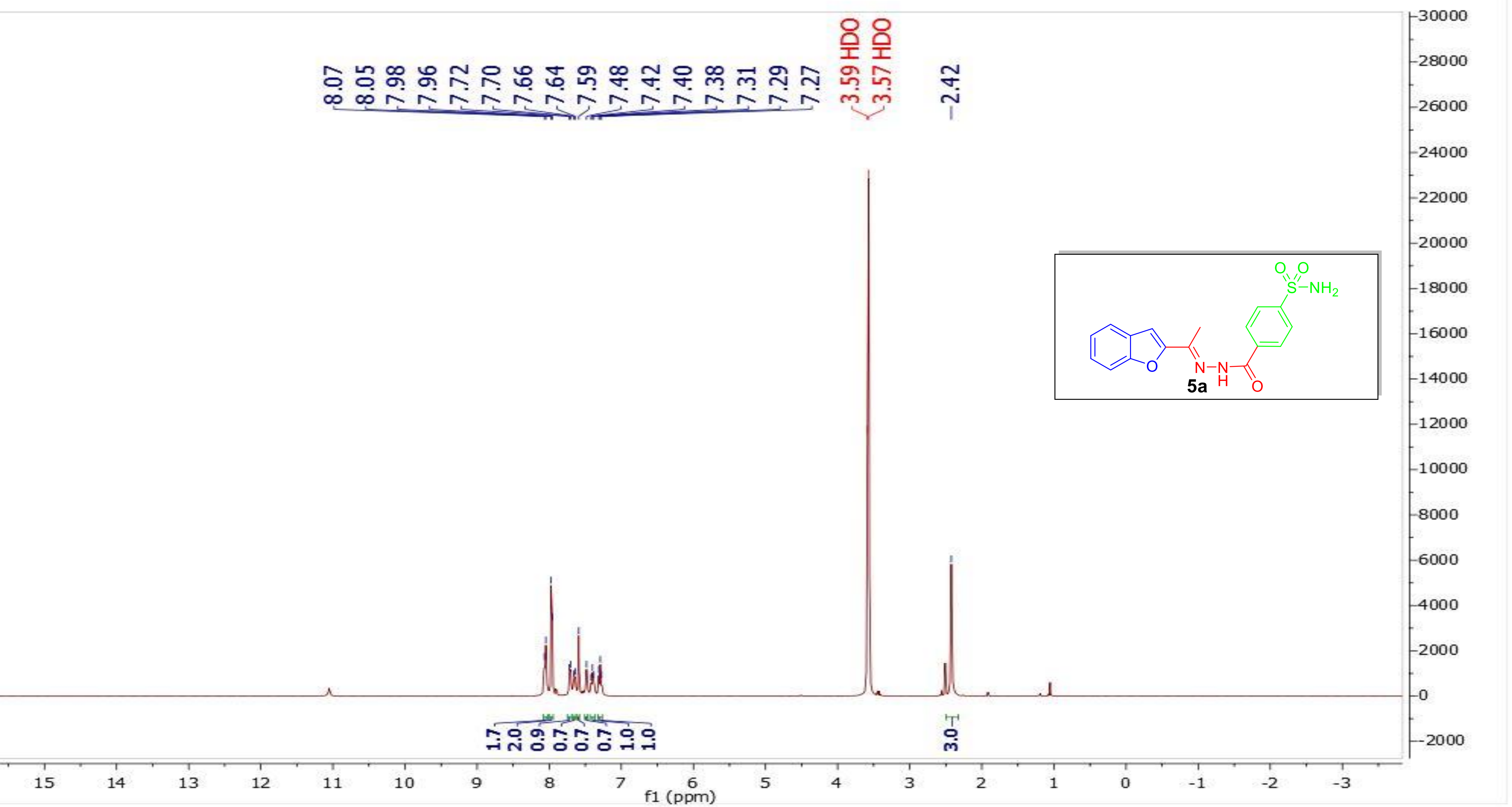
8.07
8.05
7.98
7.96
7.72
7.70
7.66
7.64
7.59
7.48
7.42
7.40
7.38
7.31
7.29
7.27
3.59 HDO
3.57 HDO
2.42
1.7
2.0
0.9
0.7
0.7
0.7
0.7
1.0
1.0
3.0
5a
f1 (ppm)

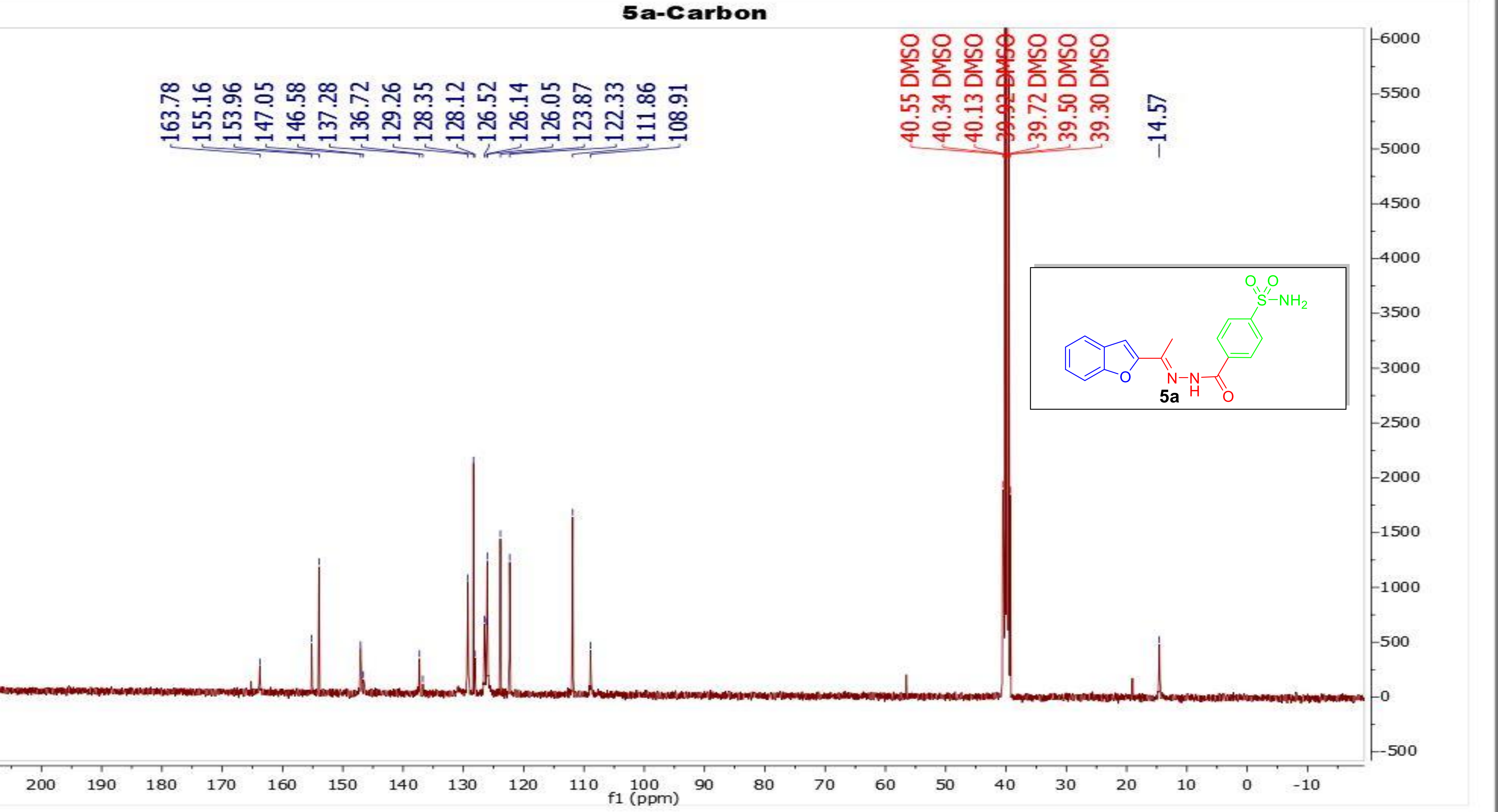
5a-Carbon
163.78
155.16
153.96
147.05
146.58
137.28
136.72
129.26
128.35
128.12
126.52
126.14
126.05
123.87
122.33
111.86
108.91
40.55 DMSO
40.34 DMSO
40.13 DMSO
39.92 DMSO
39.72 DMSO
39.50 DMSO
39.30 DMSO
14.57
5a
f1 (ppm)

5b-proton

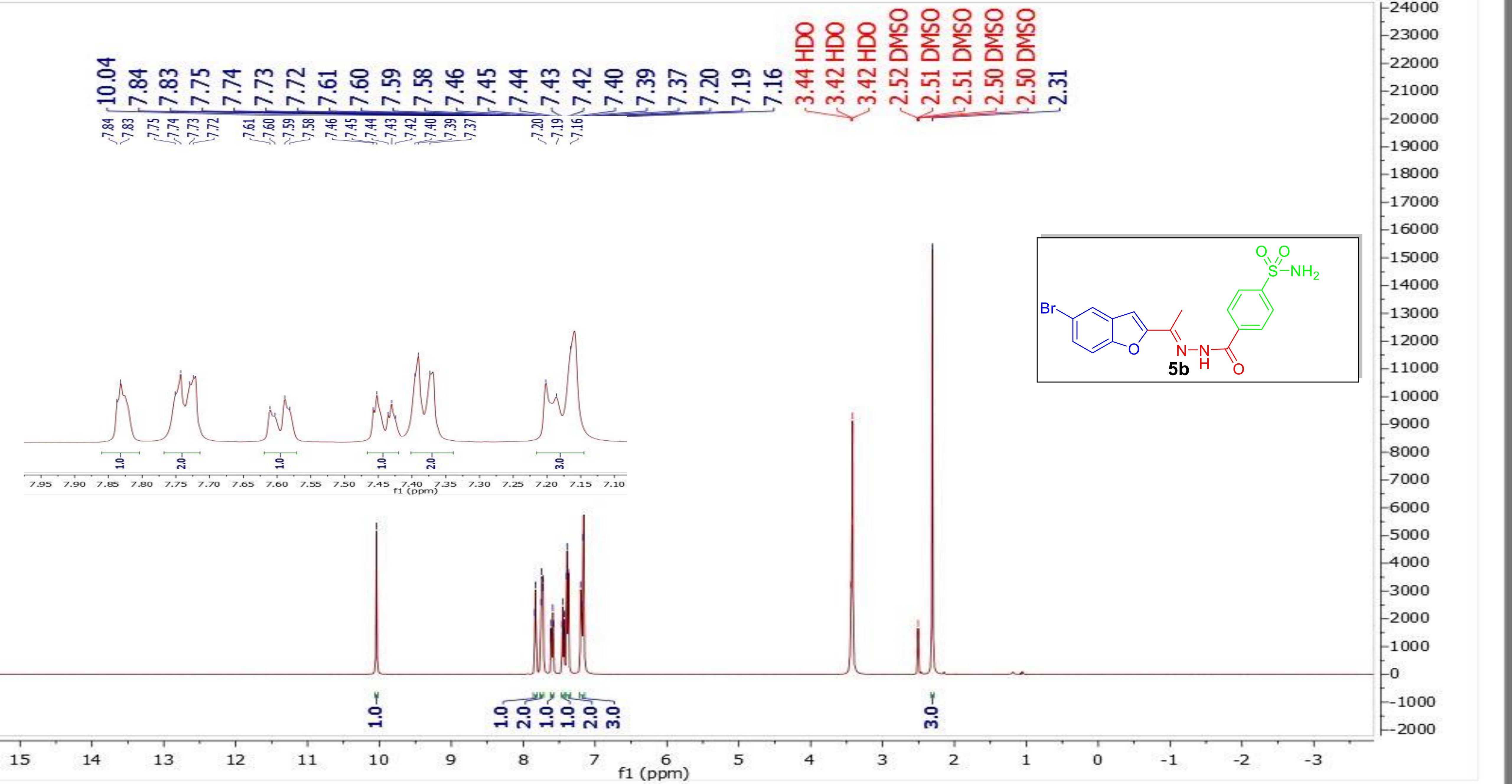
10.04
7.84
7.83
7.75
7.74
7.73
7.72
7.61
7.60
7.59
7.58
7.46
7.45
7.44
7.43
7.42
7.40
7.39
7.37
7.20
7.19
7.16
3.44 HDO
3.42 HDO
3.42 HDO
2.52 DMSO
2.51 DMSO
2.51 DMSO
2.50 DMSO
2.50 DMSO
2.31
Br
O
O
S
NH_2
O
N
N
H
O
5b
f1 (ppm)

5b-D$_2$O

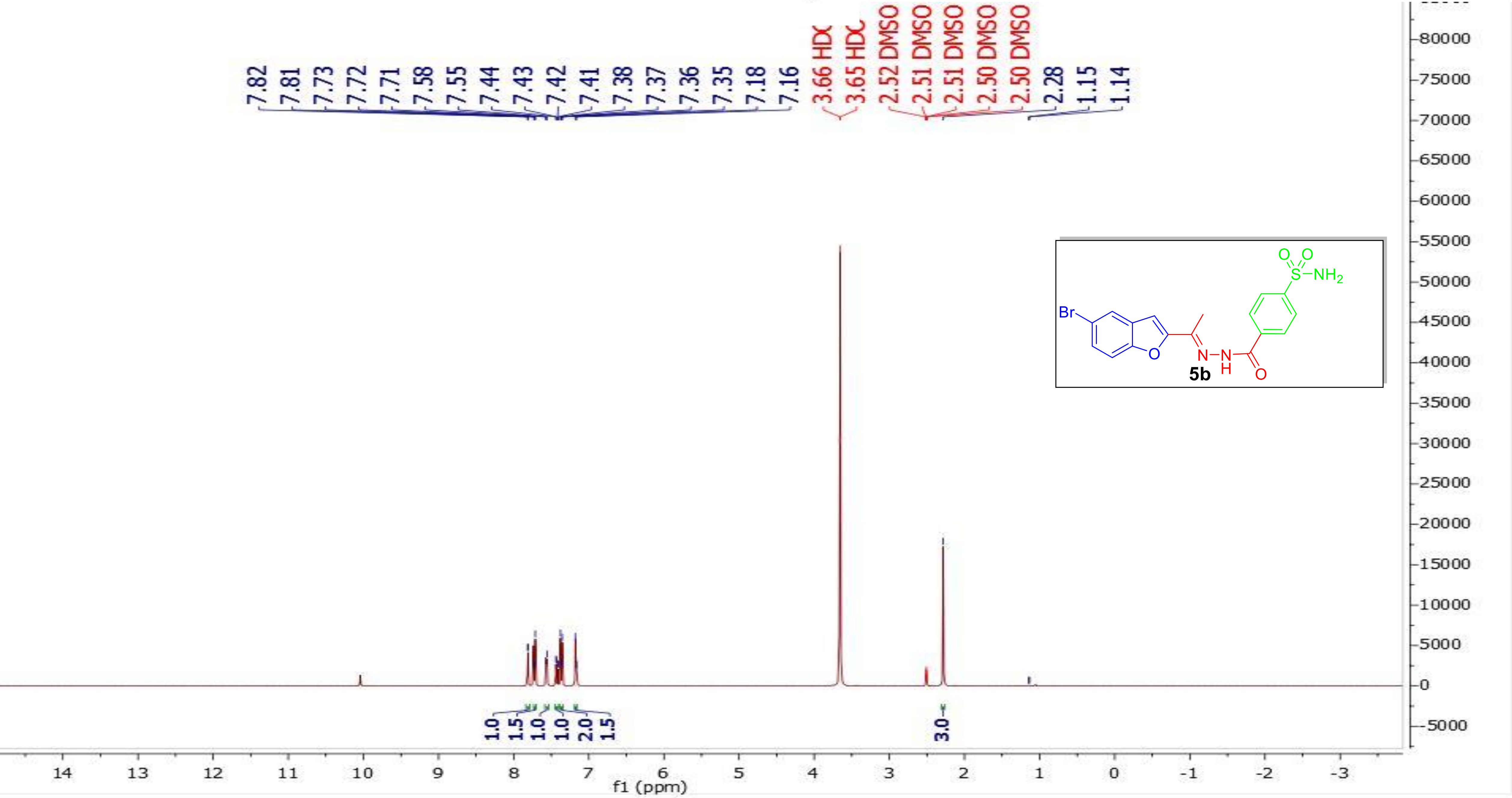
7.82 7.81 7.73 7.72 7.71 7.58 7.55 7.44 7.43 7.42 7.41 7.38 7.37 7.36 7.35 7.18 7.16
3.66 HDO 3.65 HDO
2.52 DMSO 2.51 DMSO 2.51 DMSO 2.50 DMSO 2.50 DMSO
2.28 1.15 1.14
1.0 1.5 1.0 1.0 2.0 1.5
3.0
Br
O
N
N
H
O
O
O
S
NH2
5b
f1 (ppm)
14 13 12 11 10 9 8 7 6 5 4 3 2 1 0 -1 -2 -3
80000 75000 70000 65000 60000 55000 50000 45000 40000 35000 30000 25000 20000 15000 10000 5000 0 -5000

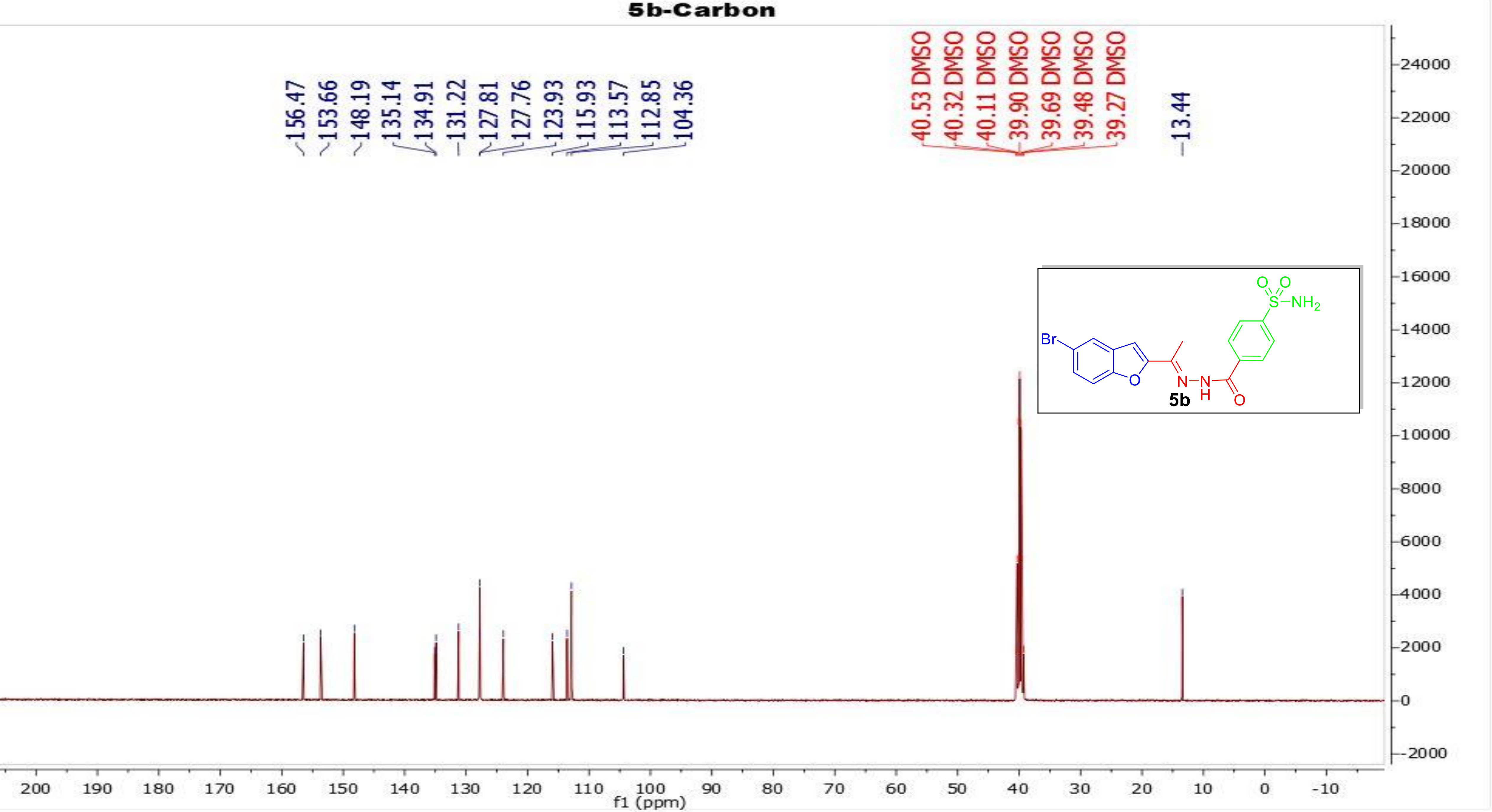

5b-Carbon
156.47
153.66
148.19
135.14
134.91
131.22
127.81
127.76
123.93
115.93
113.57
112.85
104.36
40.53 DMSO
40.32 DMSO
40.11 DMSO
39.90 DMSO
39.69 DMSO
39.48 DMSO
39.27 DMSO
13.44
Br
O
N
N
H
O
S
O
O
NH2
5b
f1 (ppm)

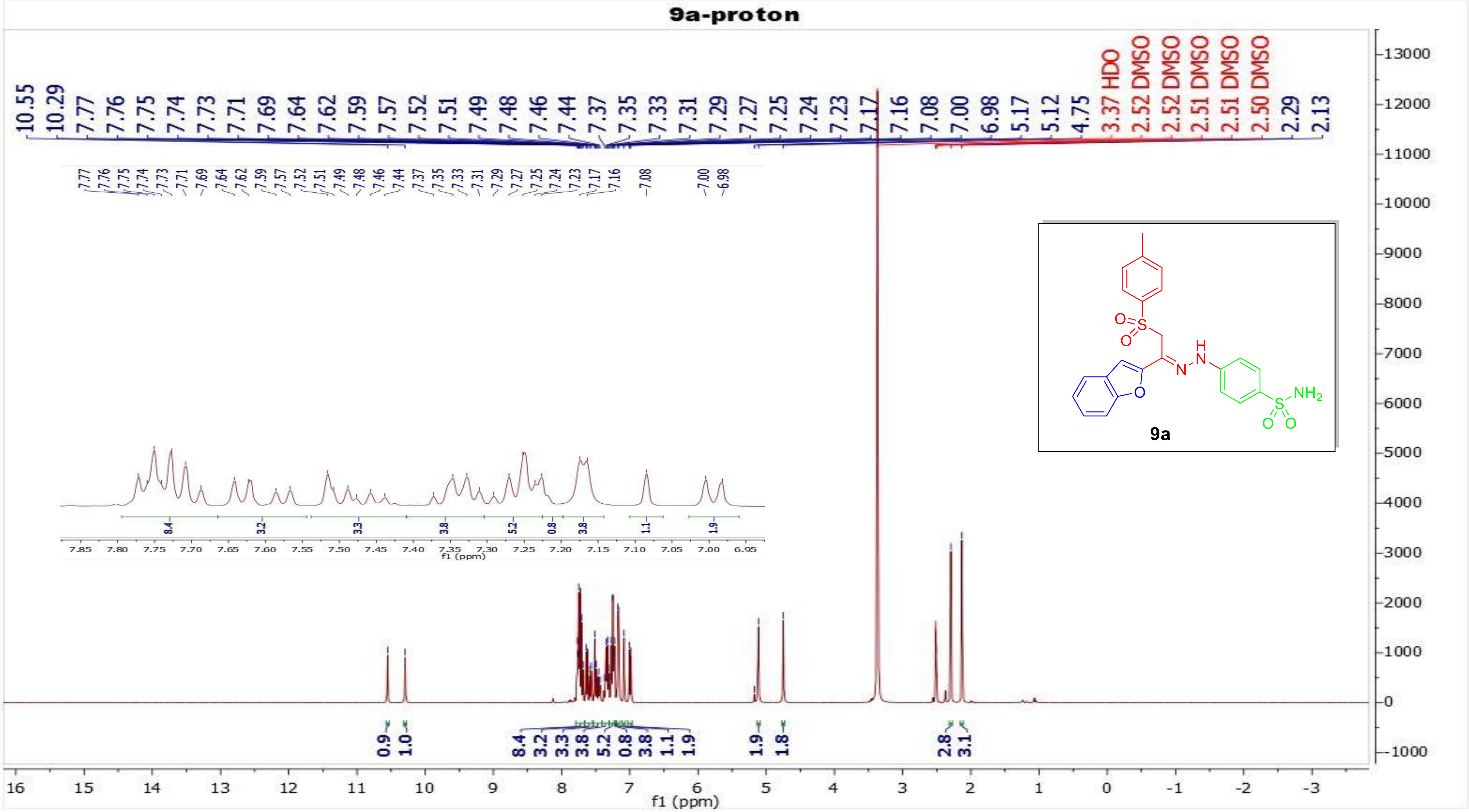
9a-proton
10.55
10.29
7.77
7.76
7.75
7.74
7.73
7.71
7.69
7.64
7.62
7.59
7.57
7.52
7.51
7.49
7.48
7.46
7.44
7.37
7.35
7.33
7.31
7.29
7.27
7.25
7.24
7.23
7.17
7.16
7.08
7.00
6.98
5.17
5.12
4.75
3.37 HDO
2.52 DMSO
2.52 DMSO
2.51 DMSO
2.51 DMSO
2.50 DMSO
2.29
2.13
9a
f1 (ppm)

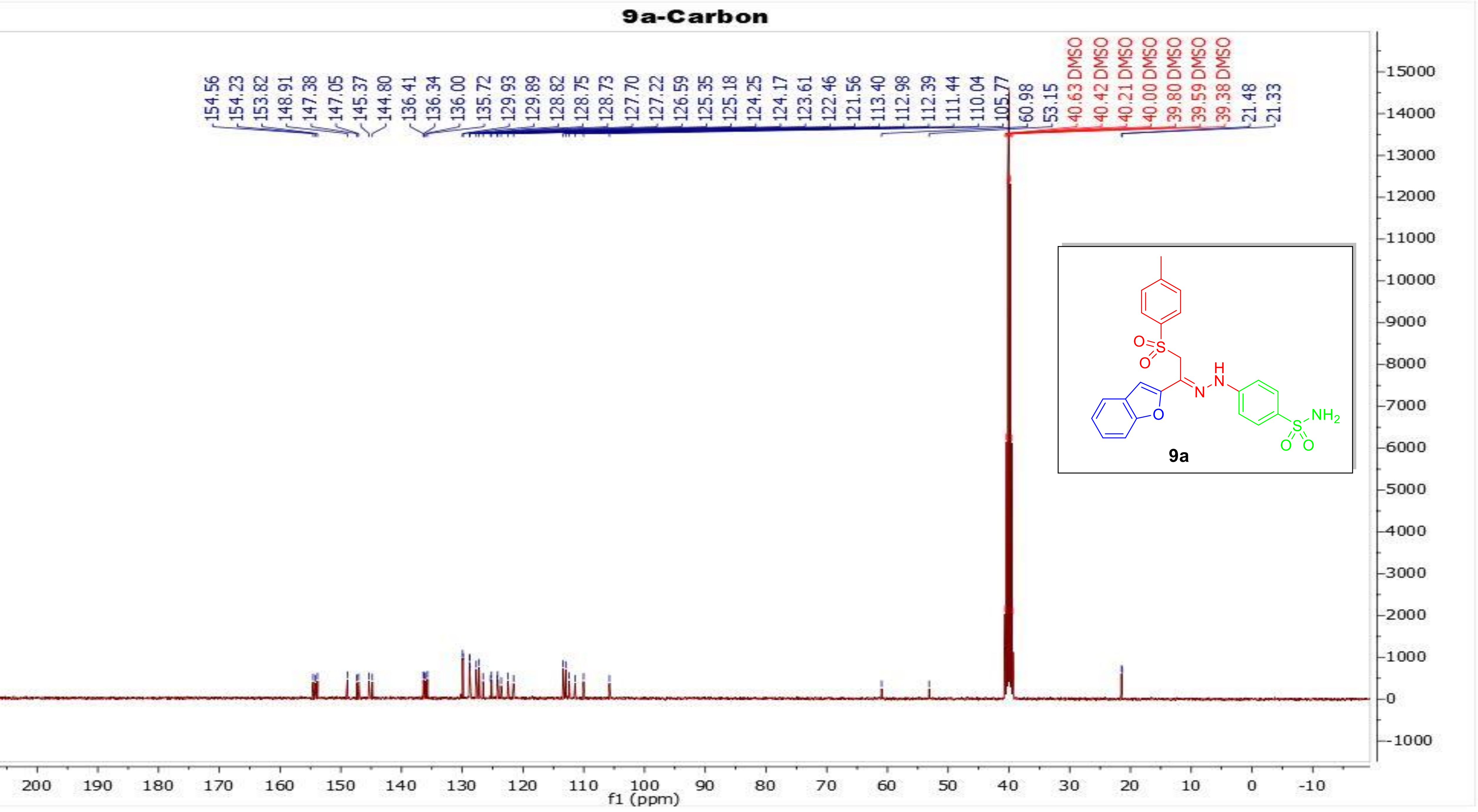
9a-Carbon
154.56
154.23
153.82
148.91
147.38
147.05
145.37
144.80
136.41
136.34
136.00
135.72
129.93
129.89
128.82
128.75
128.73
127.70
127.22
126.59
125.35
125.18
124.25
124.17
123.61
122.46
121.56
113.40
112.98
112.39
111.44
110.04
105.77
60.98
53.15
40.63 DMSO
40.42 DMSO
40.21 DMSO
40.00 DMSO
39.80 DMSO
39.59 DMSO
39.38 DMSO
21.48
21.33
9a
f1 (ppm)

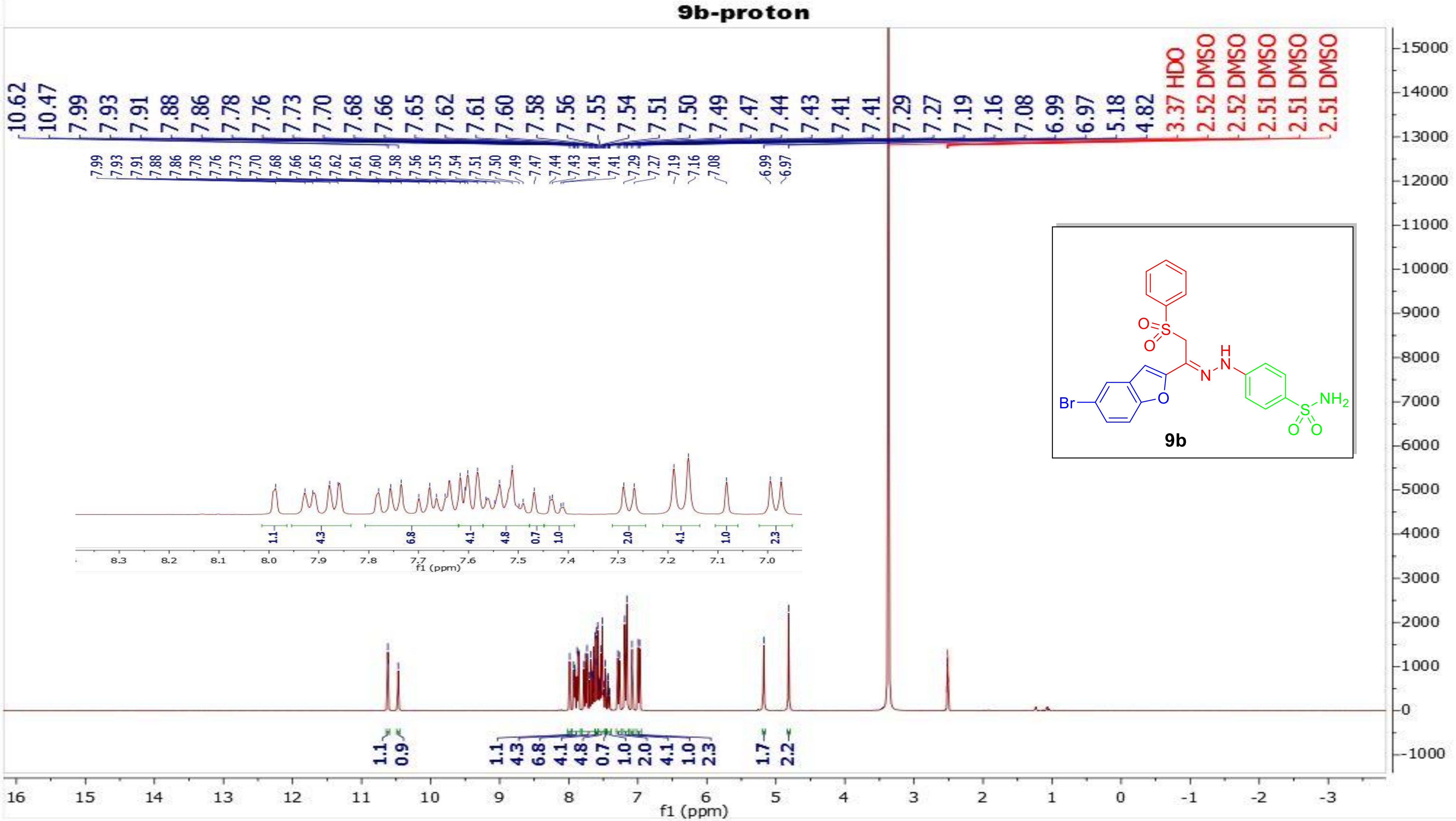
9b-proton
10.62
10.47
7.99
7.93
7.91
7.88
7.86
7.78
7.76
7.73
7.70
7.68
7.66
7.65
7.62
7.61
7.60
7.58
7.56
7.55
7.54
7.51
7.50
7.49
7.47
7.44
7.43
7.41
7.41
7.29
7.27
7.19
7.16
7.08
6.99
6.97
5.18
4.82
3.37 HDO
2.52 DMSO
2.52 DMSO
2.51 DMSO
2.51 DMSO
2.51 DMSO
Br
O
O
S
O
H
N
N
NH2
S
O
O
9b
f1 (ppm)

9b-Carbon

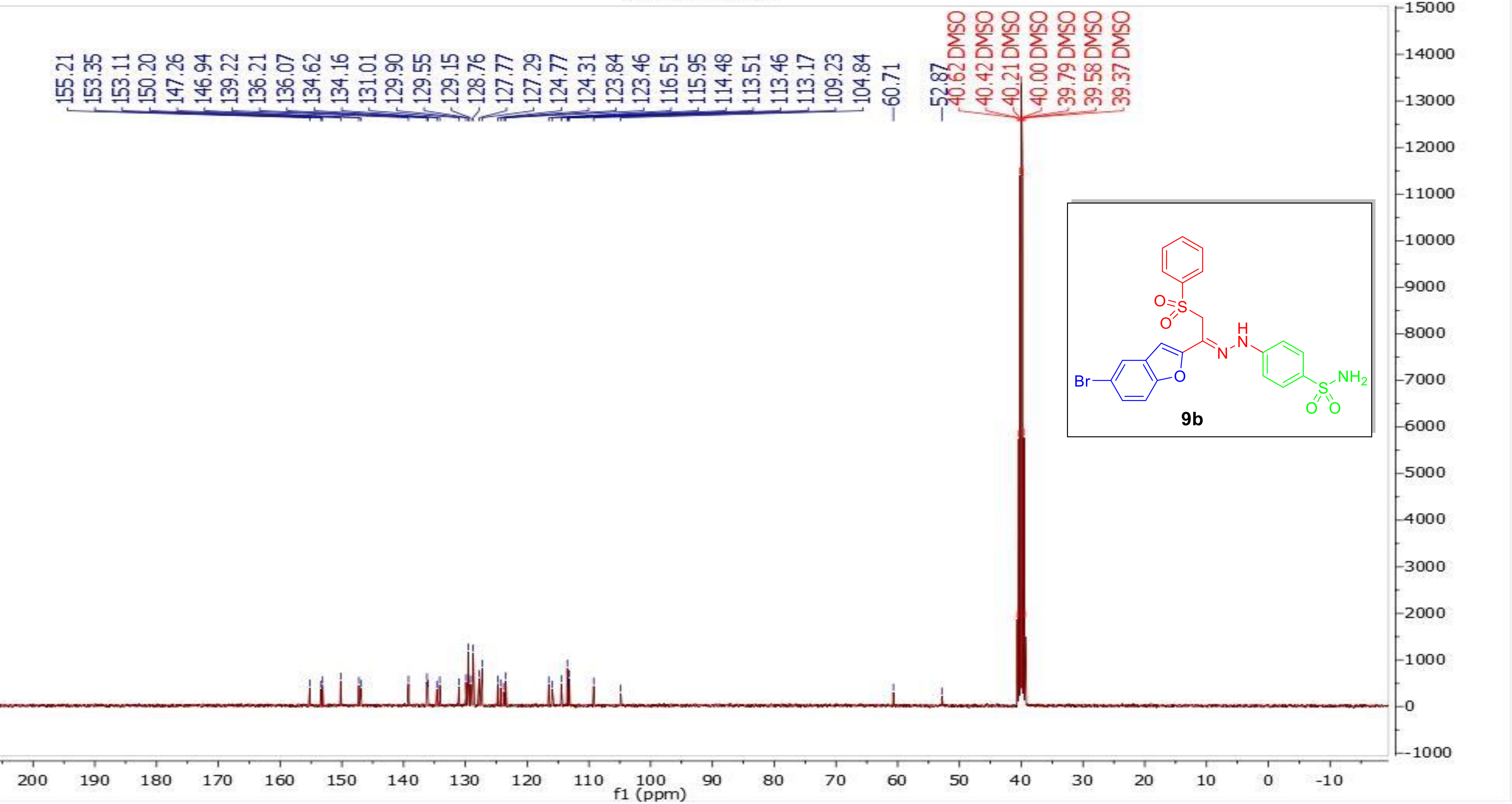
155.21
153.35
153.11
150.20
147.26
146.94
139.22
136.21
136.07
134.62
134.16
131.01
129.90
129.55
129.15
128.76
127.77
127.29
124.77
124.31
123.84
123.46
116.51
115.95
114.48
113.51
113.46
113.17
109.23
104.84
60.71
52.87
40.62 DMSO
40.42 DMSO
40.21 DMSO
40.00 DMSO
39.79 DMSO
39.58 DMSO
39.37 DMSO
Br
O
N
H
N
O
S
O
NH2
S
O
O
9b
f1 (ppm)
200 190 180 170 160 150 140 130 120 110 100 90 80 70 60 50 40 30 20 10 0 -10
15000 14000 13000 12000 11000 10000 9000 8000 7000 6000 5000 4000 3000 2000 1000 0 -1000

9c-proton

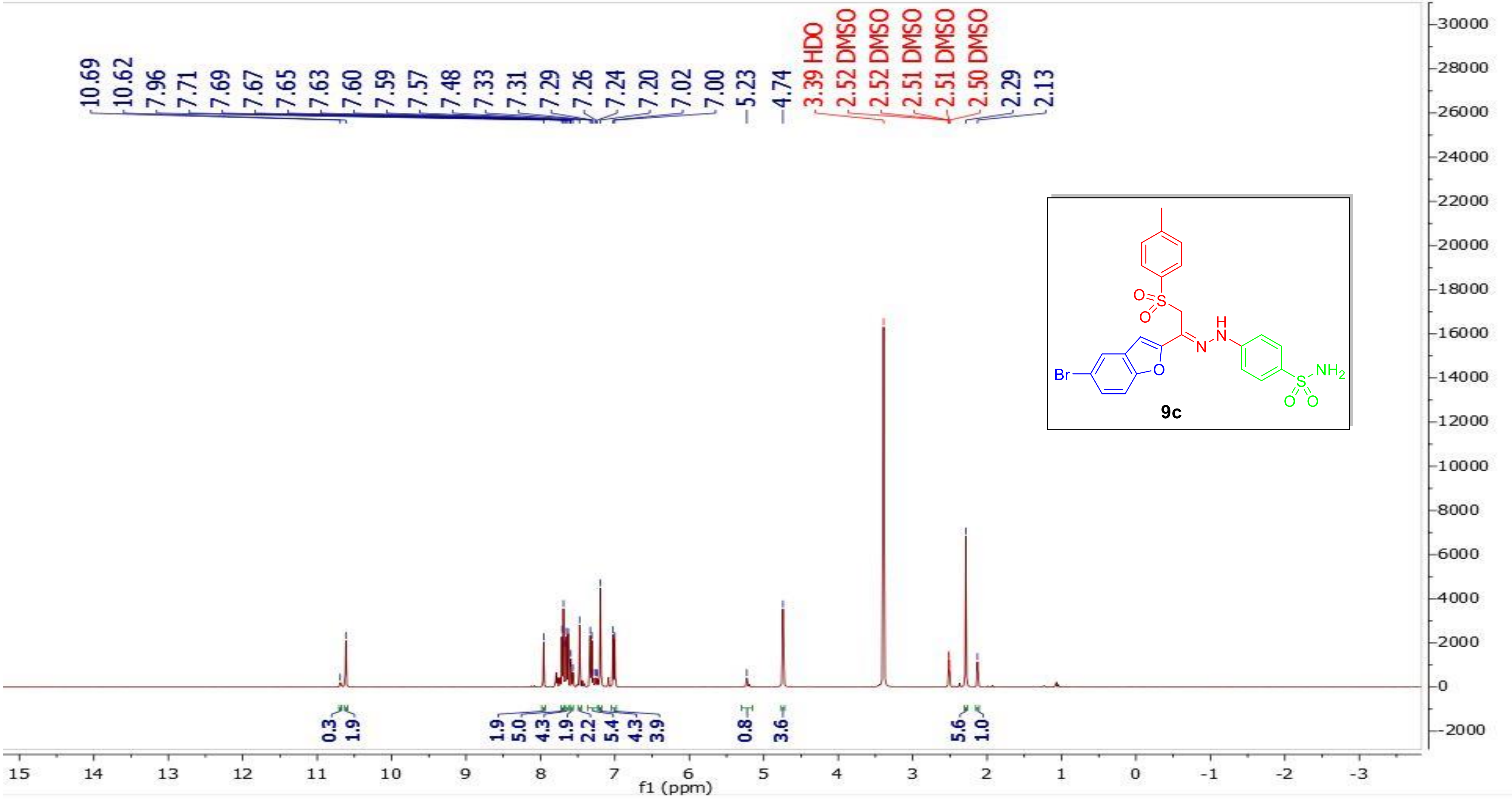

10.69
10.62
7.96
7.71
7.69
7.67
7.65
7.63
7.60
7.59
7.57
7.48
7.33
7.31
7.29
7.26
7.24
7.20
7.02
7.00
5.23
4.74
3.39 HDO
2.52 DMSO
2.52 DMSO
2.51 DMSO
2.51 DMSO
2.50 DMSO
2.29
2.13
Br
O
O
S
O
H
N
N
NH2
S
O
O
9c
0.3
1.9
1.9
5.0
4.3
1.9
2.2
5.4
4.3
3.9
0.8
3.6
5.6
1.0
15
14
13
12
11
10
9
8
7
6
5
4
3
2
1
0
-1
-2
-3
f1 (ppm)
30000
28000
26000
24000
22000
20000
18000
16000
14000
12000
10000
8000
6000
4000
2000
0
-2000

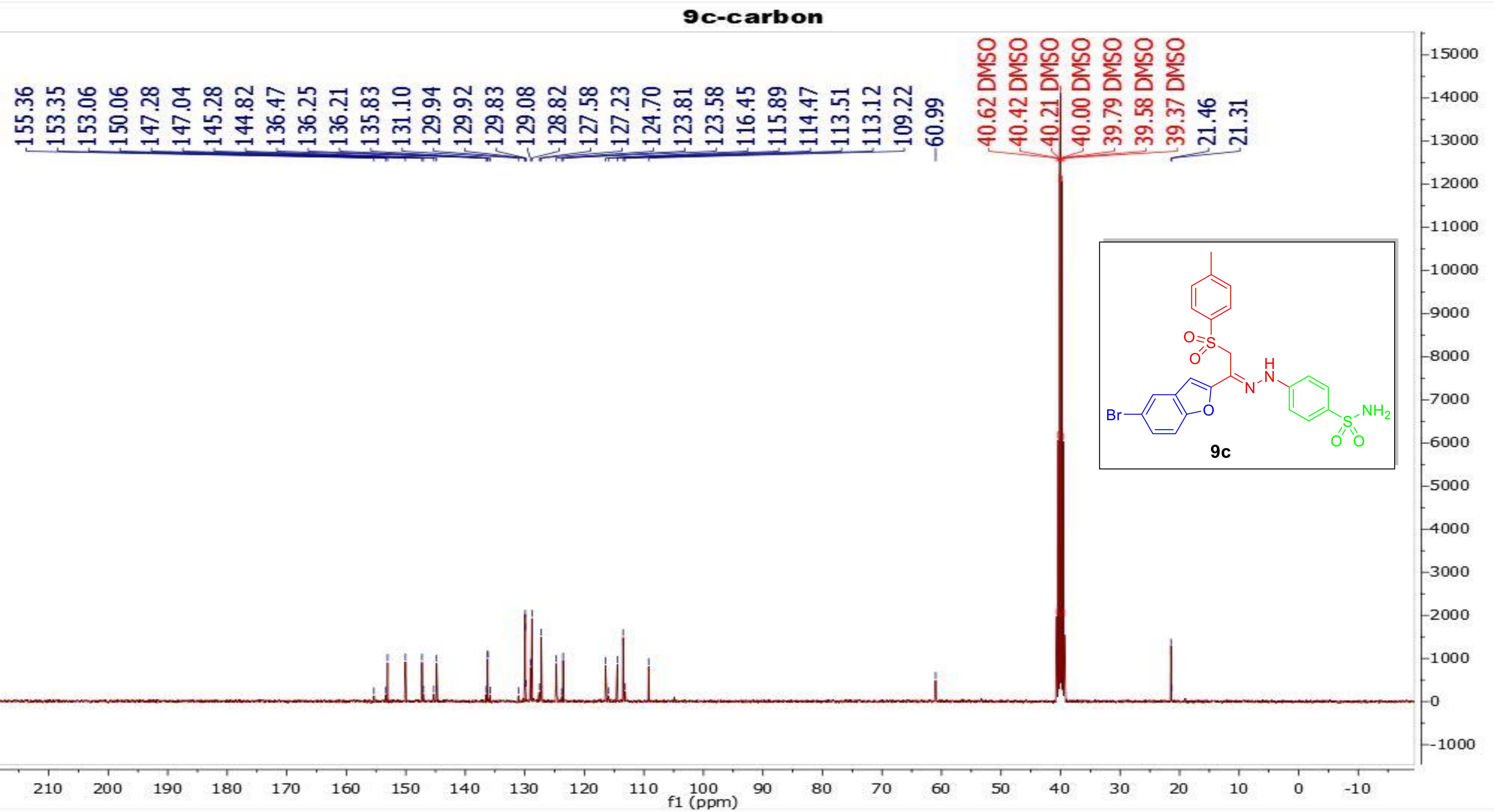
9c-carbon
155.36
153.35
153.06
150.06
147.28
147.04
145.28
144.82
136.47
136.25
136.21
135.83
131.10
129.94
129.92
129.83
129.08
128.82
127.58
127.23
124.70
123.81
123.58
116.45
115.89
114.47
113.51
113.12
109.22
60.99
40.62 DMSO
40.42 DMSO
40.21 DMSO
40.00 DMSO
39.79 DMSO
39.58 DMSO
39.37 DMSO
21.46
21.31
Br
O
O
S
O
H
N
N
NH2
S
O
O
9c
f1 (ppm)

10a-proton

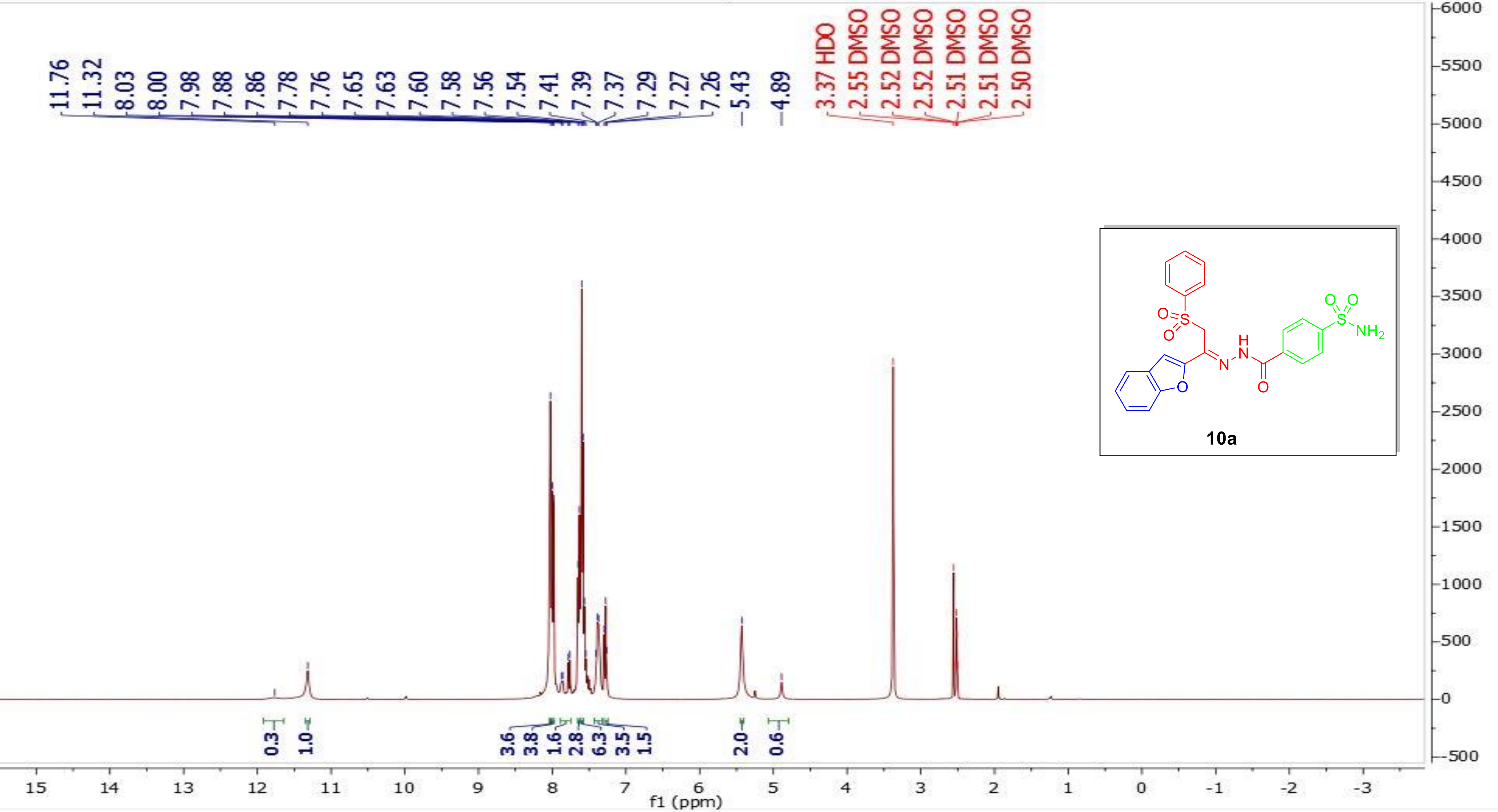

11.76
11.32
8.03
8.00
7.98
7.88
7.86
7.78
7.76
7.65
7.63
7.60
7.58
7.56
7.54
7.41
7.39
7.37
7.29
7.27
7.26
5.43
4.89
3.37 HDO
2.55 DMSO
2.52 DMSO
2.52 DMSO
2.51 DMSO
2.51 DMSO
2.50 DMSO
0.3
1.0
3.6
3.8
1.6
2.8
6.3
3.5
1.5
2.0
0.6
10a
O
S
NH2
H
N
f1 (ppm)

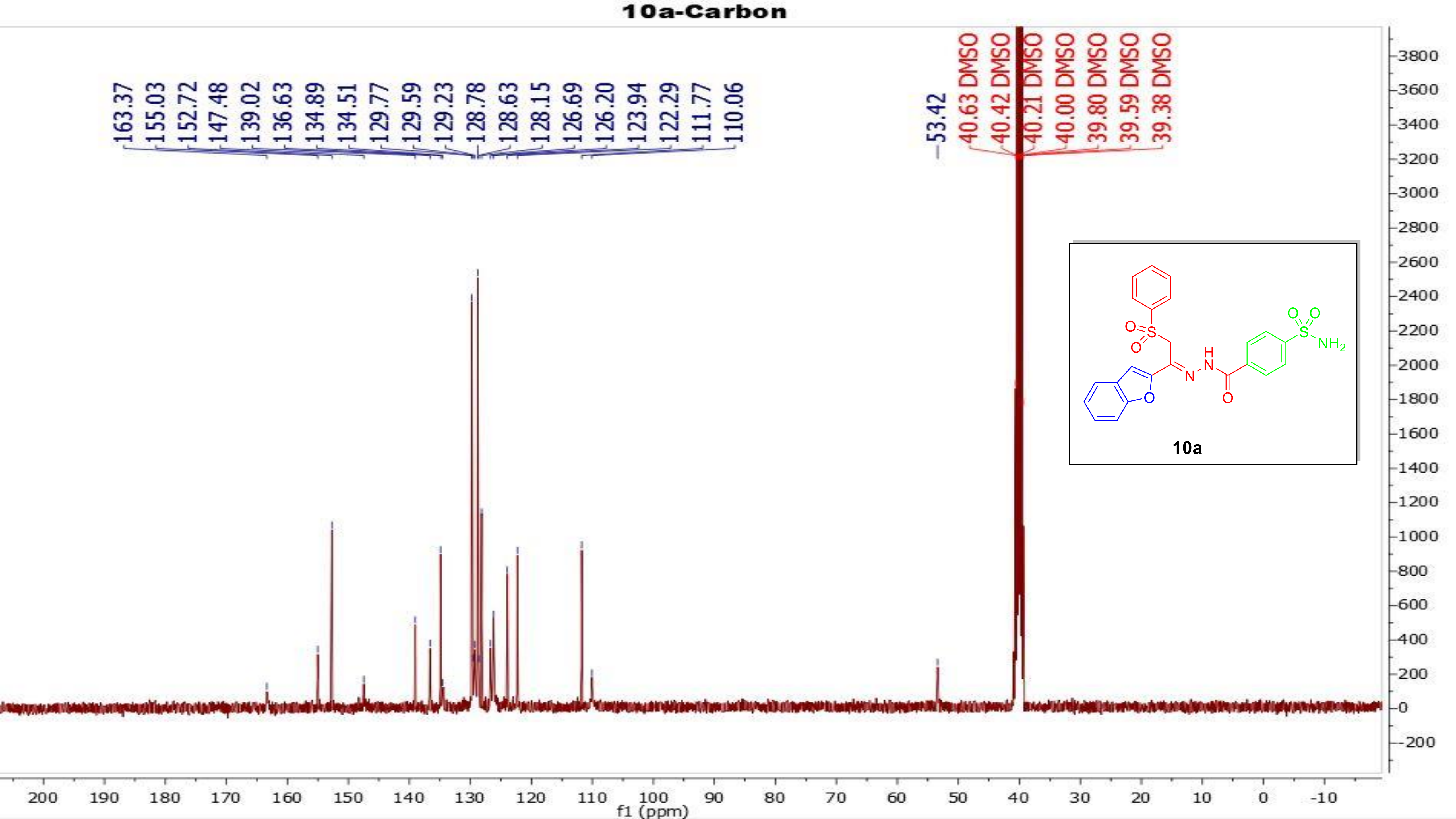
10a-Carbon
163.37
155.03
152.72
147.48
139.02
136.63
134.89
134.51
129.77
129.59
129.23
128.78
128.63
128.15
126.69
126.20
123.94
122.29
111.77
110.06
53.42
40.63 DMSO
40.42 DMSO
40.21 DMSO
40.00 DMSO
39.80 DMSO
39.59 DMSO
39.38 DMSO
10a
f1 (ppm)

10b-proton

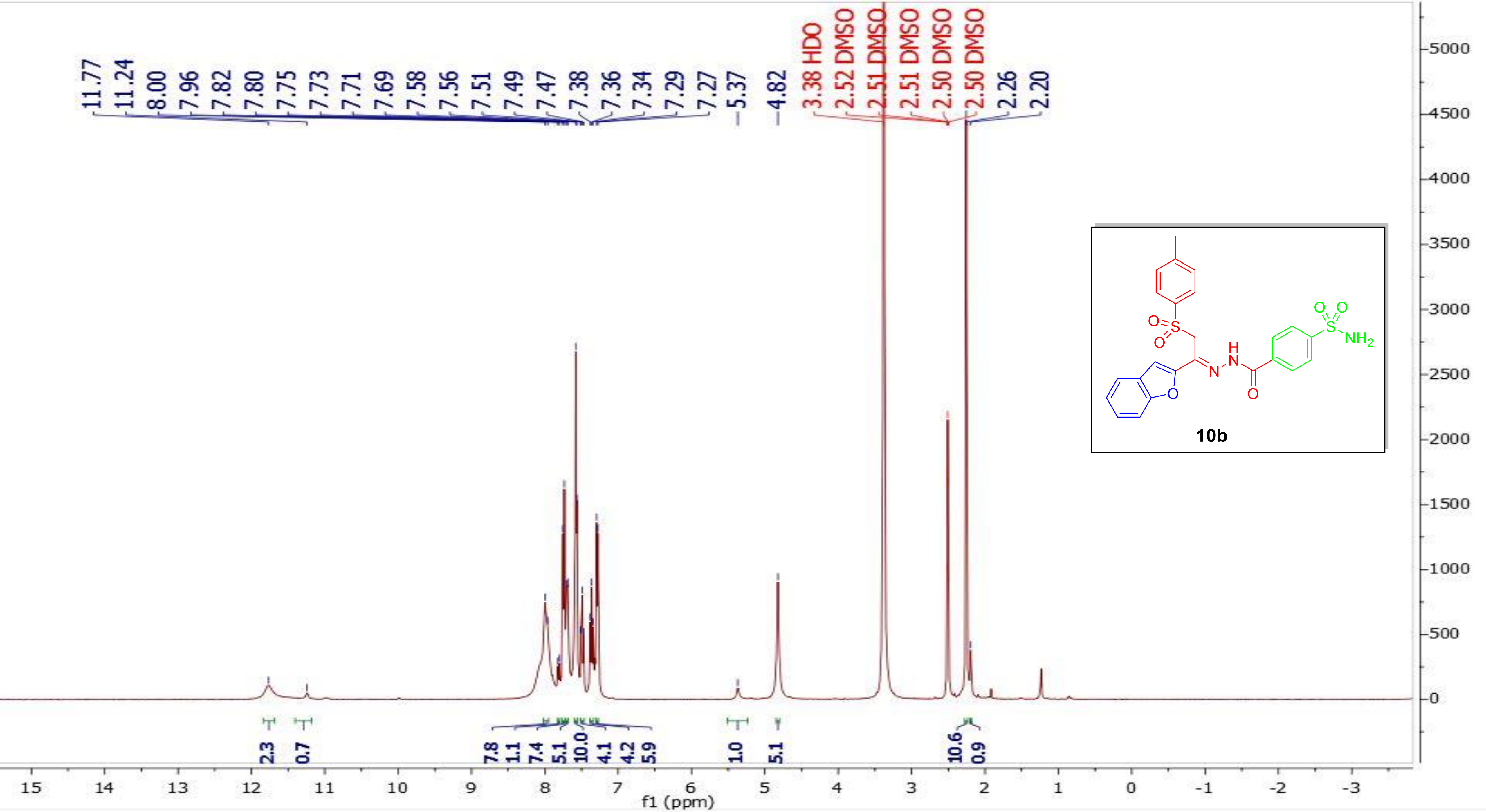
11.77
11.24
8.00
7.96
7.82
7.80
7.75
7.73
7.71
7.69
7.58
7.56
7.51
7.49
7.47
7.38
7.36
7.34
7.29
7.27
5.37
4.82
3.38 HDO
2.52 DMSO
2.51 DMSO
2.51 DMSO
2.50 DMSO
2.50 DMSO
2.26
2.20
2.3
0.7
7.8
1.1
7.4
5.1
10.0
4.1
4.2
5.9
1.0
5.1
10.6
0.9
10b
f1 (ppm)

10b-D₂O

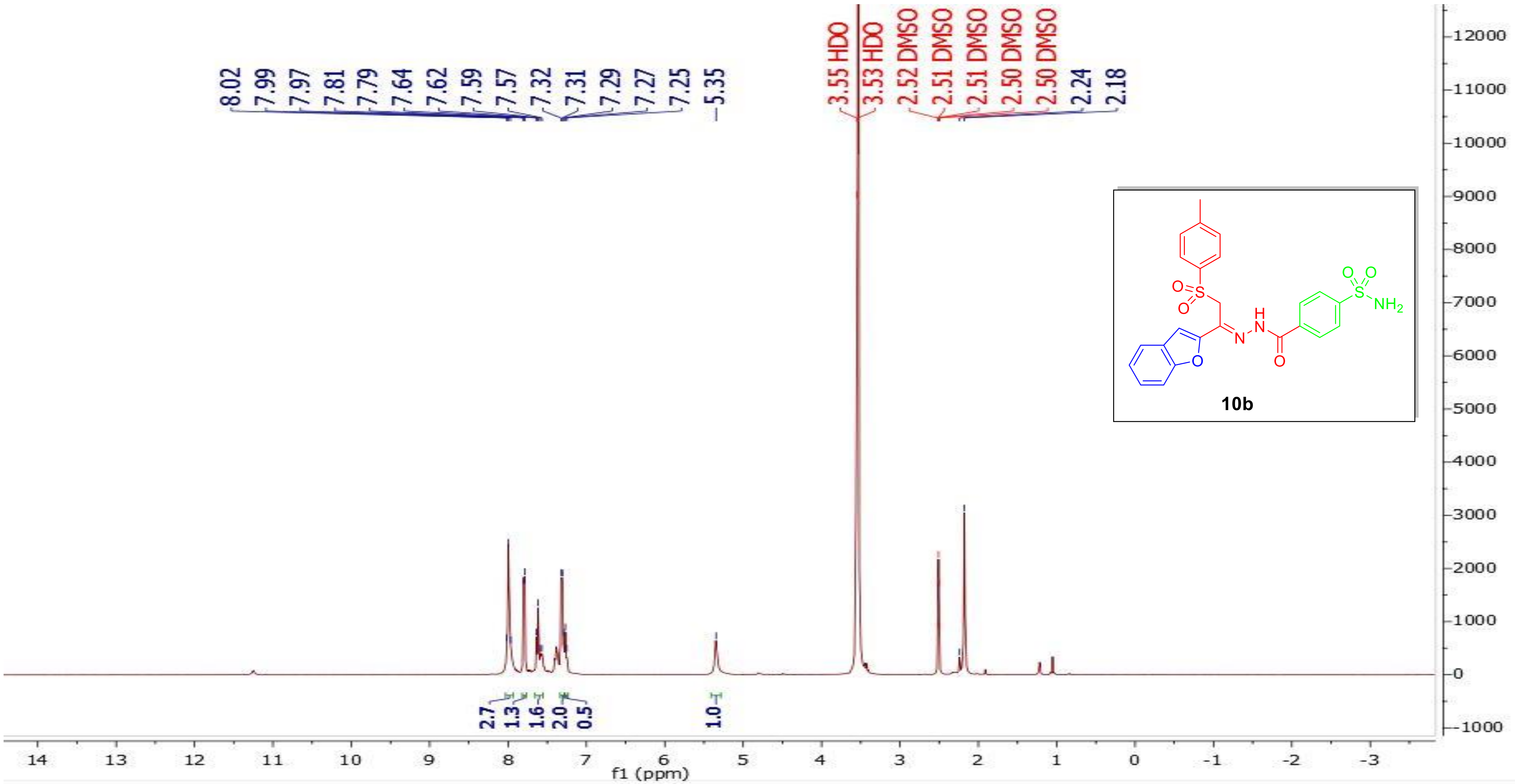
8.02
7.99
7.97
7.81
7.79
7.64
7.62
7.59
7.57
7.32
7.31
7.29
7.27
7.25
5.35
3.55 HDO
3.53 HDO
2.52 DMSO
2.51 DMSO
2.51 DMSO
2.50 DMSO
2.50 DMSO
2.24
2.18
10b
2.7
1.3
1.6
2.0
0.5
1.0
f1 (ppm)

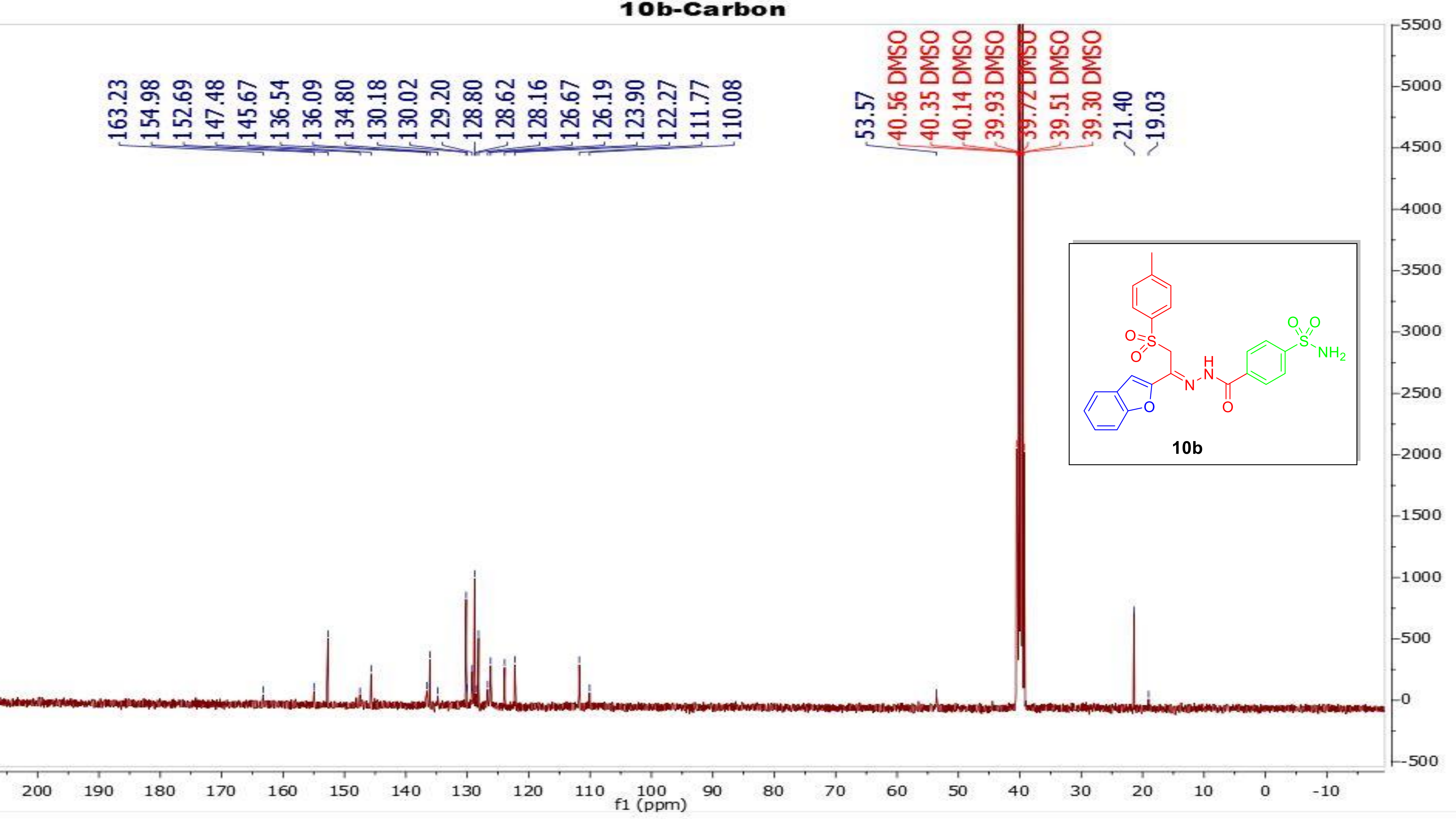
10b-Carbon
163.23
154.98
152.69
147.48
145.67
136.54
136.09
134.80
130.18
130.02
129.20
128.80
128.62
128.16
126.67
126.19
123.90
122.27
111.77
110.08
53.57
40.56 DMSO
40.35 DMSO
40.14 DMSO
39.93 DMSO
39.72 DMSO
39.51 DMSO
39.30 DMSO
21.40
19.03
10b
f1 (ppm)

10c-proton

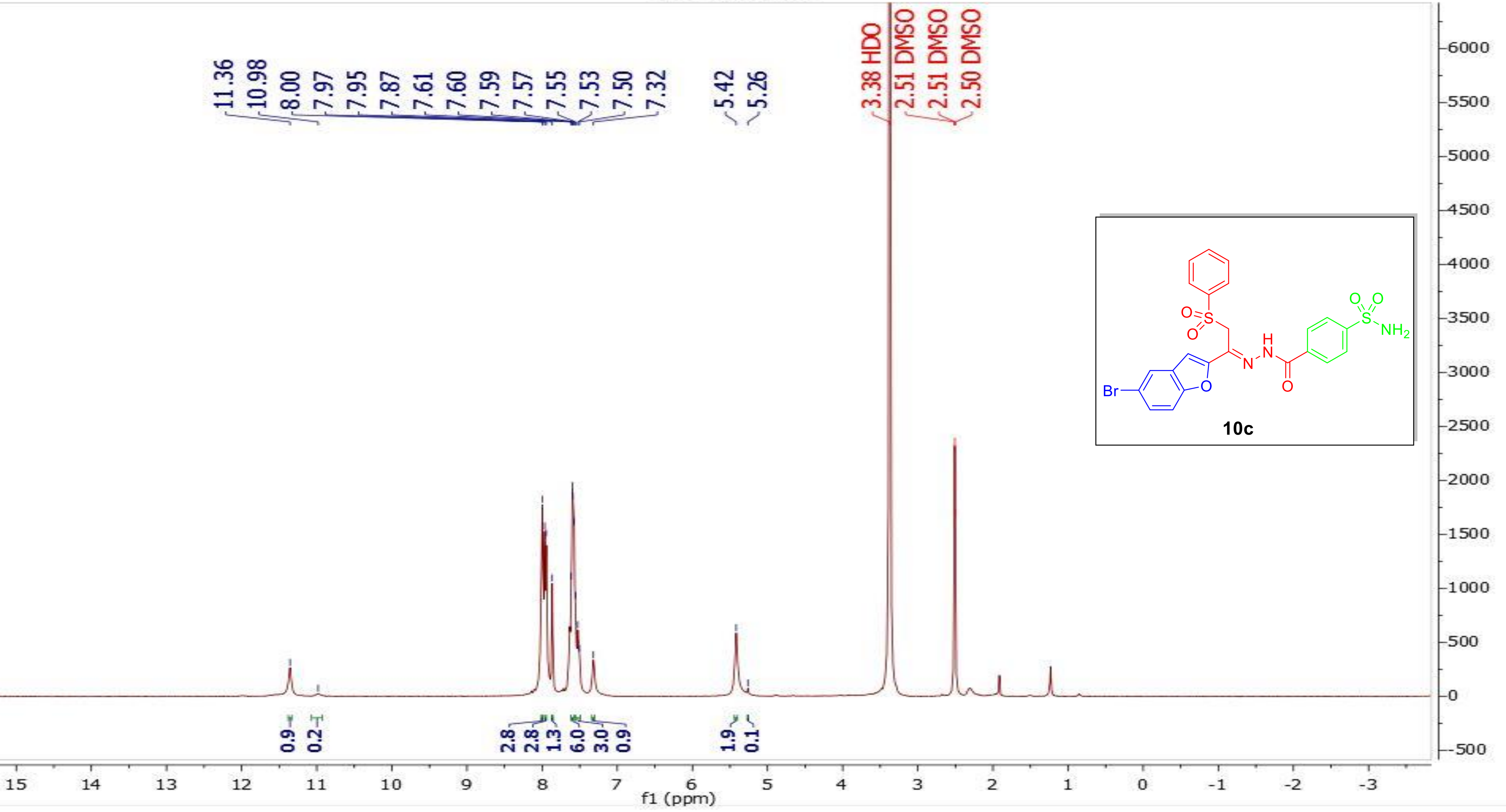
11.36
10.98
8.00
7.97
7.95
7.87
7.61
7.60
7.59
7.57
7.55
7.53
7.50
7.32
5.42
5.26
3.38 HDO
2.51 DMSO
2.51 DMSO
2.50 DMSO
0.9
0.2
2.8
2.8
1.3
6.0
3.0
0.9
1.9
0.1
Br
O
N
H
N
O
O
S
O
S
O
O
NH2
10c
f1 (ppm)

10c-D_2O

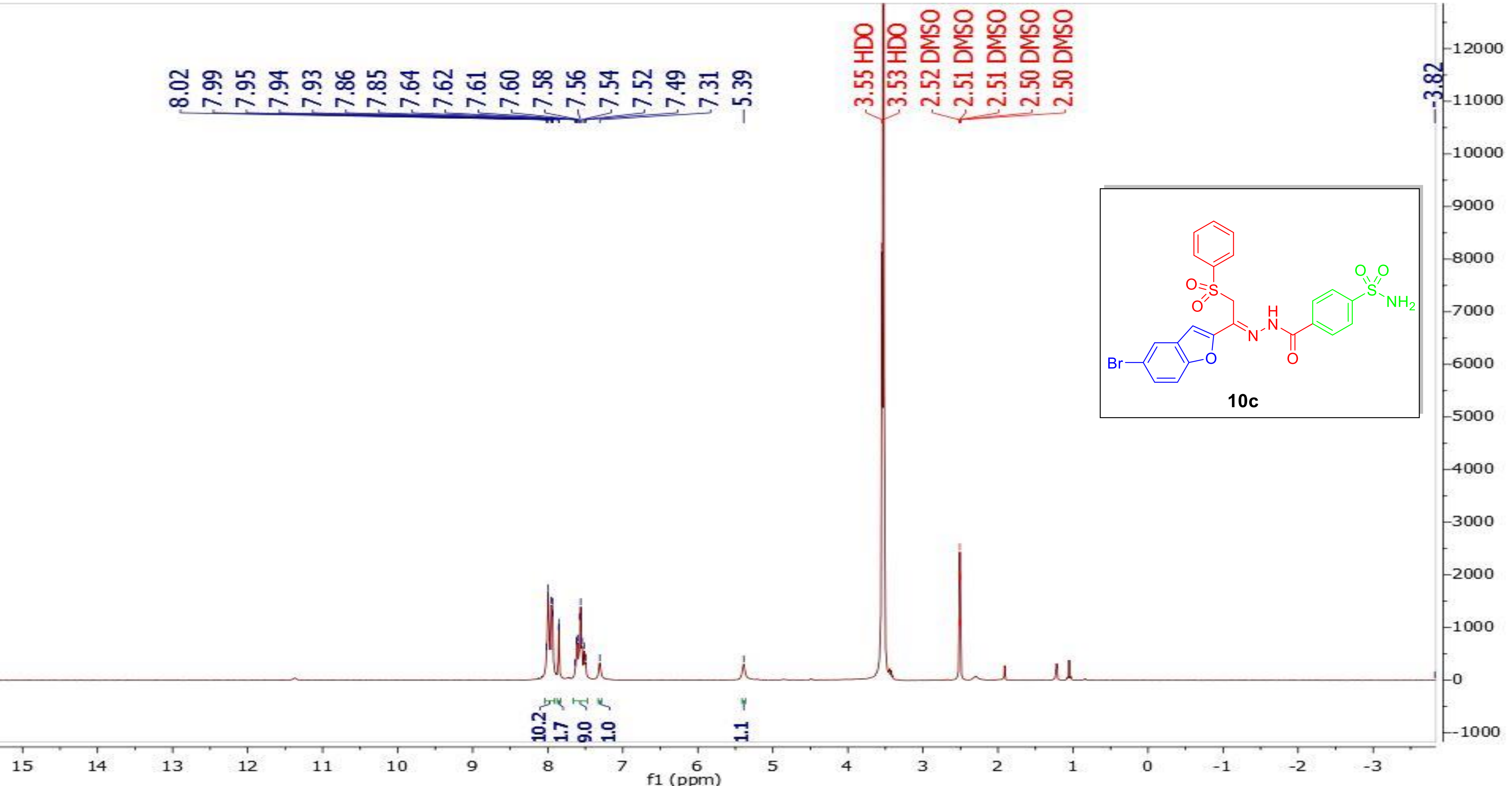
8.02
7.99
7.95
7.94
7.93
7.86
7.85
7.64
7.62
7.61
7.60
7.58
7.56
7.54
7.52
7.49
7.31
5.39
3.55 HDO
3.53 HDO
2.52 DMSO
2.51 DMSO
2.51 DMSO
2.50 DMSO
2.50 DMSO
-3.82
10.2
1.7
9.0
1.0
1.1
Br
O
O
S
O
H
N
N
O
O
O
S
NH2
10c
f1 (ppm)
15 14 13 12 11 10 9 8 7 6 5 4 3 2 1 0 -1 -2 -3
12000 11000 10000 9000 8000 7000 6000 5000 4000 3000 2000 1000 0 -1000

10c-Carbon

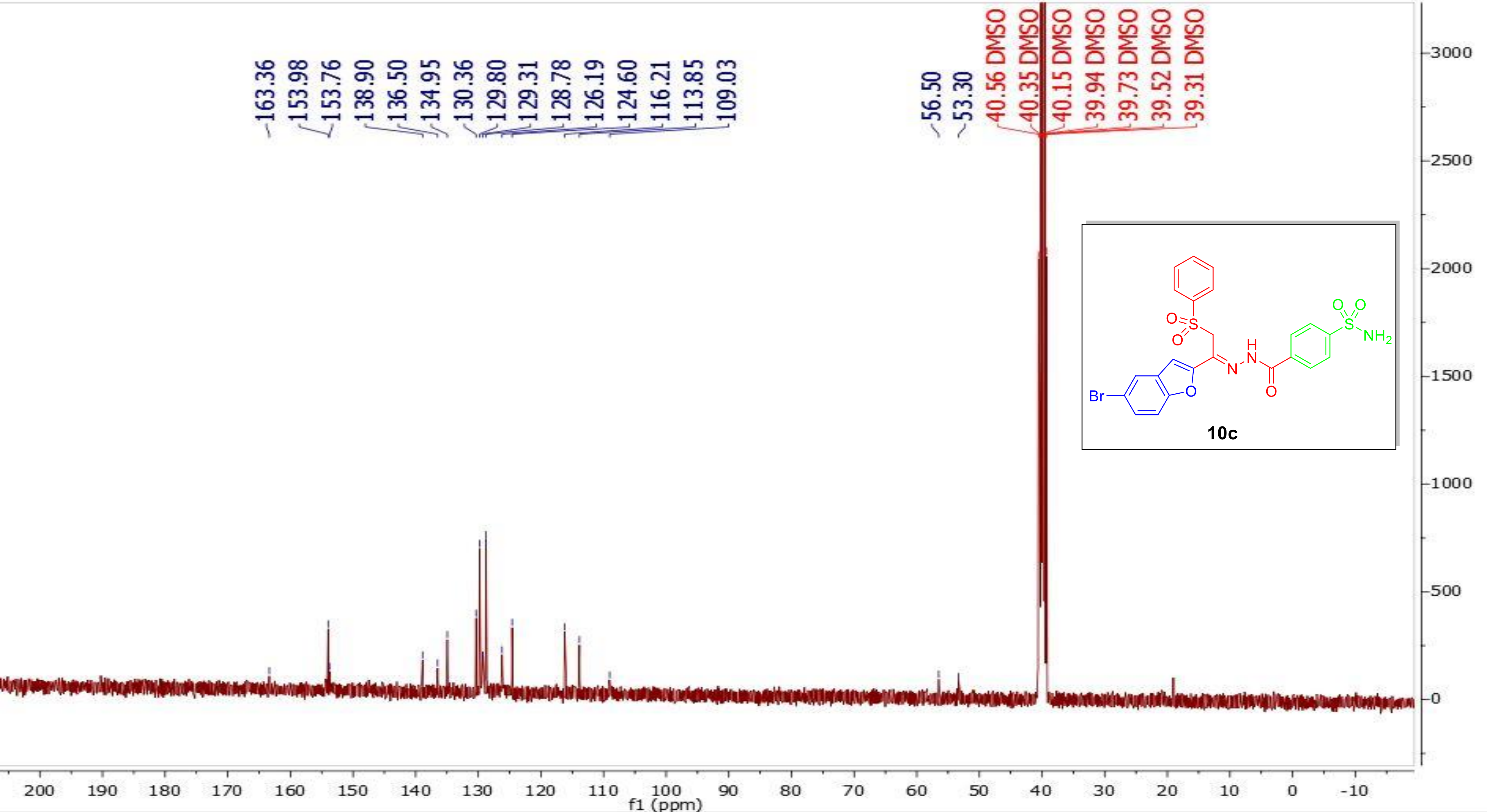
163.36
153.98
153.76
138.90
136.50
134.95
130.36
129.80
129.31
128.78
126.19
124.60
116.21
113.85
109.03
56.50
53.30
40.56 DMSO
40.35 DMSO
40.15 DMSO
39.94 DMSO
39.73 DMSO
39.52 DMSO
39.31 DMSO
Br
O
O
S
O
O
S
NH2
H
N
N
O
10c
f1 (ppm)

10d-proton

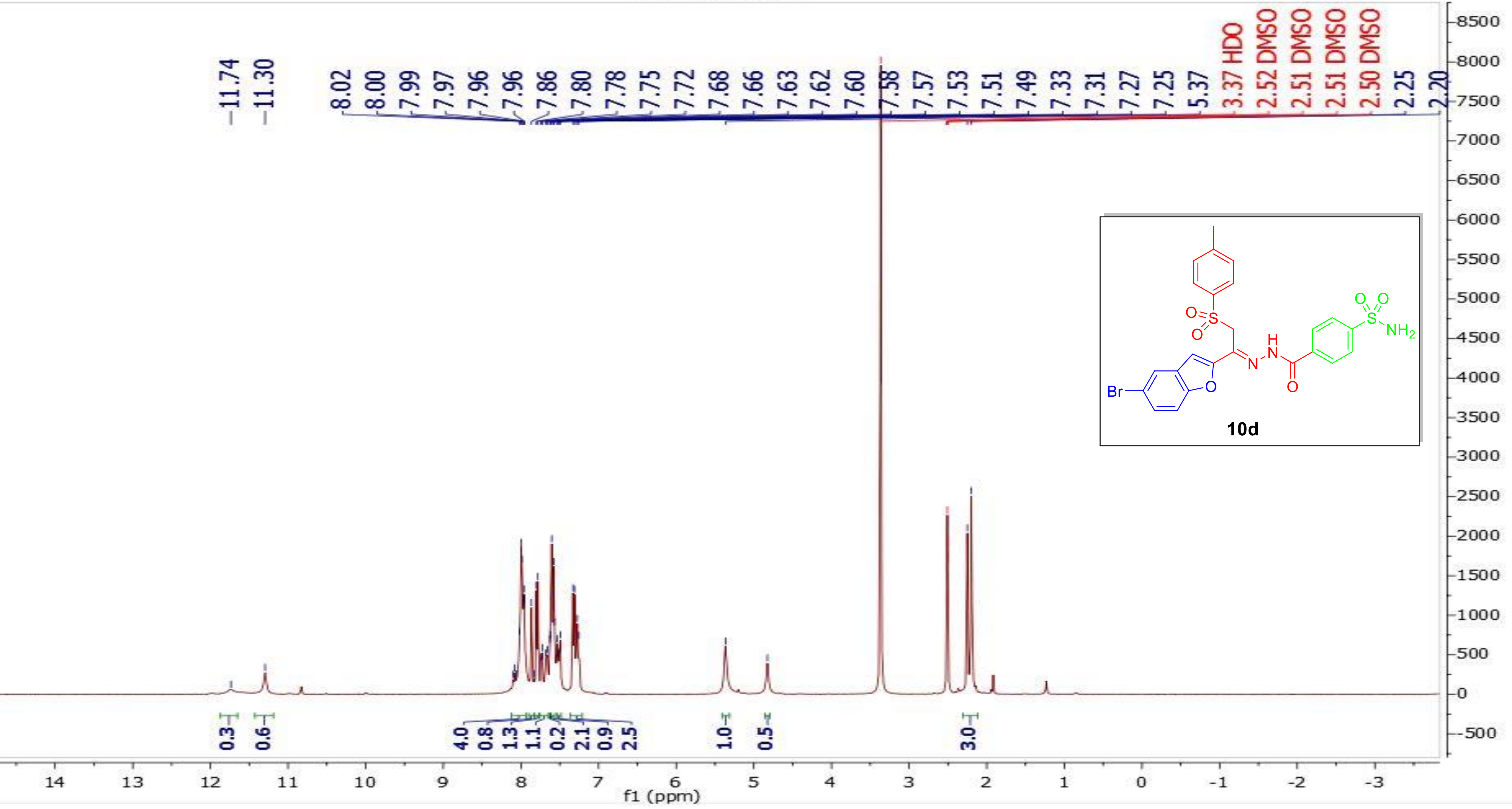

11.74
11.30
8.02
8.00
7.99
7.97
7.96
7.96
7.86
7.80
7.78
7.75
7.72
7.68
7.66
7.63
7.62
7.60
7.58
7.57
7.53
7.51
7.49
7.33
7.31
7.27
7.25
5.37
3.37 HDO
2.52 DMSO
2.51 DMSO
2.51 DMSO
2.50 DMSO
2.25
2.20
0.3
0.6
4.0
0.8
1.3
1.1
0.2
2.1
0.9
2.5
1.0
0.5
3.0
f1 (ppm)
Br
O
O
S
O
H
N
N
O
O
O
S
NH_2
10d

10d-D$_2$O

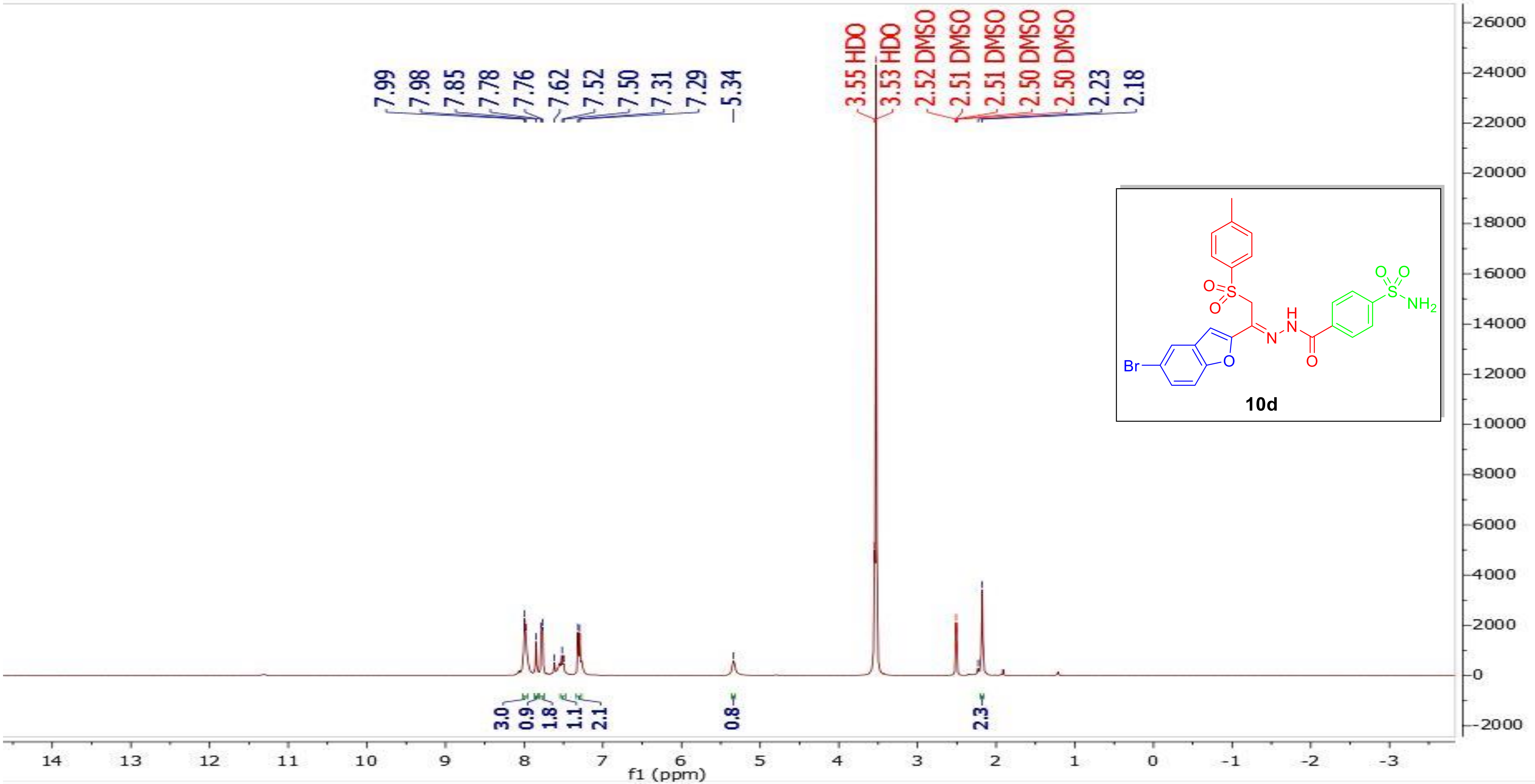

7.99
7.98
7.85
7.78
7.76
7.62
7.52
7.50
7.31
7.29
5.34
3.55 HDO
3.53 HDO
2.52 DMSO
2.51 DMSO
2.51 DMSO
2.50 DMSO
2.50 DMSO
2.23
2.18
3.0
0.9
1.8
1.1
2.1
0.8
2.3
Br
O
O
S
O
H
N
N
O
O
S
O
NH2
10d
f1 (ppm)

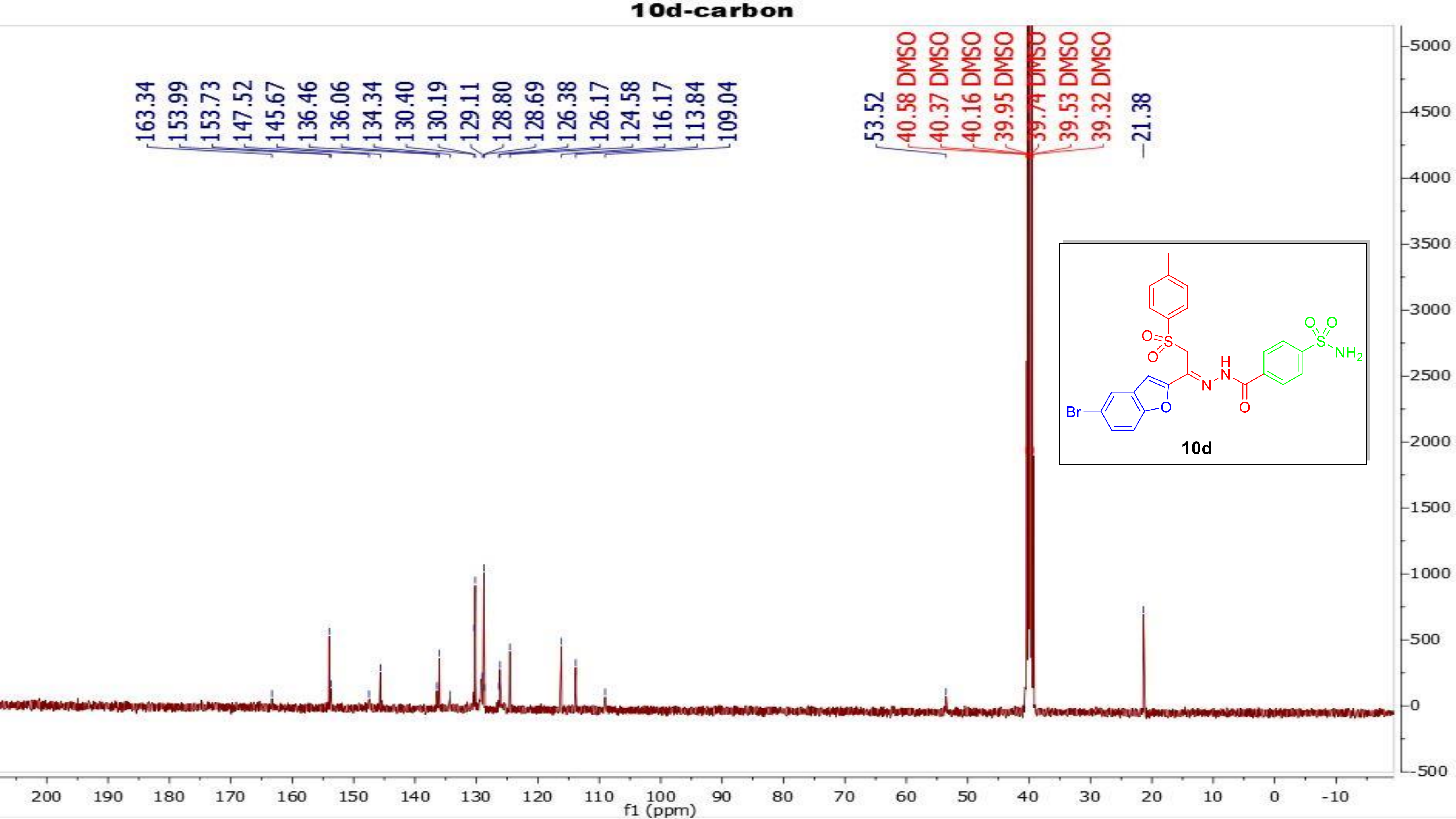
10d-carbon
163.34
153.99
153.73
147.52
145.67
136.46
136.06
134.34
130.40
130.19
129.11
128.80
128.69
126.38
126.17
124.58
116.17
113.84
109.04
53.52
40.58 DMSO
40.37 DMSO
40.16 DMSO
39.95 DMSO
39.74 DMSO
39.53 DMSO
39.32 DMSO
21.38
Br
O
N
H
N
O
O
S
O
S
O
O
NH2
10d
f1 (ppm)

Developmental Therapeutics Program

One Dose Mean Graph 4a

NSC: D-815446 / 1	Conc: 1.00E-5 Molar	Test Date: May 20, 2019
Experiment ID: 1905OS11		Report Date: Jun 16, 2019

Panel/Cell Line	Growth Percent
Leukemia	
CCRF-CEM	114.54
HL-60(TB)	110.49
K-562	104.77
MOLT-4	95.21
RPMI-8226	145.84
SR	95.61
Non-Small Cell Lung Cancer	
A549/ATCC	89.39
EKVX	96.18
HOP-62	115.36
HOP-92	140.20
NCI-H23	103.88
NCI-H322M	103.95
NCI-H460	100.67
NCI-H522	100.83
Colon Cancer	
COLO 205	124.24
HCC-2998	112.09
HCT-116	112.78
HCT-15	95.88
HT29	113.33
KM12	104.53
SW-620	117.45
CNS Cancer	
SF-268	110.58
SF-295	96.96
SF-539	95.20
SNB-19	101.01
SNB-75	109.82
U251	106.75
Melanoma	
LOX IMVI	99.71
MALME-3M	116.83
M14	109.66
MDA-MB-435	103.40
SK-MEL-2	112.43
SK-MEL-28	107.53
SK-MEL-5	93.58
UACC-257	95.85
UACC-62	97.93
Ovarian Cancer	
IGROV1	110.97
OVCAR-3	113.71
OVCAR-5	106.05
OVCAR-8	103.90
NCI/ADR-RES	111.71
SK-OV-3	107.49
Renal Cancer	
786-0	106.53
ACHN	112.68
CAKI-1	104.10
RXF 393	127.96
SN12C	103.09
TK-10	115.04
UO-31	118.30
Prostate Cancer	
PC-3	99.18
DU-145	111.60
Breast Cancer	
MCF7	92.91
MDA-MB-231/ATCC	98.72
HS 578T	109.19
BT-549	115.06
T-47D	101.80
MDA-MB-468	101.77
Mean	107.48
Delta	18.09
Range	56.45

Mean Growth Percent - Growth Percent

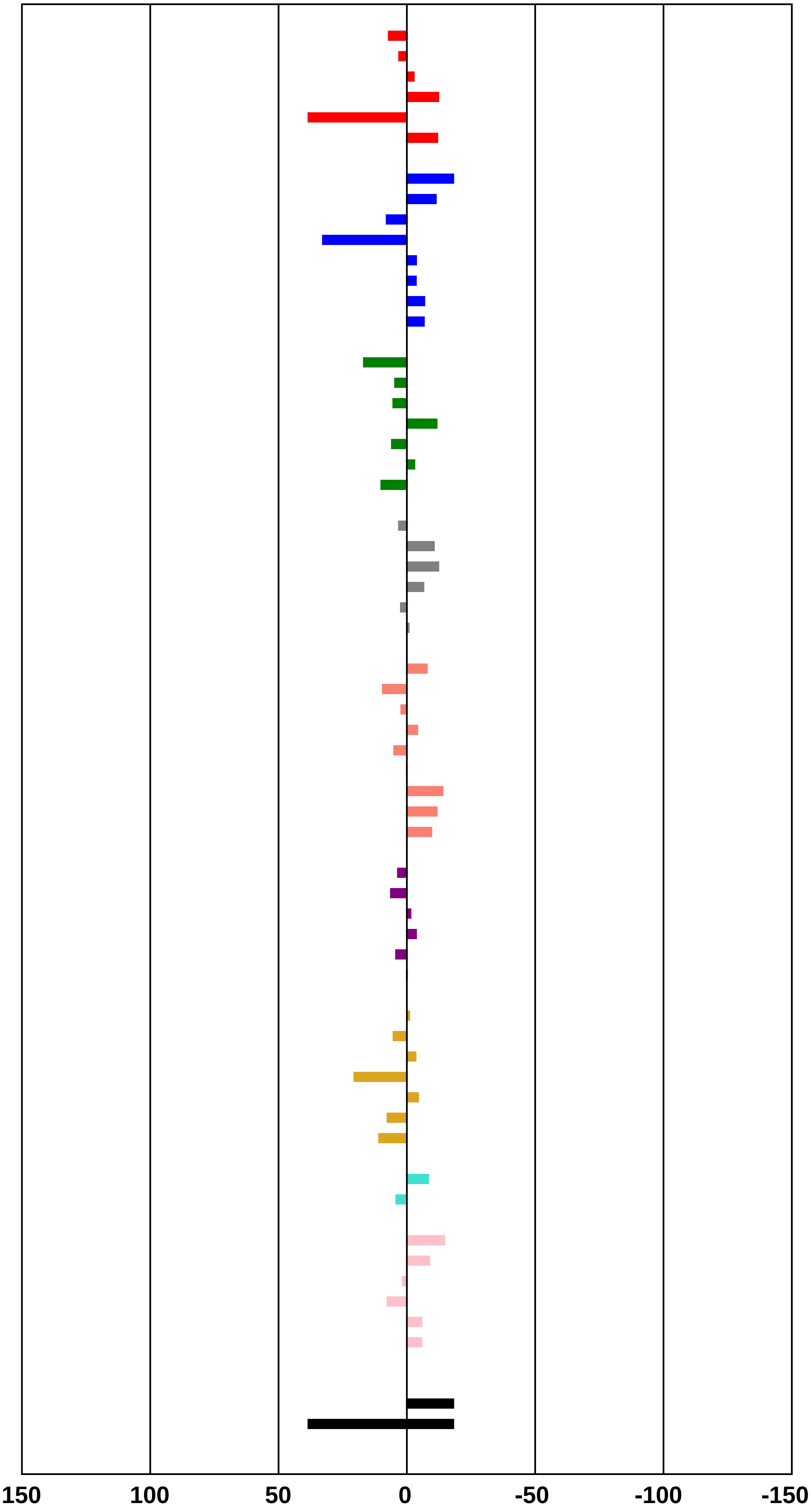

Developmental Therapeutics Program | **NSC:** D-815447 / 1 | **Conc:** 1.00E-5 Molar | **Test Date:** May 20, 2019

One Dose Mean Graph **4b** | **Experiment ID:** 1905OS11 | **Report Date:** Jun 16, 2019

Panel/Cell Line	Growth Percent
Leukemia	
CCRF-CEM	104.87
HL-60(TB)	100.40
K-562	101.29
MOLT-4	102.81
RPMI-8226	117.41
SR	104.67
Non-Small Cell Lung Cancer	
A549/ATCC	93.21
EKVX	105.70
HOP-62	123.57
HOP-92	124.66
NCI-H23	99.74
NCI-H322M	105.93
NCI-H460	102.03
NCI-H522	98.85
Colon Cancer	
COLO 205	114.78
HCC-2998	112.50
HCT-116	109.22
HCT-15	106.51
HT29	105.22
KM12	103.78
SW-620	108.43
CNS Cancer	
SF-268	113.34
SF-295	100.03
SF-539	93.90
SNB-19	105.82
SNB-75	94.46
U251	96.81
Melanoma	
LOX IMVI	96.41
MALME-3M	95.26
M14	105.51
MDA-MB-435	99.47
SK-MEL-2	115.24
SK-MEL-28	101.38
SK-MEL-5	92.00
UACC-257	100.27
UACC-62	101.89
Ovarian Cancer	
IGROV1	106.13
OVCAR-3	113.67
OVCAR-5	102.99
OVCAR-8	95.47
NCI/ADR-RES	110.70
SK-OV-3	112.90
Renal Cancer	
786-0	104.87
ACHN	99.14
CAKI-1	98.32
RXF 393	114.25
SN12C	99.22
TK-10	122.71
UO-31	100.37
Prostate Cancer	
PC-3	104.00
DU-145	108.11
Breast Cancer	
MCF7	95.19
MDA-MB-231/ATCC	95.35
HS 578T	109.57
BT-549	99.91
T-47D	99.39
MDA-MB-468	106.33
Mean	104.49
Delta	12.49
Range	32.66

Mean Growth Percent - Growth Percent

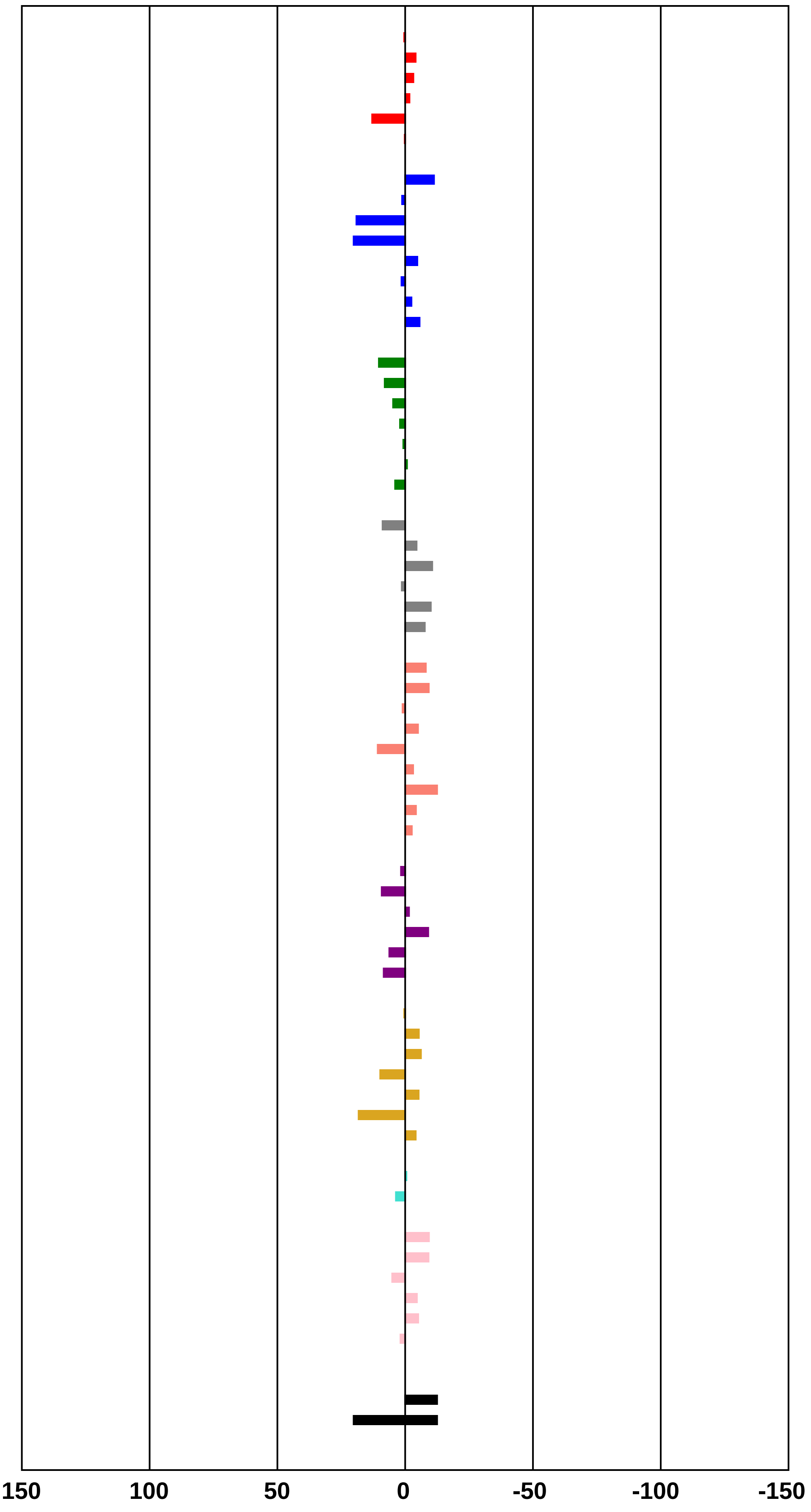

Developmental Therapeutics Program
One Dose Mean Graph 5a

NSC: D-815451 / 1	Conc: 1.00E-5 Molar	Test Date: May 20, 2019
Experiment ID: 1905OS11		Report Date: Jun 16, 2019

Panel/Cell Line	Growth Percent
**Leukemia**	
CCRF-CEM	102.56
HL-60(TB)	117.85
K-562	111.35
MOLT-4	121.84
RPMI-8226	117.17
SR	122.69
**Non-Small Cell Lung Cancer**	
A549/ATCC	104.23
EKVX	104.22
HOP-62	115.38
HOP-92	111.50
NCI-H23	95.77
NCI-H322M	106.52
NCI-H460	100.35
NCI-H522	98.60
**Colon Cancer**	
COLO 205	109.81
HCC-2998	102.87
HCT-116	103.36
HCT-15	104.22
HT29	103.50
KM12	103.89
SW-620	101.00
**CNS Cancer**	
SF-268	104.57
SF-295	100.82
SF-539	90.60
SNB-19	100.69
SNB-75	88.17
U251	98.37
**Melanoma**	
LOX IMVI	100.36
MALME-3M	88.16
M14	99.96
MDA-MB-435	103.31
SK-MEL-2	112.84
SK-MEL-28	104.14
SK-MEL-5	101.80
UACC-257	94.45
UACC-62	103.51
**Ovarian Cancer**	
IGROV1	109.90
OVCAR-3	105.30
OVCAR-5	98.30
OVCAR-8	99.43
NCI/ADR-RES	103.21
SK-OV-3	106.01
**Renal Cancer**	
786-0	104.00
ACHN	106.02
CAKI-1	100.53
RXF 393	114.98
SN12C	101.93
TK-10	109.05
UO-31	93.78
**Prostate Cancer**	
PC-3	103.12
DU-145	105.85
**Breast Cancer**	
MCF7	93.46
MDA-MB-231/ATCC	109.91
HS 578T	97.79
BT-549	95.24
T-47D	101.38
MDA-MB-468	99.57
Mean	103.67
Delta	15.51
Range	34.53

Mean Growth Percent - Growth Percent

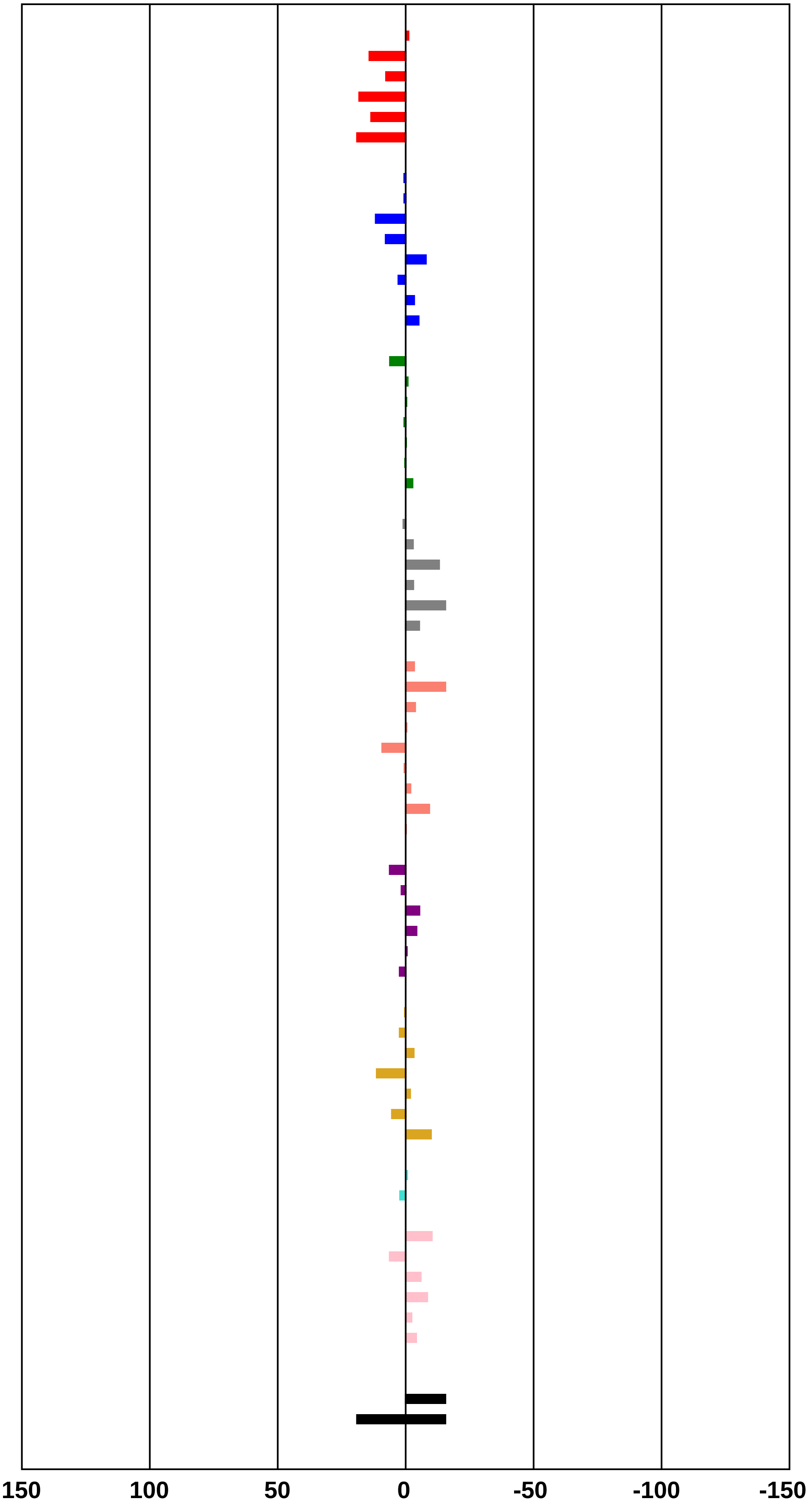

Developmental Therapeutics Program

One Dose Mean Graph 5b

NSC: D-815452 / 1	Conc: 1.00E-5 Molar	Test Date: May 20, 2019
Experiment ID: 1905OS11		Report Date: Jun 16, 2019

Panel/Cell Line	Growth Percent
**Leukemia**	
CCRF-CEM	98.64
HL-60(TB)	102.64
K-562	85.35
MOLT-4	61.40
RPMI-8226	114.65
SR	95.33
**Non-Small Cell Lung Cancer**	
A549/ATCC	66.97
EKVX	85.54
HOP-62	95.28
HOP-92	111.10
NCI-H23	96.93
NCI-H322M	81.81
NCI-H460	96.41
NCI-H522	91.74
**Colon Cancer**	
COLO 205	105.10
HCC-2998	86.56
HCT-116	92.74
HCT-15	69.87
HT29	87.16
KM12	62.22
SW-620	100.71
**CNS Cancer**	
SF-268	77.52
SF-295	99.08
SF-539	85.99
SNB-19	90.58
SNB-75	79.33
U251	84.46
**Melanoma**	
LOX IMVI	86.30
MALME-3M	102.03
M14	84.57
MDA-MB-435	87.78
SK-MEL-2	101.34
SK-MEL-28	97.97
SK-MEL-5	94.54
UACC-257	89.19
UACC-62	82.72
**Ovarian Cancer**	
IGROV1	89.44
OVCAR-3	99.56
OVCAR-5	87.48
OVCAR-8	91.92
NCI/ADR-RES	99.03
SK-OV-3	102.32
**Renal Cancer**	
786-0	97.42
ACHN	88.57
CAKI-1	61.29
RXF 393	121.48
SN12C	94.46
TK-10	97.62
UO-31	93.45
**Prostate Cancer**	
PC-3	81.07
DU-145	88.03
**Breast Cancer**	
MCF7	78.92
MDA-MB-231/ATCC	80.76
HS 578T	91.73
BT-549	94.37
T-47D	70.56
MDA-MB-468	88.05
Mean	89.98
Delta	28.69
Range	60.19

Mean Growth Percent - Growth Percent

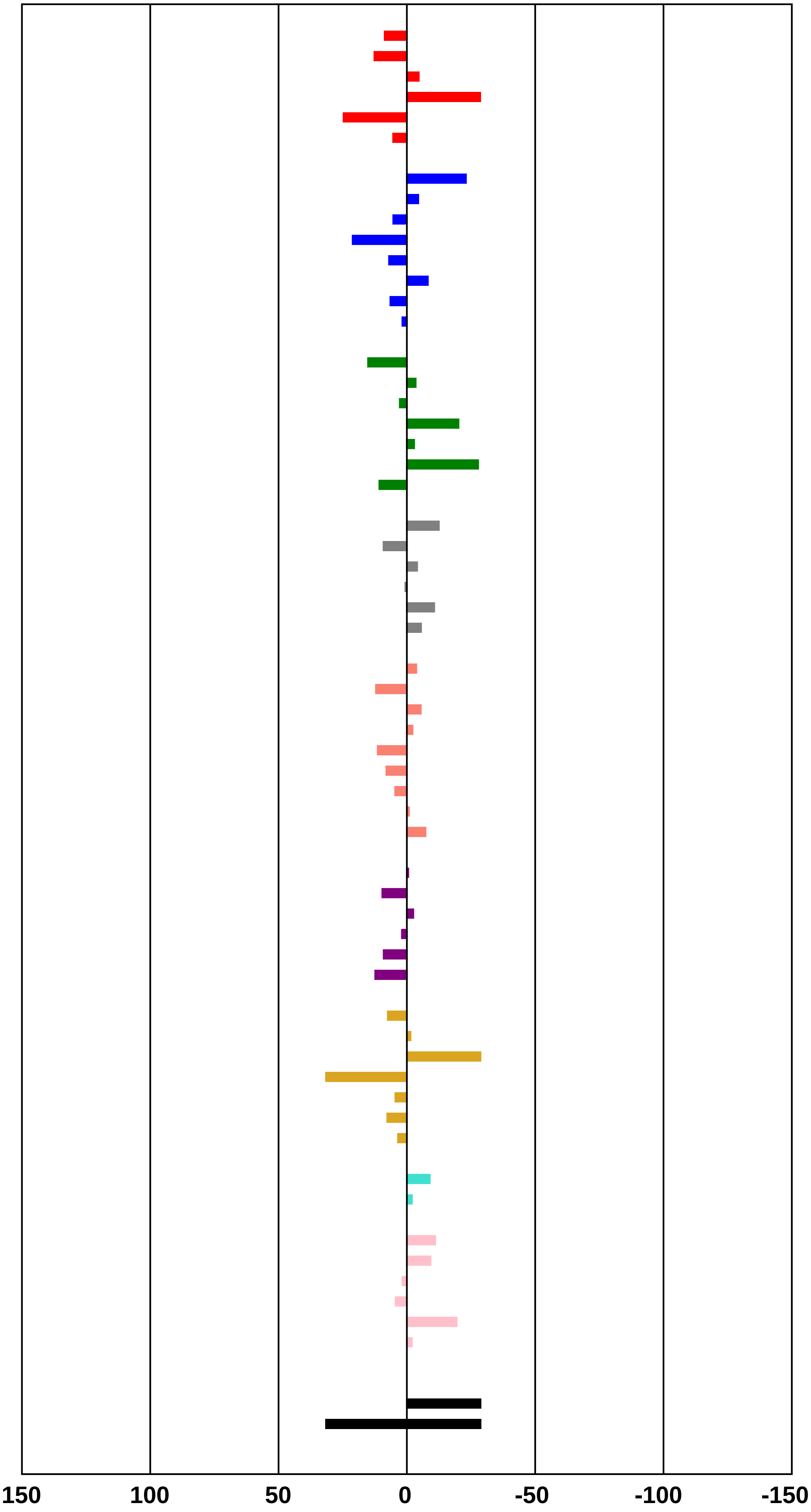

Developmental Therapeutics Program

One Dose Mean Graph 9a

NSC: D-815429 / 1	Conc: 1.00E-5 Molar	Test Date: May 20, 2019
Experiment ID: 1905OS11		Report Date: Jun 16, 2019

Panel/Cell Line	Growth Percent
**Leukemia**	
CCRF-CEM	101.55
HL-60(TB)	101.07
K-562	105.01
MOLT-4	110.67
RPMI-8226	97.79
SR	113.93
**Non-Small Cell Lung Cancer**	
A549/ATCC	82.10
EKVX	96.19
HOP-62	102.58
HOP-92	117.84
NCI-H23	101.63
NCI-H322M	102.37
NCI-H460	100.39
NCI-H522	94.48
**Colon Cancer**	
COLO 205	110.30
HCC-2998	105.12
HCT-116	97.92
HCT-15	98.15
HT29	106.22
KM12	106.79
SW-620	107.52
**CNS Cancer**	
SF-268	102.87
SF-295	99.24
SF-539	97.47
SNB-19	95.72
SNB-75	84.68
U251	90.34
**Melanoma**	
LOX IMVI	98.01
MALME-3M	101.99
M14	101.53
MDA-MB-435	100.87
SK-MEL-2	101.40
SK-MEL-28	105.42
SK-MEL-5	101.24
UACC-257	69.77
UACC-62	89.05
**Ovarian Cancer**	
IGROV1	102.07
OVCAR-3	107.22
OVCAR-5	95.34
OVCAR-8	90.21
NCI/ADR-RES	103.14
SK-OV-3	109.84
**Renal Cancer**	
786-0	100.25
ACHN	101.11
CAKI-1	87.07
RXF 393	109.85
SN12C	99.34
TK-10	99.89
UO-31	88.23
**Prostate Cancer**	
PC-3	91.45
DU-145	105.24
**Breast Cancer**	
MCF7	89.84
MDA-MB-231/ATCC	92.12
HS 578T	100.77
BT-549	94.17
T-47D	90.13
MDA-MB-468	107.08
Mean	99.36
Delta	29.59
Range	48.07

Mean Growth Percent - Growth Percent

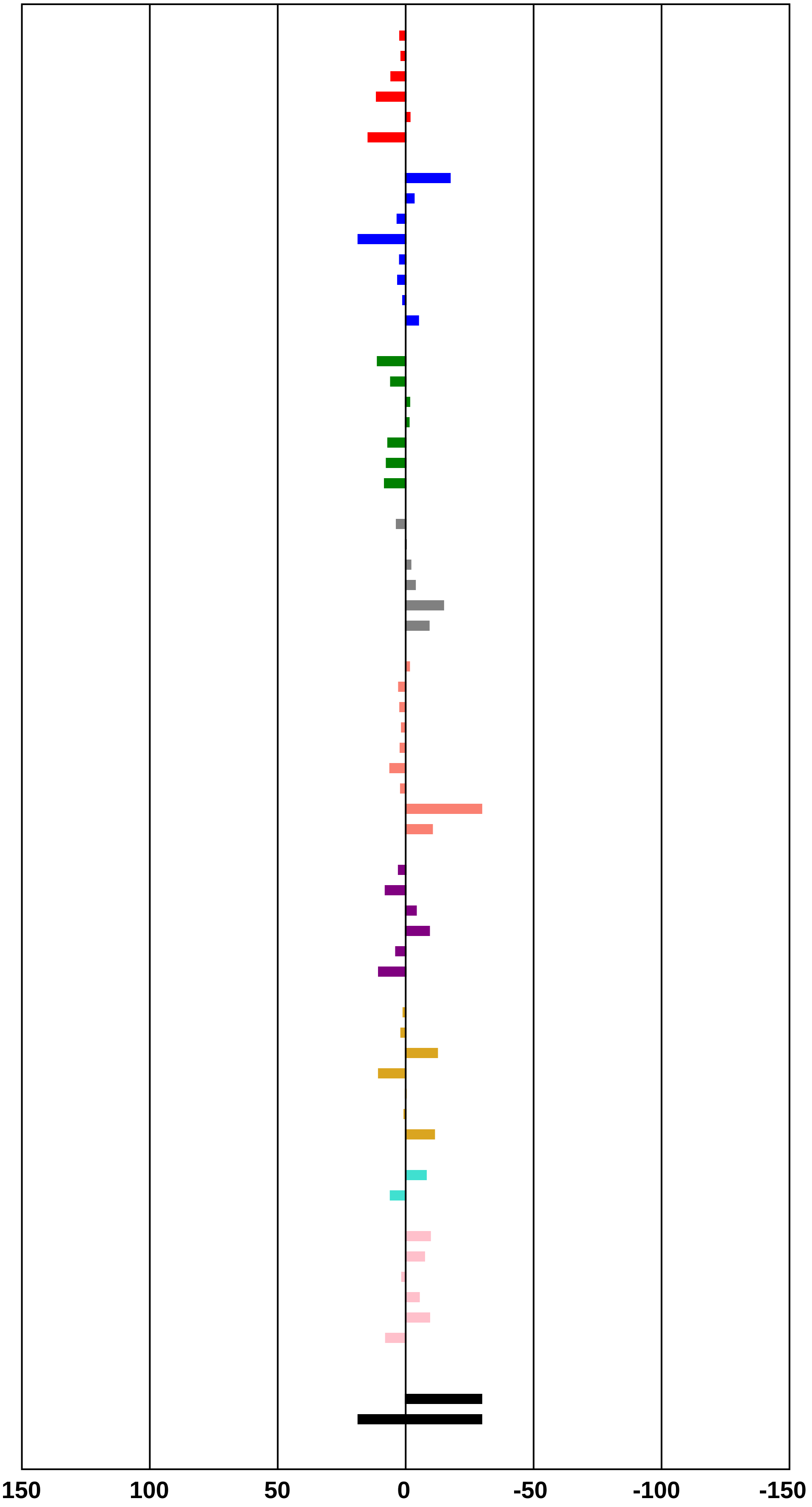

Developmental Therapeutics Program

One Dose Mean Graph 9b

NSC: D-815430 / 1 | **Conc:** 1.00E-5 Molar | **Test Date:** May 20, 2019

Experiment ID: 1905OS11 | **Report Date:** Jun 16, 2019

Panel/Cell Line	Growth Percent
**Leukemia**	
CCRF-CEM	112.44
HL-60(TB)	109.52
K-562	105.07
MOLT-4	98.54
RPMI-8226	126.72
SR	106.72
**Non-Small Cell Lung Cancer**	
A549/ATCC	87.27
EKVX	96.40
HOP-62	108.92
HOP-92	108.76
NCI-H23	99.92
NCI-H322M	101.12
NCI-H460	100.32
NCI-H522	91.07
**Colon Cancer**	
COLO 205	109.16
HCC-2998	107.56
HCT-116	104.36
HCT-15	98.32
HT29	101.08
KM12	106.78
SW-620	110.61
**CNS Cancer**	
SF-268	99.51
SF-295	95.86
SF-539	94.30
SNB-19	95.59
SNB-75	85.26
U251	91.28
**Melanoma**	
LOX IMVI	99.48
MALME-3M	99.12
M14	99.84
MDA-MB-435	102.33
SK-MEL-2	99.50
SK-MEL-28	102.29
SK-MEL-5	94.00
UACC-257	87.71
UACC-62	96.35
**Ovarian Cancer**	
IGROV1	102.72
OVCAR-3	112.42
OVCAR-5	99.01
OVCAR-8	94.54
NCI/ADR-RES	109.33
SK-OV-3	101.29
**Renal Cancer**	
786-0	98.41
ACHN	106.62
CAKI-1	91.01
RXF 393	105.89
SN12C	97.81
TK-10	100.02
UO-31	86.42
**Prostate Cancer**	
PC-3	94.17
DU-145	109.72
**Breast Cancer**	
MCF7	90.88
MDA-MB-231/ATCC	95.77
HS 578T	100.94
BT-549	93.82
T-47D	83.58
MDA-MB-468	106.25
Mean	100.24
Delta	16.66
Range	43.14

Mean Growth Percent - Growth Percent

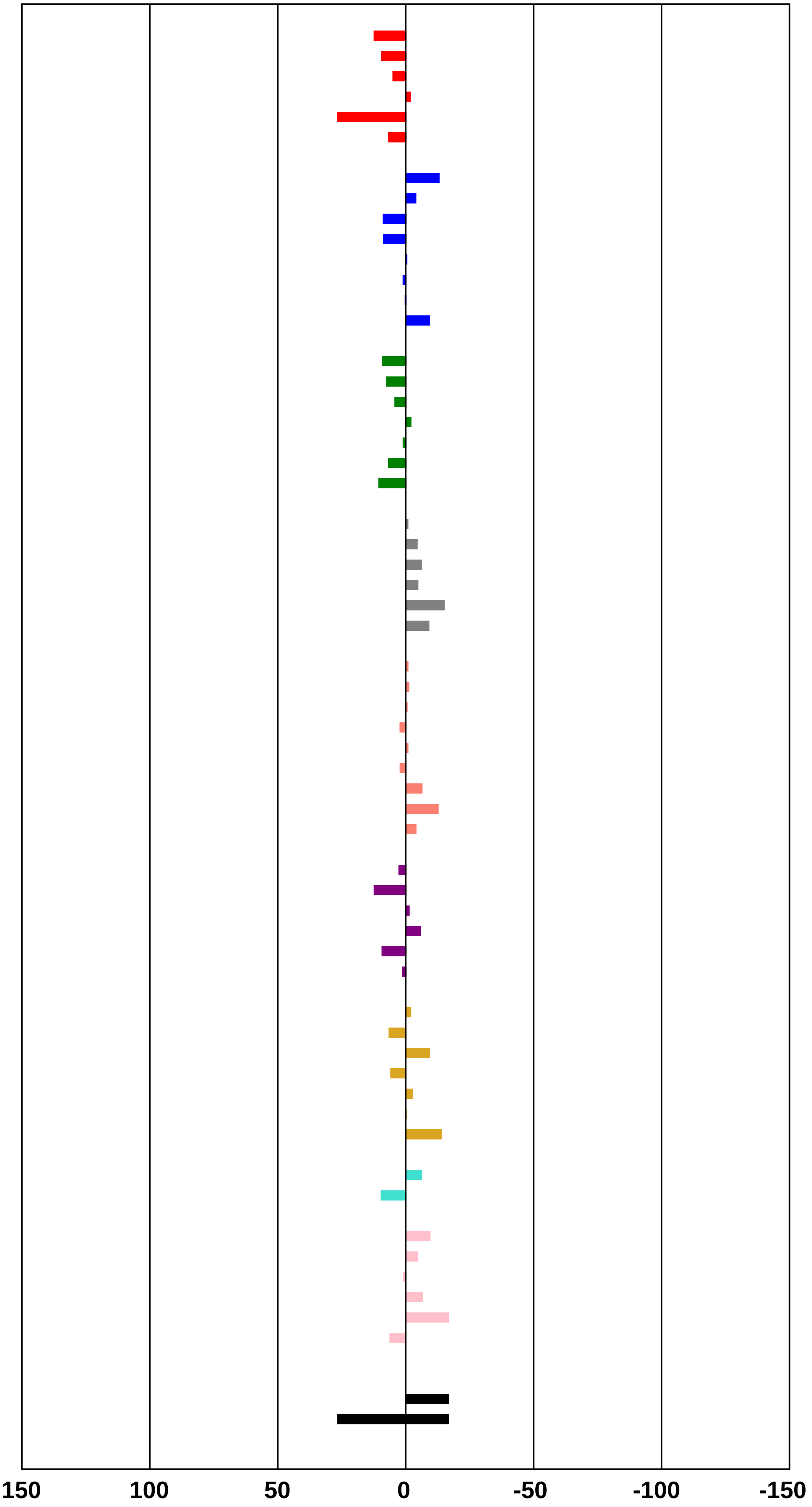

Developmental Therapeutics Program	NSC: D-815431 / 1	Conc: 1.00E-5 Molar	Test Date: May 20, 2019
One Dose Mean Graph 9c	Experiment ID: 1905OS11		Report Date: Jun 16, 2019

Panel/Cell Line	Growth Percent
**Leukemia**	
CCRF-CEM	110.69
HL-60(TB)	105.32
K-562	105.99
MOLT-4	102.56
RPMI-8226	115.58
SR	98.37
**Non-Small Cell Lung Cancer**	
A549/ATCC	87.66
EKVX	96.97
HOP-62	117.68
HOP-92	112.45
NCI-H23	102.42
NCI-H322M	102.35
NCI-H460	101.26
NCI-H522	95.30
**Colon Cancer**	
COLO 205	112.03
HCC-2998	105.34
HCT-116	109.04
HCT-15	104.89
HT29	104.85
KM12	108.38
SW-620	106.04
**CNS Cancer**	
SF-268	101.46
SF-295	97.71
SF-539	92.47
SNB-19	98.05
SNB-75	90.86
U251	92.06
**Melanoma**	
LOX IMVI	97.64
MALME-3M	94.50
M14	99.85
MDA-MB-435	102.78
SK-MEL-2	99.95
SK-MEL-28	104.90
SK-MEL-5	95.62
UACC-257	86.69
UACC-62	98.69
**Ovarian Cancer**	
IGROV1	104.34
OVCAR-3	112.17
OVCAR-5	101.92
OVCAR-8	93.96
NCI/ADR-RES	106.01
SK-OV-3	109.76
**Renal Cancer**	
786-0	100.77
ACHN	101.66
CAKI-1	88.80
RXF 393	111.97
SN12C	99.21
TK-10	107.59
UO-31	86.73
**Prostate Cancer**	
PC-3	99.99
DU-145	105.59
**Breast Cancer**	
MCF7	94.18
MDA-MB-231/ATCC	95.54
HS 578T	106.42
BT-549	92.35
T-47D	85.59
MDA-MB-468	103.09
Mean	101.16
Delta	15.57
Range	32.09

Mean Growth Percent - Growth Percent

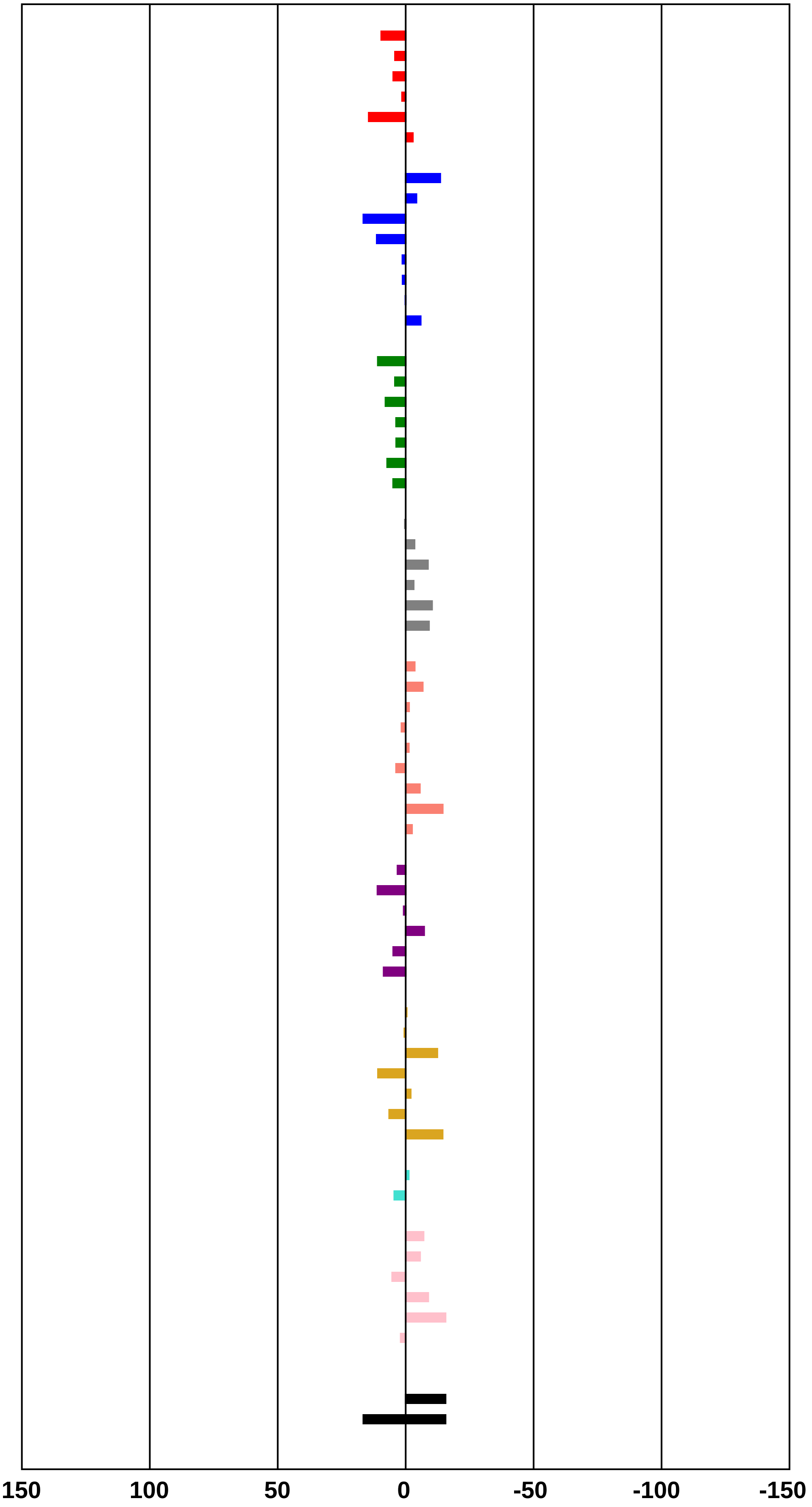

Developmental Therapeutics Program

One Dose Mean Graph 10a

NSC: D-815439 / 1	Conc: 1.00E-5 Molar	Test Date: May 20, 2019
Experiment ID: 1905OS11		Report Date: Jun 16, 2019

Panel/Cell Line	Growth Percent
**Leukemia**	
CCRF-CEM	110.19
HL-60(TB)	103.31
K-562	105.83
MOLT-4	110.64
RPMI-8226	104.76
SR	97.76
**Non-Small Cell Lung Cancer**	
A549/ATCC	93.00
EKVX	99.95
HOP-62	122.47
HOP-92	115.29
NCI-H23	101.23
NCI-H322M	101.83
NCI-H460	100.28
NCI-H522	98.47
**Colon Cancer**	
COLO 205	110.84
HCC-2998	108.07
HCT-116	109.61
HCT-15	104.42
HT29	107.84
KM12	101.99
SW-620	104.35
**CNS Cancer**	
SF-268	96.14
SF-295	98.22
SF-539	97.62
SNB-19	100.18
SNB-75	96.32
U251	98.78
**Melanoma**	
LOX IMVI	102.26
MALME-3M	99.58
M14	109.24
MDA-MB-435	96.54
SK-MEL-2	110.81
SK-MEL-28	101.13
SK-MEL-5	93.45
UACC-257	99.24
UACC-62	96.69
**Ovarian Cancer**	
IGROV1	102.50
OVCAR-3	108.87
OVCAR-5	101.76
OVCAR-8	97.26
NCI/ADR-RES	105.48
SK-OV-3	108.60
**Renal Cancer**	
786-0	102.60
ACHN	99.95
CAKI-1	89.67
RXF 393	91.72
SN12C	97.90
TK-10	113.74
UO-31	91.59
**Prostate Cancer**	
PC-3	104.78
DU-145	111.07
**Breast Cancer**	
MCF7	94.85
MDA-MB-231/ATCC	94.25
HS 578T	104.58
BT-549	96.35
T-47D	105.23
MDA-MB-468	97.66
Mean	102.26
Delta	12.59
Range	32.80

Mean Growth Percent - Growth Percent

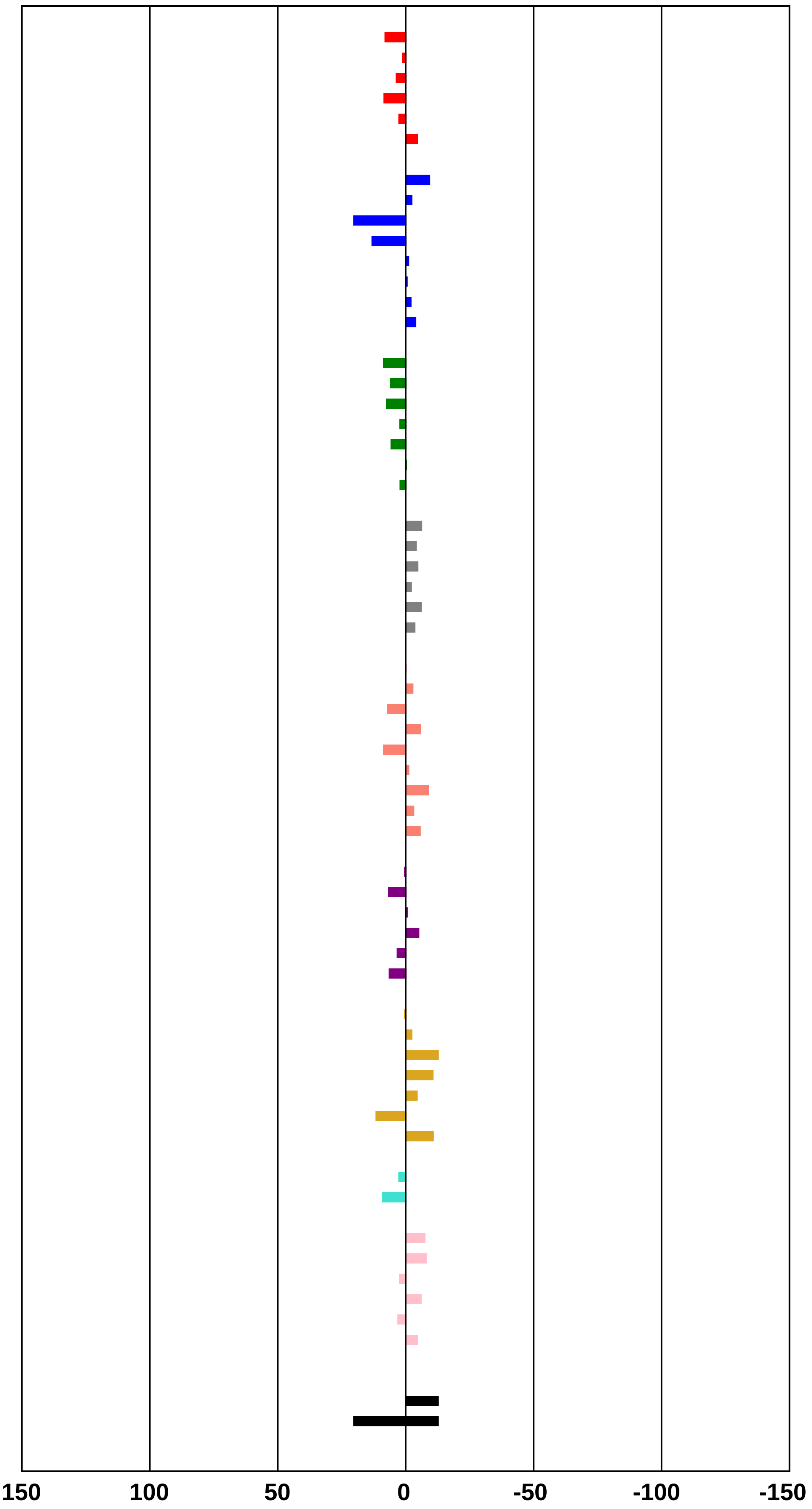

Developmental Therapeutics Program

One Dose Mean Graph 10b

NSC: D-815440 / 1	Conc: 1.00E-5 Molar	Test Date: May 20, 2019
Experiment ID: 1905OS11		Report Date: Jun 16, 2019

Panel/Cell Line	Growth Percent
**Leukemia**	
CCRF-CEM	117.51
HL-60(TB)	115.41
K-562	105.82
MOLT-4	136.63
RPMI-8226	98.29
SR	126.60
**Non-Small Cell Lung Cancer**	
A549/ATCC	87.01
EKVX	103.27
HOP-62	96.03
HOP-92	77.22
NCI-H23	80.67
NCI-H322M	103.13
NCI-H460	96.32
NCI-H522	80.23
**Colon Cancer**	
COLO 205	107.10
HCC-2998	94.20
HCT-116	90.47
HCT-15	99.47
HT29	97.40
KM12	85.61
SW-620	94.63
**CNS Cancer**	
SF-268	73.30
SF-295	101.22
SF-539	80.60
SNB-19	69.85
SNB-75	56.08
U251	96.09
**Melanoma**	
LOX IMVI	94.66
MALME-3M	90.46
M14	111.14
MDA-MB-435	73.00
SK-MEL-2	100.15
SK-MEL-28	98.40
SK-MEL-5	85.60
UACC-257	94.81
UACC-62	81.98
**Ovarian Cancer**	
IGROV1	92.51
OVCAR-3	94.55
OVCAR-5	99.80
OVCAR-8	75.18
NCI/ADR-RES	98.01
SK-OV-3	103.09
**Renal Cancer**	
786-0	100.59
ACHN	99.30
CAKI-1	89.90
RXF 393	74.74
SN12C	88.96
TK-10	107.22
UO-31	101.43
**Prostate Cancer**	
PC-3	94.38
DU-145	101.07
**Breast Cancer**	
MCF7	72.27
MDA-MB-231/ATCC	73.70
HS 578T	80.71
BT-549	100.60
T-47D	88.12
MDA-MB-468	78.91
Mean	93.25
Delta	37.17
Range	80.55

Mean Growth Percent - Growth Percent

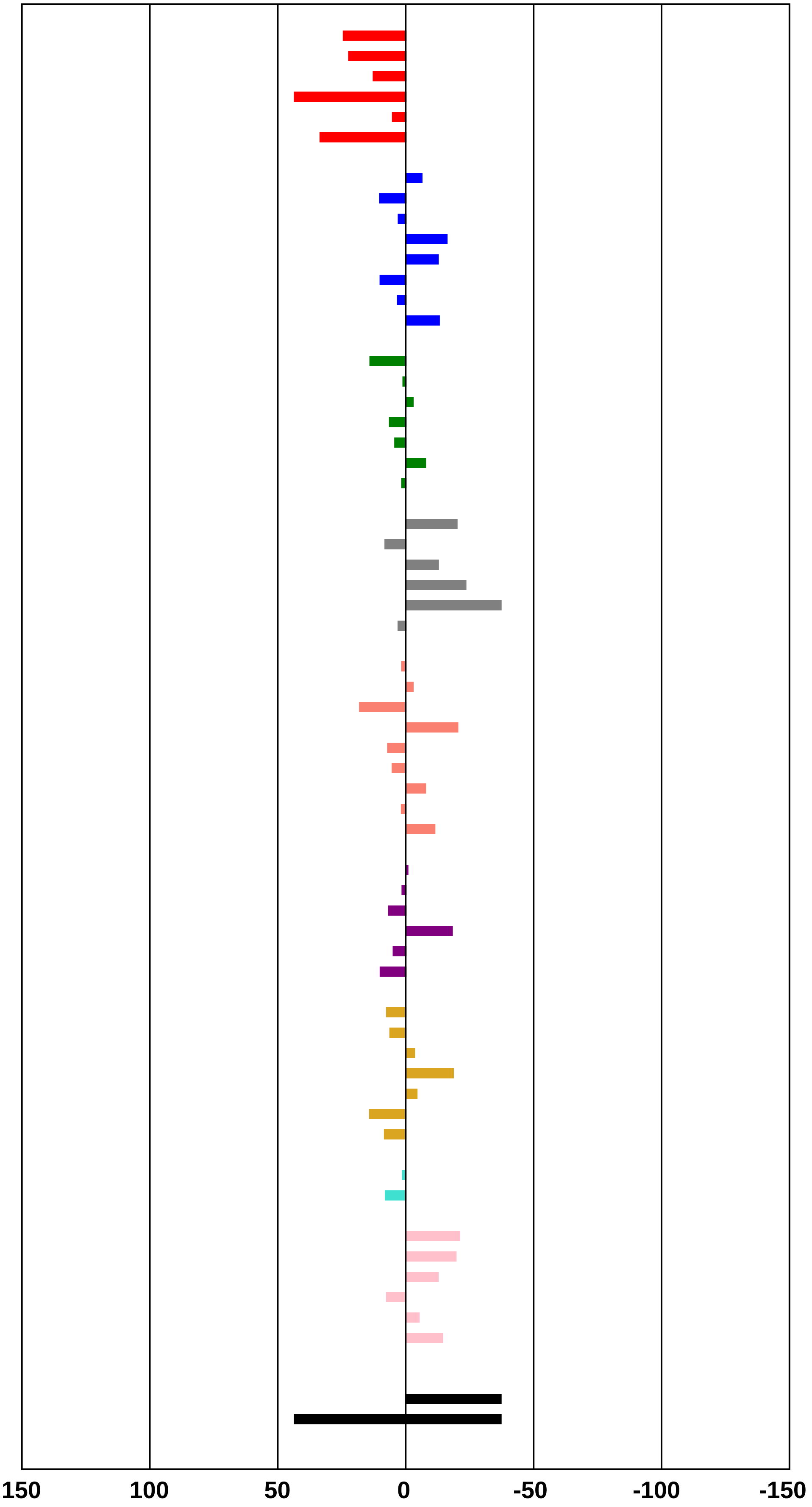

Developmental Therapeutics Program | **NSC:** D-815441 / 1 | **Conc:** 1.00E-5 Molar | **Test Date:** May 20, 2019

One Dose Mean Graph **10c** | **Experiment ID:** 1905OS11 | **Report Date:** Jun 16, 2019

Panel/Cell Line	Growth Percent
**Leukemia**	
CCRF-CEM	115.64
HL-60(TB)	103.87
K-562	96.87
MOLT-4	97.16
RPMI-8226	89.19
SR	99.78
**Non-Small Cell Lung Cancer**	
A549/ATCC	95.04
EKVX	95.01
HOP-62	105.44
HOP-92	75.11
NCI-H23	94.38
NCI-H322M	94.90
NCI-H460	99.09
NCI-H522	91.61
**Colon Cancer**	
COLO 205	97.84
HCC-2998	96.39
HCT-116	104.16
HCT-15	101.26
HT29	99.00
KM12	108.54
SW-620	92.88
**CNS Cancer**	
SF-268	97.83
SF-295	92.98
SF-539	91.05
SNB-19	96.53
SNB-75	86.34
U251	103.84
**Melanoma**	
LOX IMVI	97.02
MALME-3M	94.58
M14	100.55
MDA-MB-435	94.64
SK-MEL-2	100.45
SK-MEL-28	99.44
SK-MEL-5	91.93
UACC-257	89.70
UACC-62	80.44
**Ovarian Cancer**	
IGROV1	92.88
OVCAR-3	103.85
OVCAR-5	94.72
OVCAR-8	96.65
NCI/ADR-RES	100.55
SK-OV-3	101.26
**Renal Cancer**	
786-0	102.13
ACHN	96.84
CAKI-1	83.71
RXF 393	99.10
SN12C	94.11
TK-10	100.73
UO-31	81.92
**Prostate Cancer**	
PC-3	92.63
DU-145	102.43
**Breast Cancer**	
MCF7	98.51
MDA-MB-231/ATCC	91.48
HS 578T	92.43
BT-549	103.66
T-47D	86.35
MDA-MB-468	97.74
Mean	96.21
Delta	21.10
Range	40.53

Mean Growth Percent - Growth Percent

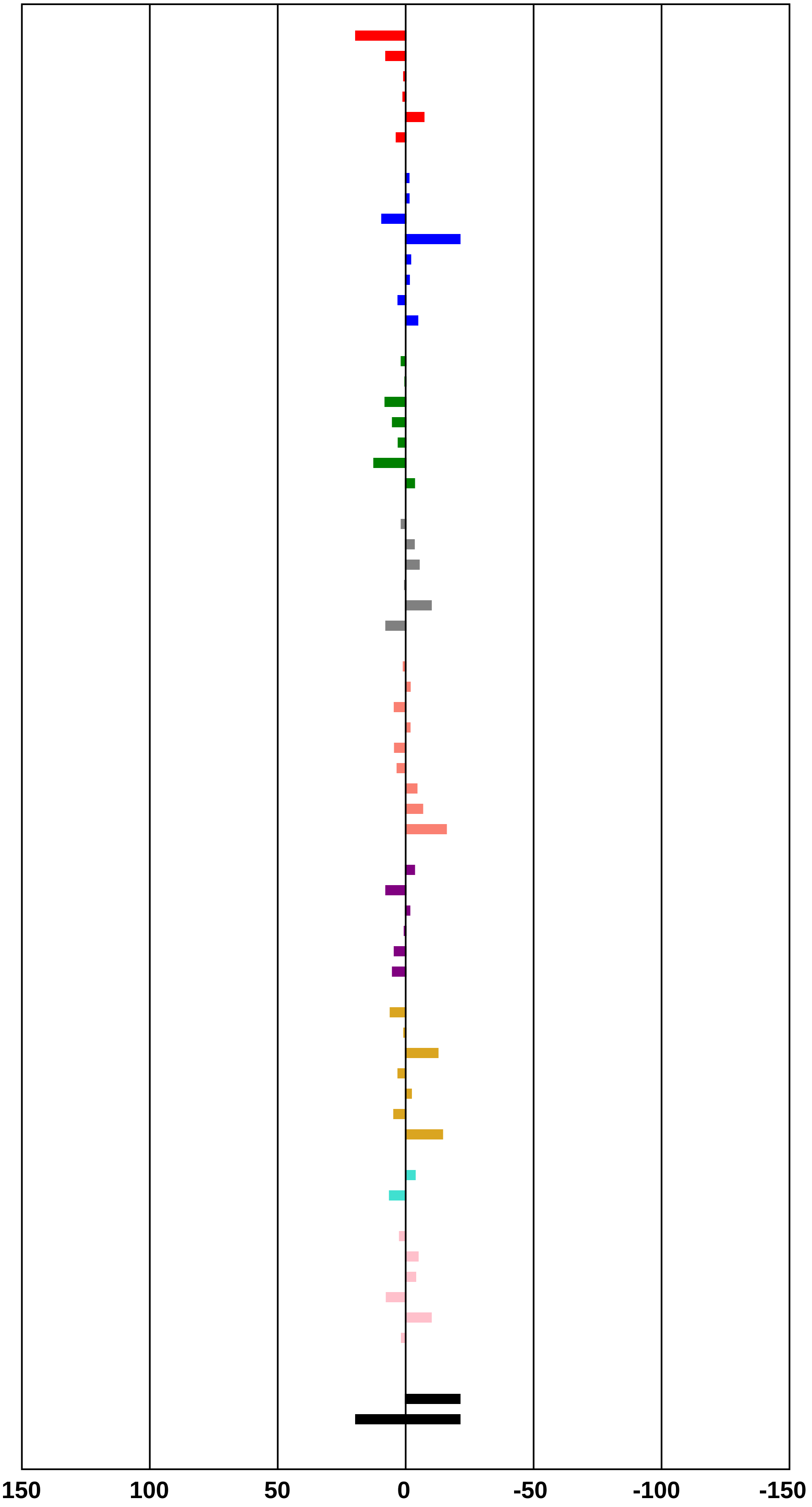

Developmental Therapeutics Program	NSC: D-815442 / 1	Conc: 1.00E-5 Molar	Test Date: May 20, 2019
One Dose Mean Graph 10d	Experiment ID: 1905OS11		Report Date: Jun 16, 2019

Panel/Cell Line	Growth Percent
Leukemia	
CCRF-CEM	113.66
HL-60(TB)	101.60
K-562	95.18
MOLT-4	101.80
RPMI-8226	101.77
SR	96.36
Non-Small Cell Lung Cancer	
A549/ATCC	97.69
EKVX	101.97
HOP-62	109.39
HOP-92	112.69
NCI-H23	101.18
NCI-H322M	100.79
NCI-H460	99.25
NCI-H522	87.56
Colon Cancer	
COLO 205	112.99
HCC-2998	107.09
HCT-116	101.99
HCT-15	104.92
HT29	108.26
KM12	104.35
SW-620	104.17
CNS Cancer	
SF-268	105.30
SF-295	101.24
SF-539	101.06
SNB-19	98.40
SNB-75	50.73
U251	100.19
Melanoma	
LOX IMVI	100.20
MALME-3M	106.42
M14	108.48
MDA-MB-435	95.07
SK-MEL-2	108.39
SK-MEL-28	100.77
SK-MEL-5	94.81
UACC-257	87.46
UACC-62	99.75
Ovarian Cancer	
IGROV1	104.75
OVCAR-3	111.15
OVCAR-5	98.14
OVCAR-8	101.79
NCI/ADR-RES	104.46
SK-OV-3	102.55
Renal Cancer	
786-0	102.28
ACHN	103.36
CAKI-1	88.79
RXF 393	107.02
SN12C	97.07
TK-10	101.97
UO-31	91.19
Prostate Cancer	
PC-3	103.80
DU-145	107.72
Breast Cancer	
MCF7	101.24
MDA-MB-231/ATCC	106.47
HS 578T	101.50
BT-549	92.23
T-47D	94.97
MDA-MB-468	102.63
Mean	100.84
Delta	50.11
Range	62.93

Mean Growth Percent - Growth Percent

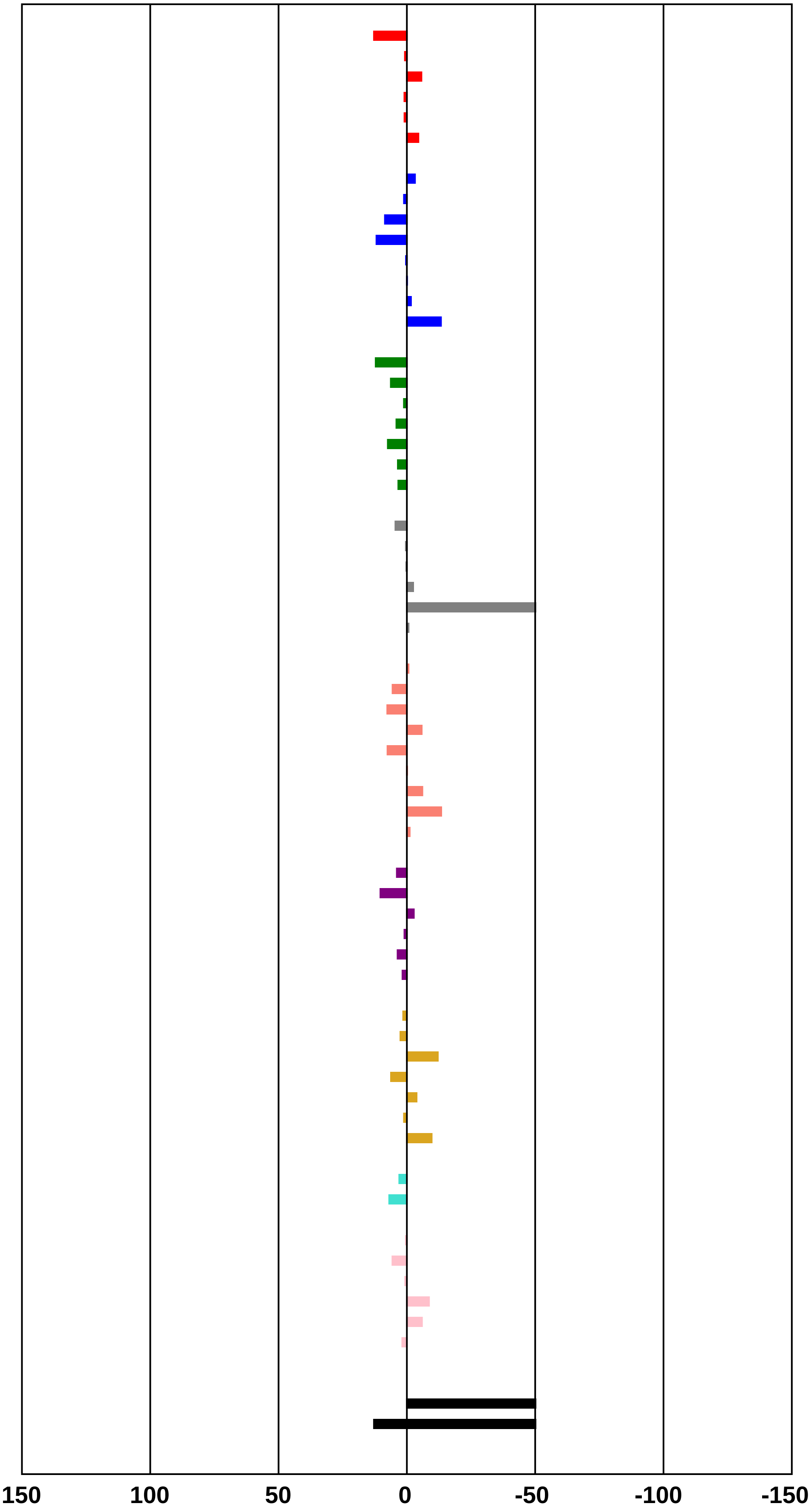

Supplement: Supplemental Material [file IENZ_A_1697250_SM2395.pdf]
